# NMR Structural Elucidation of Mitoxantrone–Gonadotropin-Releasing Hormone (GnRH) Conjugates Implicated in Hormone-Dependent Cancer

**DOI:** 10.3390/ijms27167437

**Published:** 2026-08-20

**Authors:** Georgia Biniari, Haralambos Tzoupis, Uroš Javornik, Nikitas Georgiou, Georgios Liapakis, Thomas Mavromoustakos, Theodore Tselios, Carmen Simal

**Affiliations:** 1 Department of Chemistry, University of Patras, 26504 Rion Patras, Greece; gbiniari@ac.upatras.gr (G.B.); haralambostz@gmail.com (H.T.); ttselios@upatras.gr (T.T.); 2 Slovenian NMR Centre, National Institute of Chemistry, SI-1001 Ljubljana, Slovenia; uros.javornik@ki.si; 3 Department of Chemistry, National and Kapodistrian University of Athens, 10679 Athens, Greece; nikitage@chem.uoa.gr (N.G.); tmavrom@chem.uoa.gr (T.M.); 4 Center for Interdisciplinary Biosciences, Technology and Innovation Park, P.J. Safarik University in Kosice, Jesenna 5, 04001 Kosice, Slovakia; 5 Department of Pharmacology, School of Medicine, University of Crete, 71003 Heraklion, Greece; liapakig@uoc.gr

**Keywords:** Gonadotropin Releasing Hormone (GnRH), GnRH receptor, mitoxantrone, peptide drug conjugates (PDCs), NMR spectroscopy, MD simulations, structure elucidation, targeted cancer therapy

## Abstract

Gonadotropin-Releasing Hormone receptors (GnRHRs) are overexpressed in several hormone-dependent malignancies, making them attractive molecular targets for selective anticancer drug delivery. Peptide–drug conjugates (PDCs) are a promising therapy for cancer and autoimmune diseases with high specificity and reduced toxicity. In this study, the three-dimensional structures of two previously synthesized mitoxantrone–GnRH conjugates, con3 and con7, were elucidated using high-resolution NMR spectroscopy in combination with molecular dynamics (MD) simulations. Complete ^1^H and ^13^C resonance assignments were achieved in DMSO-*d_6_* through two-dimensional NMR experiments. NOESY-derived distance restraints were subsequently used to refine the conformational ensembles obtained from MD simulations performed in water and DMSO. Both conjugates exhibited compact bent conformations with a U-shaped peptide backbone. The mitoxantrone moiety is positioned close to the peptide backbone in water simulations and NMR-refined structures, while it is positioned farther away in DMSO, without affecting the orientation of key residues involved in GnRH receptor binding. Importantly, His^2^, Trp^3^, and Arg^8^ remain solvent-exposed, whereas the disulfide bond is easily accessible to the solvent, consistent with the proposed drug release mechanism by the thioredoxin system. NMR-restrained molecular modeling confirmed the dominant conformational features predicted by the unconstrained theoretical simulations. Overall, these findings provide better structural understanding of the molecular organization of mitoxantrone–GnRH conjugates, highlighting key receptor-recognition residues and supporting both the proposed thioredoxin-mediated drug release mechanism and their previously reported biological properties. These insights may facilitate the rational design and optimization of improved GnRH peptide–drug conjugates for targeted therapy.

## 1. Introduction

Cancer is a multifactorial disease characterized by genetic alterations that disrupt the natural balance between cell proliferation and apoptosis [1,2,3,4]. Hormone-dependent cancers comprise a diverse group that affects thousands of individuals and includes two of the most common types of cancer in men (prostate cancer) and women (breast cancer) [5,6,7,8]. Steroid and peptide hormones play a pivotal role in regulating cell proliferation through endocrine, paracrine, or autocrine action [9,10,11]. Any disruption of these regulatory processes can lead to carcinogenesis.

Sex steroid hormones, such as estrogens and androgens, have been strongly associated with the development of hormone-dependent malignancies, including breast, ovarian, endometrial, and prostate cancers in both women and men [12]. Similarly, peptide hormones such as Gonadotropin-Releasing Hormone (GnRH) have also been implicated in cancer development [13,14,15]. Receptors for GnRH, which have a significant role in regulating reproductive function and sex steroid production, are overexpressed in certain cancer types. Consequently, their expression on the cell surface marks them as potential targets for therapeutic intervention [13,16,17,18,19].

The most common approaches involved the development of altered GnRH peptide analogues acting as agonists (e.g., Leuprolide) or antagonists (e.g., degarelix) or non-peptide antagonists (e.g., elagolix) [13,20,21,22,23,24]. However, the serious adverse effects associated with these therapies prompted the development of novel therapeutic strategies that incorporated cytotoxic agents with the GnRH peptide for targeted drug delivery. These peptide–drug conjugates (PDCs) represent a promising alternative for cancer therapy, providing enhanced selectivity and efficacy while minimizing systemic toxicity [25,26]. The first such conjugate, developed and synthesized by A. R. Schally and co-workers, was the AEZS-108 (Zoptrex), in which doxorubicin (DOX) is attached to a GnRH analogue [DLys^6^]GnRH via a glutaryl spacer [27]. Despite the encouraging outcomes from the initial clinical studies, AEZS-108 was discontinued after Phase III trials failed to demonstrate enhanced survival compared with doxorubicin. This outcome was attributed to the early release of doxorubicin into the bloodstream, highlighting the insufficient stability of the conjugate [28].

**Figure 1:**
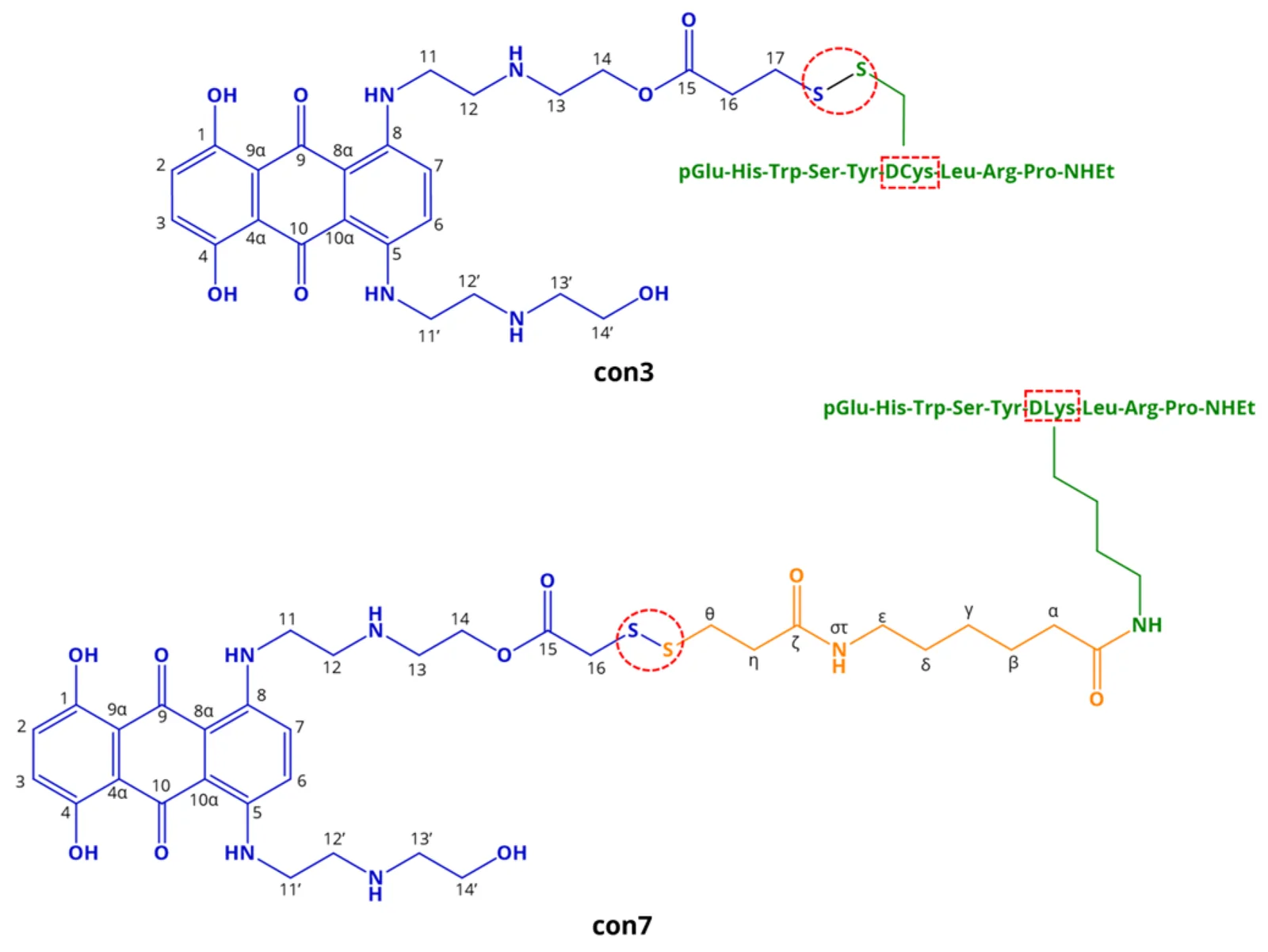
Structures of conjugates con3 and con7 with atom numbering used for NMR assignments. The mitoxantrone moiety (MTX) is shown in blue, the spacer in orange, and the GnRH peptide analogue in green. The red rectangle highlights the amino acid substitution between the two peptide sequences, while the red circle indicates the disulfide linkage connecting the peptide to the mitoxantrone moiety.

In this study, we present the structural elucidation of two comparable conjugates based on the structure of AEZS-108. These conjugates consist of a GnRH peptide analogue that is connected to mitoxantrone through a disulfide bond (Figure 1). The specific design aims to enhance the selectivity and efficacy of the cytotoxic agent while improving the stability of the conjugate. The GnRH peptide analogues were designed to be structurally similar to the commercially available agonist Leuprolide, except at position 6, where D-Leu was replaced by D-Lys or D-Cys [29]. Drug release is facilitated through a thioredoxin-mediated reductive mechanism. Mitoxantrone (MTX) was selected due to its improved toxicity profile compared with doxorubicin [29,30].

Both conjugates were evaluated in vitro and exhibited high affinity for the GnRH receptor. Moreover, they induced dose- and time-dependent anti-proliferative effects in different cancer cell lines, comparable to those of mitoxantrone alone. The findings demonstrate effective GnRH receptor targeting treatment with selectivity and suggest that the therapeutic benefits of these conjugates may arise from improved pharmacokinetics and reduced systemic toxicity [29,31,32]. Despite these promising biological results, the three-dimensional solution structures and the impact of mitoxantrone conjugation on the conformational properties of the GnRH peptide analogue remained unknown. Such structural characterization is essential for understanding receptor recognition and guiding the rational design of improved GnRH peptide–drug conjugates. To address this knowledge gap, the conformational properties of con3 and con7 were investigated by two-dimensional NMR spectroscopy combined with molecular dynamics simulations to obtain solution-state structural models and provide insights into conjugate–GnRH receptor interactions.

## 2. Results and Discussion

### 2.1. Structure Identification

Initially, NMR experiments of the synthesized analogues were performed in D_2_O (Tables S1, S3, S5 and S6), as it mimics the aqueous intracellular environment. However, these spectra proved unsuitable for complete ^1^H assignment and structural elucidation because rapidly exchanging protons were not observable due to their exchange with deuterium from the solvent. To overcome this limitation, the chemical shift assignments of the two conjugates, con3 (Table 1), con7 (Table 2), and their respective peptides (Tables S2 and S4), were carried out in DMSO-*d_6_*. This solvent provides an amphiphilic environment that suppresses proton–deuterium exchange, allowing the observation of exchangeable protons. The assignments of con3 and con7 in DMSO-*d_6_* were achieved using a combination of ^1^H NMR, ^1^H-^1^H NOESY, and ^1^H-^15^N HSQC experiments, together with data from the corresponding peptide spectra in DMSO-*d_6_* and comparative information obtained from the D_2_O NMR experiments.

### 2.2. Structure Elucidation

Cross-peaks in the NOESY spectra of all analogues corresponding to spatial proximity between non-overlapping proton resonances were analyzed. The analysis started on the spectral regions between 0.8 and 2.0 ppm (aliphatic protons) and 6.0–8.0 ppm (aromatic protons), which were examined in detail to identify well-resolved, non-overlapping cross-peaks suitable for use as distance restraints. The analysis was then extended to the remaining spectral regions. The complete lists of observed cross-peaks are provided in Table 3 for con3 and con7, and in Table S7 for the corresponding peptides; the respective distance restraints are reported in the Supplementary Materials (Tables S9–S12).

### 2.3. MD in Water and DMSO

**Figure 2:**
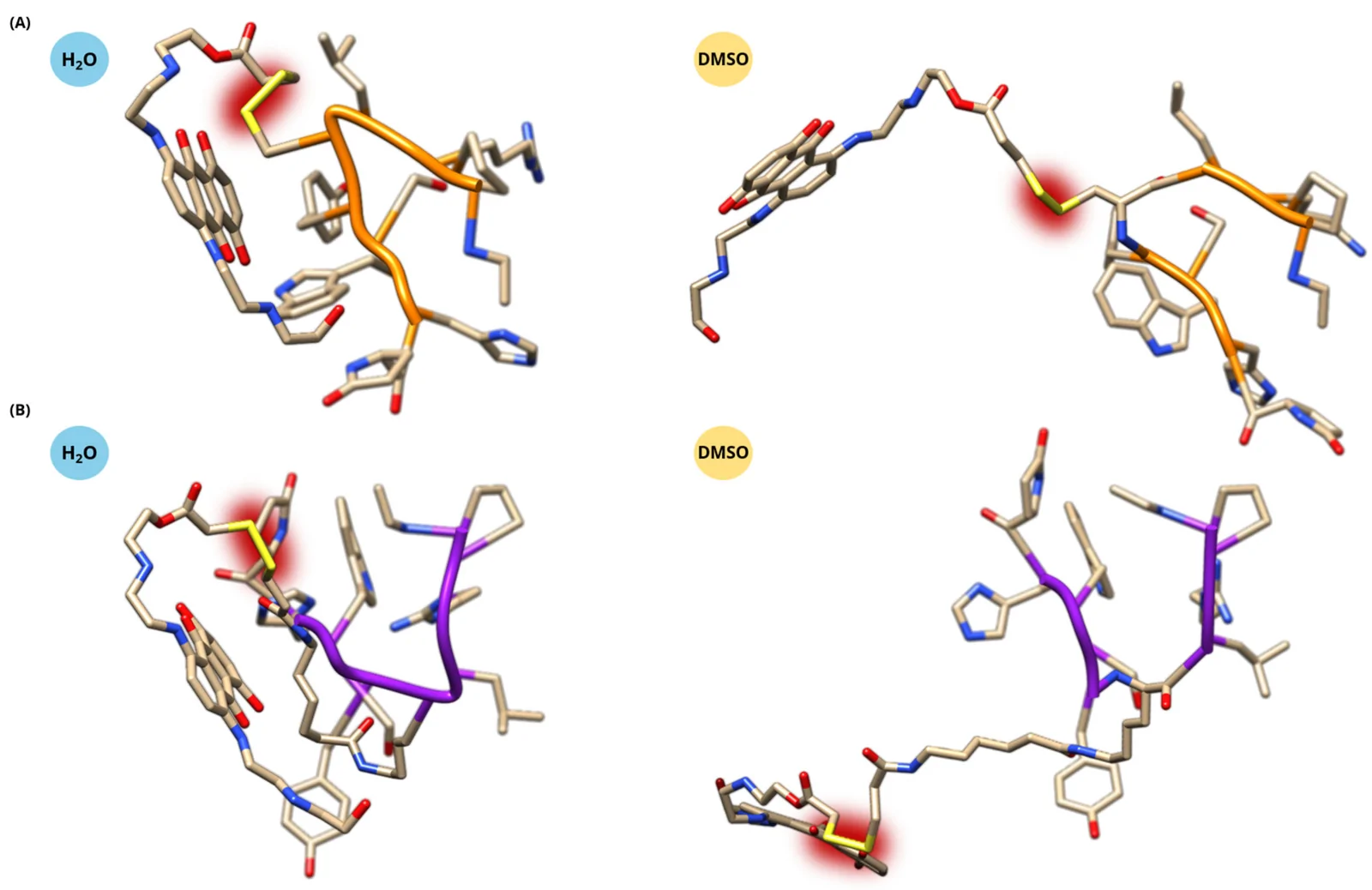
Dominant conformations of con3 (**A**) and con7 (**B**) from MD in water and DMSO. The peptide backbone adopts a U-shaped conformation ((**A**): orange; (**B**): purple), positioning the mitoxantrone moiety close to the peptide in H_2_O simulations but farther away in DMSO simulations. In both simulation environments, the disulfide bond (red highlighting) is exposed to the solvent. Atoms are colored as follows: sulfur (S) in yellow, oxygen (O) in red, and nitrogen (N) in dark blue.

Clustering analysis in water identified two dominant clusters for each conjugate, representing 50% (Figure 2A) and 19% of structures in the simulation time for con3, and 45% (Figure 2B) and 25% for con7, respectively. The dominant clusters overall exhibited highly similar conformations, with only minor differences in the orientation of the mitoxantrone tricyclic moiety. In all clusters, the peptide backbone retained the U-shaped conformation, indicating that the conformation of conjugates depends mainly on the peptide backbone rather than the mitoxantrone moiety. In contrast, analysis in DMSO revealed a single dominant cluster for each conjugate, which was present for approximately 85% of the simulation time (Figure 2).

In con3 (Figure 2A), the peptide backbone adopts a closed U-shaped conformation instead of the helical structure observed for the free peptide (Figure S3A). On the other hand, the peptide backbone of con7 (Figure 2B) adopts a more compact U-shaped conformation than its corresponding free peptide (Figure S3B). The mitoxantrone moiety remains in close proximity to the peptide backbone in both conjugates during the water simulations but is positioned farther away in DMSO (Figure 2). In both solvents, the disulfide bond is exposed to the solvent, facilitating its proposed reduction mechanism by the thioredoxin system. Previous structural studies of Leuprolide and GnRH peptide analogues have identified His^2^, Trp^3^ and Arg^8^ as key residues involved in receptor recognition [33]. In the conformations observed here, the side chains of these amino acids are exposed to the solvent (Figure S3 and Table S8), potentially enhancing receptor interactions and facilitating GnRHR activation.

### 2.4. MD with NOE Constraints

**Figure 3:**
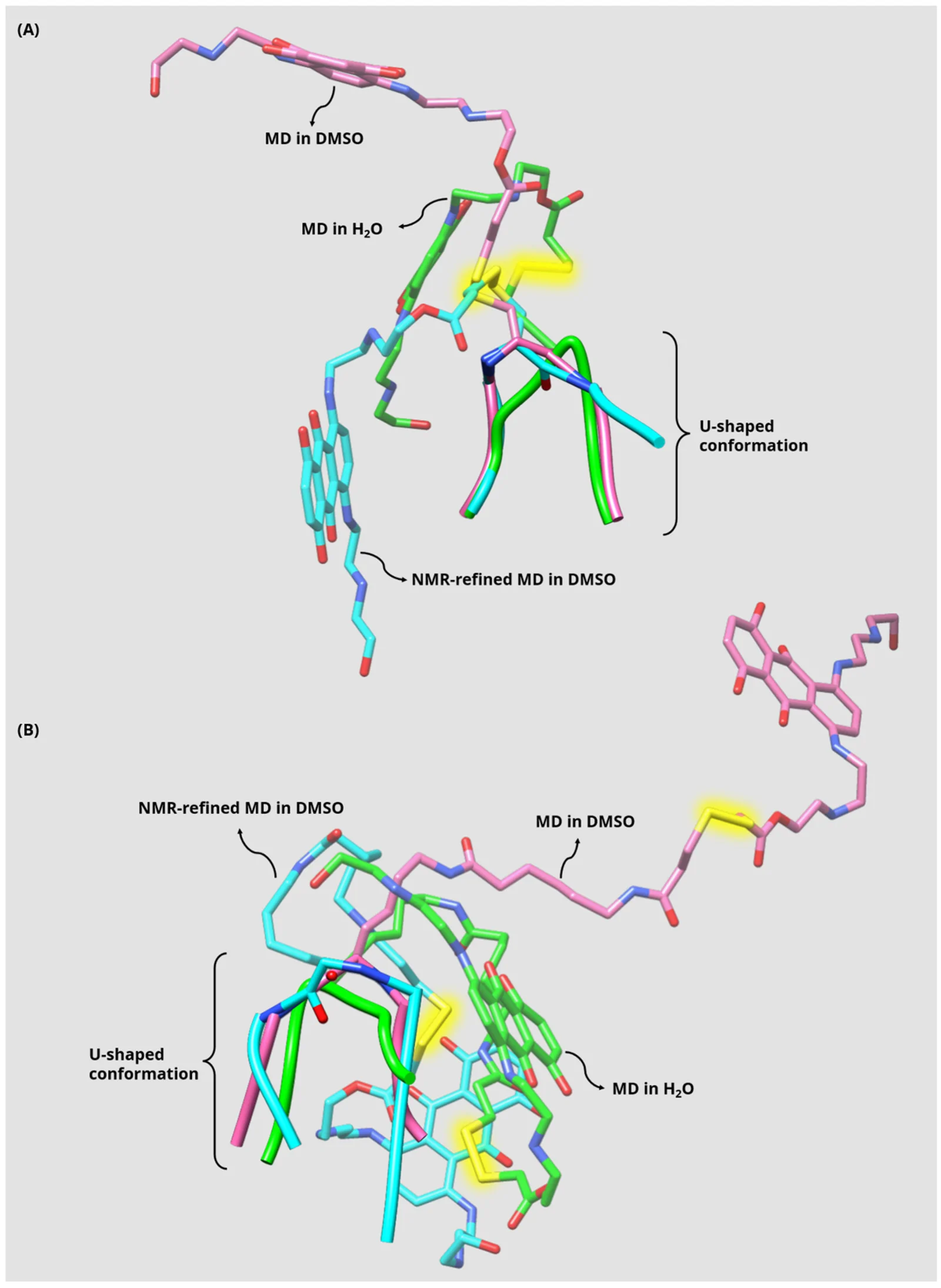
Structural comparison of con3 (**A**) and con7 (**B**) obtained from MD simulations in H_2_O (green), DMSO (pink), and NMR-refined MD simulations in DMSO (cyan). For clarity, amino acid side chains are omitted. In the theoretical MD simulations (green and pink), the peptide backbone adopts a compact U-shaped conformation, whereas the NMR-refined structures (cyan) appear slightly more open. This difference may arise from the limited number of experimentally derived NOE distance restraints available for structure refinement. In all structures, the disulfide bond (yellow highlighting) remains exposed to the solvent, whereas the mitoxantrone moiety is close to the peptide backbone, only in water simulations. For the NMR-refined structures in DMSO, mitoxantrone is located close to the peptide in both conjugates. The atoms are colored as follows: sulfur (S) in yellow, oxygen (O) in red, and nitrogen (N) in dark blue.

The NMR refinement, performed using the NMR experimental constraints (Table 3 and Table S7), yielded the predicted structures for conjugates in Figure 3 and for the respective peptides in Figure S3. The dominant conformations from the unrestrained MD simulations were used as the starting structures for the refinement protocol. In both the peptides (Figure S3) and the conjugates (Figure 3), the peptide backbone retains a bent conformation. In the peptide component of con7 (Figure S3B), the backbone adopts an almost helical conformation, while in all other cases the conformations mostly resemble a U-shape (Figure S4B). Another notable feature observed in the two conjugates is the compact conformation of the mitoxantrone moiety (Figure S4). In the NMR-refined structures of both conjugates, the drug is positioned in close proximity to the peptide backbone, while the spacer in con7 (Figure S4B) is folded onto itself. Furthermore, the spacer conformation exposes the disulfide bond to the solvent, potentially facilitating its reduction, as noted above. Also, the side chains of His^2^, Trp^3^, and Arg^8^ exhibit a solvent-exposed orientation in both conjugates (Figure S3), which is consistent with the high GnRHR binding affinity observed in our previously reported study [29]. The main limitation of NMR refinement modeling is the incomplete definition of all the NOE constraints. Extensive signal overlap and a possible compact conformation of the peptide in solution hindered the assignment of NMR signals for every single proton; consequently, undetermined NOE constraints could not be considered in the refinement.

The comparison between the unconstrained MD simulations in water and DMSO and the NOE-restrained simulations revealed a high degree of structural agreement. In all structures, the peptide backbone adopted a bent, U-shaped conformation, while the mitoxantrone moiety remained away from the peptide backbone only in the DMSO MD simulations. The NMR-refined structures had slightly more open conformations than those obtained from the corresponding MD simulations due to the limited number of NOE restraints available for refinement. Nevertheless, the major structural features were consistently reproduced by both the computational and experimental approaches. Importantly, in all structures the disulfide bond remained solvent-exposed, indicating that it is accessible to the thioredoxin system for an effective release of the mitoxantrone moiety. This structural observation is compatible with the biological activity of the conjugates and further supports the proposed thioredoxin-mediated drug-release mechanism [29,31,32].

### 2.5. Docking

**Figure 4:**
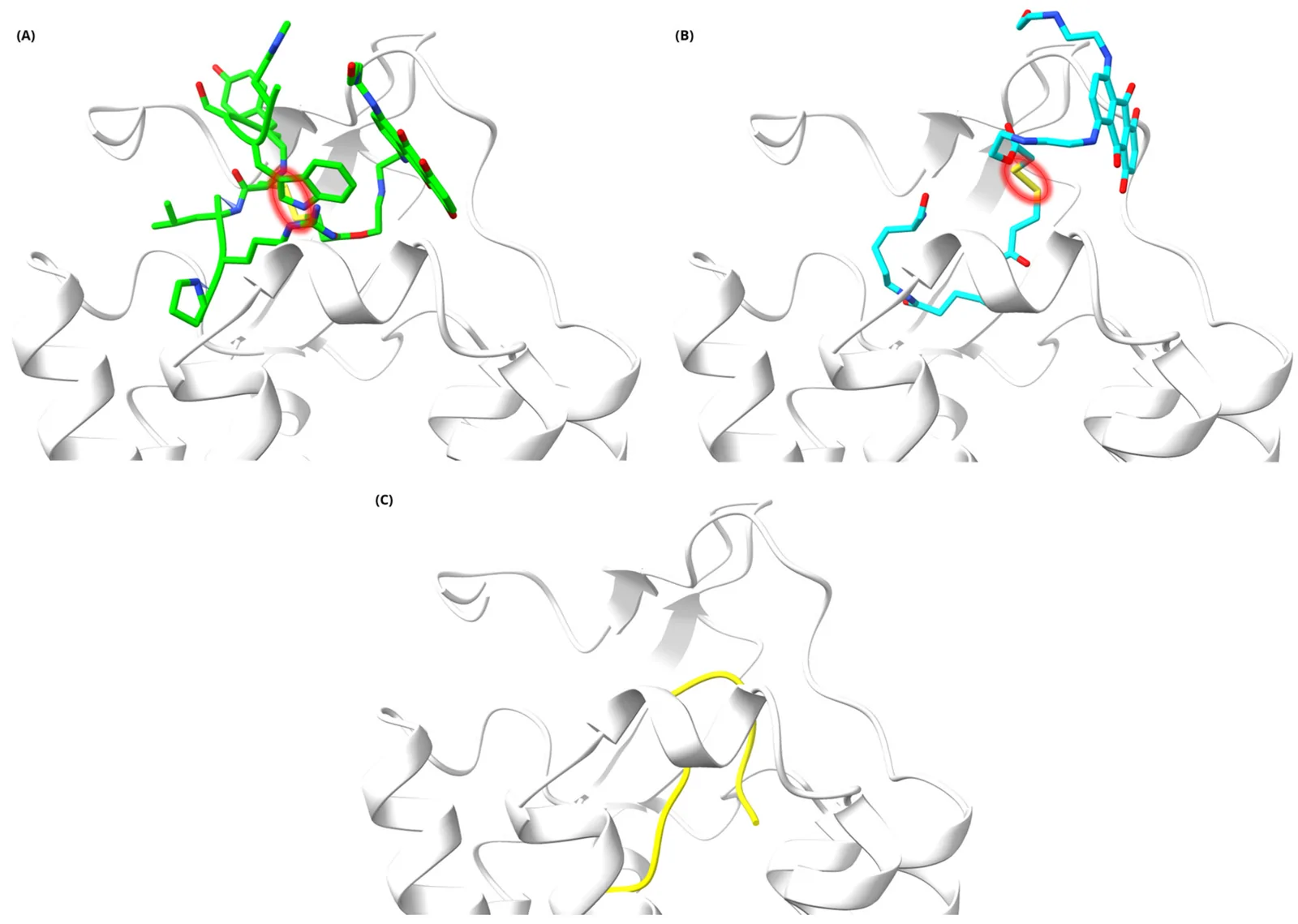
Orientation of (**A**) con3, (**B**) con7 and (**C**) Leuprolide as predicted by molecular docking simulations. In both conjugates, the mitoxantrone moiety and disulfide bond are exposed to the solvent. The Leuprolide binding site is slightly deeper than that of the conjugates.

The molecular docking simulations were performed using the conformations obtained after the NMR refinement. The results are summarized in Table 4 and in Figure 4. Due to their size, the peptide conjugates dock in a vertical fashion and do not move deeply into the receptor-binding site like Leuprolide (Figure 4C). This binding mode potentially resembles the dynamic process of GnRH recognition, in which the peptide approaches the receptor from the extracellular side. Based on the analysis of the docking conformations, there are no hydrogen bonds formed between the peptides and the protein, suggesting that binding is predominantly stabilized by nonpolar interactions such as van der Waals interactions. Notably, the docking results are consistent with the solution conformations obtained from MD simulations in water and NMR-guided MD simulations, indicating that these conformations are compatible with GnRHR recognition while allowing distinct orientations of the mitoxantrone moiety upon receptor binding.

Importantly, docking results of these analogues compared with Leuprolide suggest that substitution of the D-Leu^6^ residue with either D-Cys or D-Lys is well tolerated within the GnRH receptor binding pocket. Although comparisons of docking scores should be interpreted with caution, the predicted binding modes are consistent with the ability of these conjugates to interact with the GnRH receptor, as demonstrated in previous experimental studies [29,31,32]. Con7 exhibited a docking score of −13.201 kcal/mol, which was more favorable than that of the reference agonist Leuprolide (−12.335 kcal/mol) and agrees with our earlier work [29]. In contrast, con3 exhibited a less favorable docking score (−9.869 kcal/mol), despite its previously reported high experimental binding affinity [29], further indicating the limitations of these calculated values. The docking score of con7 was comparable to that of its corresponding free peptide (−13.292 kcal/mol), suggesting that the presence of the spacer and the bulky mitoxantrone moiety at position 6 does not prevent peptide–receptor recognition in the predicted binding model. Con3 exhibited a lower docking score than its corresponding free peptide (−13.494 kcal/mol), which may be related to the absence of the spacer and the shorter D-Cys side chain, resulting in lower conformational flexibility. As shown in Figure 4C, Leuprolide binds deeper within the receptor pocket than either conjugate. In both conjugates, the mitoxantrone moiety and the disulfide bond (Figure 4A,B) remain solvent-exposed and can easily be accessed by the thioredoxin system for reduction, which is consistent with our previous findings [29]. The disulfide bond of con3 is positioned slightly deeper within the binding pocket, whereas the flexible spacer of con7 allows greater solvent exposure, potentially facilitating its reduction.

## 3. Materials and Methods

### 3.1. Synthesis of con3 and con7

The synthesis of conjugates con3 and con7 has been described in our previous work [29]. Briefly, the peptides were synthesized using the Fmoc/tBu solid-phase methodology, and conjugation to the mitoxantrone analogue was achieved by disulfide bond formation. The final products were purified by reverse-phase high-performance liquid chromatography (RP-HPLC) (purity > 95%) and characterized by electrospray ionization mass spectrometry (ESI-MS).

### 3.2. NMR Spectroscopy

DMSO-*d_6_* (99.8 atom% D) was purchased from Deutero GmbH (Kastellaun, Germany). High-precision 5 mm NMR tubes (Deutero GmbH) were used throughout the study. High-resolution NMR spectra were recorded at 298 K on a Bruker Avance Neo 800 MHz spectrometer equipped with a cryogenically cooled 5 mm ^1^H/^13^C/^15^N/D z-gradient probe. All experiments were acquired in phase-sensitive mode using standard pulse sequences and phase-cycling routines provided in the Bruker pulse program library. Detailed descriptions of the NMR experiments are provided in the Supplementary Materials (Section S2) and NMR spectra in Figures S6–S45. The NMR experiments were performed over three consecutive days, with the samples maintained in DMSO throughout this period. Sample integrity after completion of the NMR experiments was confirmed by RP-HPLC and ESI-MS, with no detectable degradation of either conjugate. Raw NMR data were processed and analyzed using MestReNova software (version 14.3.3-33362) [34].

#### 3.2.1. NMR Resonance Assignment

Structure identification of the conjugates con3 (Table 1), con7 (Table 2), and their respective peptides (Supplementary Materials; Section S3) through NMR experiments was performed in DMSO-*d_6_* under the conditions described in Supplementary Materials; Section S2. Chemical shift assignments were obtained from ^1^H NMR, ^13^C NMR, 2D ^1^H-^1^H NOESY, and 2D ^1^H-^15^N HSQC spectra. Additionally, data from the assignment of all analogues in D_2_O (Supplementary Materials; Sections S1 and S3) were used to support the assignment in DMSO-*d_6_*.

#### 3.2.2. NOESY Analysis and Distance Restraints

The structure elucidation of conjugates con3 and con7 was achieved using two-dimensional ^1^H-^1^H NOESY experiments. Cross-peaks corresponding to spatial proximity between non-overlapping proton resonances were analyzed. The resulting distance restraints were incorporated into molecular dynamics (MD) simulations to refine the structural models, using a common upper-distance limit of 5.5 Å for all assigned cross-peaks. The cross-peaks observed in the NOESY spectra of con3 and con7 are shown in Table 3, and those of the respective peptides in Table S7, while the distance restraints used for the simulations are provided in Tables S9–S12.

### 3.3. Molecular Modeling

Molecular dynamics (MD) simulations were initially performed in water to evaluate the conformational aspects of the conjugates under physiological conditions. However, since NMR structural assignments could only be achieved in DMSO-*d_6_*, MD simulations were also carried out in DMSO to enable the direct comparison between the computational models and the NMR experiments. MD production runs were conducted in water and DMSO solvents with and without constraints. The simulation run was carried out for 150 ns using AMBER22 software [35]. Structure preparation: The 2D structures of the analogues were designed with UCSF Chimera 1.16 [36]. Geometry optimization was performed with the GAMESS R1 [37,38] software using the density-functional theory (DFT) [39,40] and the Hartree–Fock (HF) approximation. The B3LYP/6-311G atomic basis set was applied, while the convergence criterion was set at 0.0001 [40,41]. Detailed information on the construction of the parameters and the MD protocol followed is provided in the Supplementary Materials (Section S6).

### 3.4. Molecular Docking Protocol

The structures obtained from the NMR refinement process (Supplementary Materials Section S5) were employed for molecular docking simulations using Autodock Vina [3.1] [42,43]. The conjugates were treated as a single ligand and prepared with the prepare_ligand tool in the software. The GnRH receptor 1 AlphaFold canonical structure (AF-P30968-F1, Uniprot ID: P30968) was employed for the docking due to the absence of any missing residues compared to the crystal structure (Figure S5) and was prepared using the prepare_receptor tool provided by Autodock Vina. The structure of Leuprolide was obtained from the Protein Data Bank Chemical Component Dictionary (PDB ID: 1YY2) and used as the reference ligand for comparison. The binding site of the receptor was defined based on the center-of-mass of elagolix (Figure S5) with coordinates (x, y, z) = (−26.906, −13.2388, 2.45162) and a box size of 35 × 40 × 39 Å. For the docking protocol, an exhaustiveness value of 32 was used.

## 4. Conclusions

The conformations of the two synthesized mitoxantrone–GnRH conjugates, con3 and con7, were evaluated by high-resolution NMR spectroscopy combined with molecular dynamics simulations in DMSO and water. ^1^H and ^13^C assignments were achieved in both solvents, and NOESY-derived distance restraints provided valuable structural information that was incorporated into the MD refinement protocols. Computational and experimental data indicate that both conjugates adopt compact bent conformations characterized by a U-shaped arrangement of the peptide backbone. Despite the sequence difference at position 6, both conjugates exhibit similar conformations, suggesting that this substitution has only a limited influence on the global structure. The mitoxantrone moiety does not significantly alter the peptide backbone conformation, while the key residues His^2^, Trp^3^, and Arg^8^ remain exposed to the solvent, thereby preserving their ability to interact with the GnRH receptor. Moreover, the orientation of the mitoxantrone moiety exposes the disulfide bond to the solvent, which is consistent with the proposed mechanism of intracellular controlled drug release mediated by the thioredoxin system. The conformational characteristics observed in the unconstrained simulations were further confirmed by NMR-restrained molecular modeling, which generated refined structural models consistent with experimental NOE data. A limitation of the present NMR study was the inability to assign all observed NOE cross-peaks due to signal overlap; therefore, only confidently assigned NOE-derived distance restraints were included in the refinement protocols. Nevertheless, the resulting structural models provide a reliable representation of the predominant solution conformations of both conjugates. The docking studies showed that the structural models of the experimentally determined conformations are compatible with GnRH receptor recognition. Combined with the NMR spectroscopy and molecular dynamics results, these findings provide a framework that explains the previously reported biological evaluation of these conjugates [29,31,32]. In particular, the preservation of the receptor-recognition residues is consistent with the previously reported interaction of these conjugates with the GnRH receptor, and the solvent-exposed disulfide bond further supports the proposed thioredoxin-mediated drug-release mechanism. Moreover, the docking model of con7 suggests that the flexible spacer may facilitate its accommodation within the receptor-binding site while maintaining accessibility of the disulfide bond. Overall, the structural information obtained in this study enhances our understanding of GnRH receptor recognition and supports the rational design and optimization of next-generation GnRH peptide–drug conjugates and their continued development as selective drug-delivery platforms for GnRH receptor–expressing malignancies.

## Figures and Tables

**Table 1:** ^1^H chemical shift assignment of conjugate con3 (800 MHz, DMSO-*d_6_*, 298 K). Chemical shifts are referenced to DMSO-*d_6_* (δ^1^H = 2.50).

Residue chemical shift				
	NH	αH	βH	Others
pGlu^1^ (pGlu)	7.71	3.98	2.15	γH: 1.72
His^2^ (H)	8.09	4.53	2.85, 2.98	2H: 9.21, 5H: 6.55
Trp^3^ (W)	8.06	4.63	2.98, 3.16	1H: 10.79; 2H: 7.13; 4H: 7.60; 5H: 6.91; 6H: 7.04; 7H: 7.31
Ser^4^ (S)	8.34	4.34	3.52, 3.58	-
Tyr^5^ (Y)	7.99	4.50	2.72, 2.97	2, 6H: 7.05; 3, 5H: 6.63
DCys^6^ (DC)	8.42	4.59	2.84, 3.03	-
Leu^7^ (L)	8.24	4.34	1.55	γH: 1.43; δH: 0.83; δ^′^H: 0.86
Arg^8^ (R)	8.14	4.45	1.72	γH: 1.55; δH: 3.09; εH: 7.58
Pro^9^ (P)	-	4.21	2.01	γH: 1.79, 1.91; δH: 3.52, 3.66
NHEt^10^ (NHEt)	7.81	3.04	0.98	-
**Mitoxantrone Analogue**				
1, 4OH			13.49	
2, 3H			7.23	
5, 8NH			10.46	
6, 7H			7.59	
11H			3.84	
11′H			3.67	
12H			3.27	
12′H			3.08	
13H			3.27	
13′H			3.27	
14H			4.29	
14′H			3.87	
16H			3.16	
17H			2.76	

**Table 2:** ^1^H chemical shift assignment of conjugate con7 (800 MHz, DMSO-*d_6_*, 298 K). Chemical shifts are referenced to DMSO-*d_6_* (δ^1^H = 2.50).

Residue chemical shift				
	NH	αH	βH	Others
pGlu^1^ (pGlu)	7.69	3.97	2.14	γH: 1.71
His^2^ (H)	8.10	4.56	2.87, 2.99	2H: 9.18, 5H: 6.54
Trp^3^ (W)	8.06	4.62	2.97, 3.15	
Ser^4^ (S)	8.32	4.34	3.50, 3.58	-
Tyr^5^ (Y)	7.97	4.48	2.72, 2.90	2, 6H: 7.02; 3, 5H: 6.62
DLys^6^ (DK)	8.05	4.23	1.43, 1.53	γH: 1.09; δH: 1.31; εH: 2.94; στH: 7.70
Leu^7^ (L)	8.07	4.30	1.55	γH: 1.43; δH: 0.79; δ^′^H: 0.85
Arg^8^ (R)	8.09	4.45	1.72, 1.79	γH: 1.54; δH: 3.08; εH: 7.54
Pro^9^ (P)	-	4.20	2.00, 1.72	γH: 1.80; 1.90; δH: 3.51, 3.67
NHEt^10^ (NHEt)	7.80	2.99, 3.04	0.97	-
Ahx Spacer	-	2.00	1.34	γH: 1.19; δH: 1.44; εH: 2.92, 2.99; στH: 7.91; ηH: 2.45; θH: 2.71, 2.93
**Mitoxantrone Analogue**				
1, 4OH			13.47	
2, 3H			7.59	
5, 8NH			10.44	
6, 7H			7.23	
11H			3.86	
11′H			3.66	
12H			3.26	
12′H			3.22	
13H			3.07	
13′H			3.21	
14H			4.35	
14′H			3.86	
16H			3.69	

**Table 3:** Observed NOE connectivities for con7 and con3 based on NOESY spectra.

Correlations							
con3				con7			
NH*_W3_*	βH*_NHEt10_*	αH*_pGlu1_*	2H*_W3_*	NH*_S4_*	δH*_L7_*, δ’H*_L7_*	1, 4OH*_MTX_*	5H*_H2_*
NH*_Y5_*	δH*_L7_*, δ’H*_L7_*	αH*_DC6_*	δH*_L7_*, δ’H*_L7_*	NH*_S4_*	γH_D_*_K6_*	1, 4OH*_MTX_*	2H*_H2_*
NH*_Y5_*	γH*_R8_*	γH*_R8_*	αH*_P9_*	NH*_Y5_*	δH*_L7_*, δ’H*_L7_*	1, 4OH*_MTX_*	5, 8NH*_MTX_*
NH*_DC6_*	δH*_L7_*, δ’H*_L7_*	γH*_R8_*	14H*_MTX_*	NH*_Y5_*	γH_D_*_K6_*	1, 4OH*_MTX_*	2, 3H*_MTX_*
NH*_R8_*	δH*_L7_*, δ’H*_L7_*	αH*_P9_*	δH*_L7_*, δ’H*_L7_*	στH*_Spacer_*	δH*_L7_*, δ’H*_L7_*	16H*_MTX_*	5, 8NH*_MTX_*
2H*_W3_*	δH*_L7_*, δ’H*_L7_*	αH*_P9_*	βH*_NHEt10_*	στH*_Spacer_*	γH*_Spacer_*	16H*_MTX_*	στH*_Spacer_*
3,5H*_Y5_*	δH*_L7_*, δ’H*_L7_*	1, 4OH*_MTX_*	2H*_H2_*	στH*_Spacer_*	βH*_Spacer_*	16H*_MTX_*	2, 3H*_MTX_*
γH*_R8_*	NH*_pGlu1_*	1, 4OH*_MTX_*	5H*_H2_*	2H*_W3_*	δH*_L7_*, δ’H*_L7_*	16H*_MTX_*	ηH*_Spacer_*
γH*_R8_*	2H*_W3_*	1, 4OH*_MTX_*	2, 3H*_MTX_*	2H*_W3_*	γH_D_*_K6_*	γH*_Spacer_*	ηH*_Spacer_*
γH*_R8_*	4H*_W3_*	1, 4OH*_MTX_*	5, 8NH*_MTX_*	3, 5H*_Y5_*	δH*_L7_*, δ’H*_L7_*	βH*_Spacer_*	ηH*_Spacer_*
γH*_R8_*	7H*_W3_*	5, 8NH*_MTX_*	14H*_MTX_*	3, 5H*_Y5_*	γH_D_*_K6_*		
γH*_R8_*	NH*_NHEt10_*	17H*_MTX_*	NH*_L7_*	2H*_H2_*	3,5H*_Y5_*		
αH*_Y5_*	δH*_L7_*, δ’H*_L7_*	17H*_MTX_*	δH*_L7_*, δ’H*_L7_*	NH*_S4_*	NH*_Y5_*		

**Table 4:** Docking scores calculated with Autodock Vina. Values are in kcal/mol.

Analogue	Docking Score
Con3	−9.869
Peptide component of con3	−13.494
Con7	−13.201
Peptide component of con7	−13.292
Leuprolide	−12.335

## Data Availability

The original contributions presented in this study are included in the article/Supplementary Material. Further inquiries can be directed to the corresponding authors.

## Supplementary Materials

The following supporting information can be downloaded at: https://www.mdpi.com/article/10.3390/ijms27167437/s1.

## References

[1] Hanahan D. (2026). Hallmarks of cancer—Then and now, and beyond. Cell.

[2] Mustafa M., Ahmad R., Tantry I.Q., Ahmad W., Siddiqui S., Alam M., Abbas K., Moinuddin, Hassan I., Habib S. (2024). Apoptosis: A comprehensive overview of signaling pathways, morphological changes, and physiological significance and therapeutic implications. Cells.

[3] Bhat G.R., Sethi I., Sadida H.Q., Rah B., Mir R., Algehainy N., Albalawi I.A., Masoodi T., Subbaraj G.K., Jamal F. (2024). Cancer cell plasticity: From cellular, molecular, and genetic mechanisms to tumor heterogeneity and drug resistance. Cancer Metastasis Rev..

[4] Ruddon R.W. (2007). Cancer Biology.

[5] Tettey T.T., Rinaldi L., Hager G.L. (2023). Long-Range Gene Regulation in Hormone-Dependent Cancer. Nat. Rev. Cancer.

[6] Bergengren O., Pekala K.R., Matsoukas K., Fainberg J., Mungovan S.F., Bratt O., Bray F., Brawley O., Luckenbaugh A.N., Mucci L. (2023). 2022 Update on Prostate Cancer Epidemiology and Risk Factors—A Systematic Review. Eur. Urol..

[7] Arnold M., Morgan E., Rumgay H., Mafra A., Singh D., Laversanne M., Vignat J., Gralow J.R., Cardoso F., Siesling S. (2022). Current and Future Burden of Breast Cancer: Global Statistics for 2020 and 2040. Breast.

[8] Ulm M., Ramesh A.V., McNamara K.M., Ponnusamy S., Sasano H., Narayanan R. (2019). Therapeutic Advances in Hormone-Dependent Cancers: Focus on Prostate, Breast and Ovarian Cancers. Endocr. Connect..

[9] Zhang X., Pandey V., Bhardwaj V., Zhu T., Lobie P.E. (2024). Autocrine/Paracrine Growth Hormone in Cancer Progression. Endocr. Relat. Cancer.

[10] Husted A.S., Trauelsen M., Rudenko O., Hjorth S.A., Schwartz T.W. (2017). GPCR-Mediated Signaling of Metabolites. Cell Metab..

[11] Tzoupis H., Nteli A., Androutsou M.-E., Tselios T. (2020). Gonadotropin-Releasing Hormone and GnRH Receptor: Structure, Function and Drug Development. Curr. Med. Chem..

[12] Miki Y. (2023). Hormone-Dependent Cancers: New Aspects on Biochemistry and Molecular Pathology. Int. J. Mol. Sci..

[13] Garrido M.P., Hernandez A., Vega M., Araya E., Romero C. (2023). Conventional and new proposals of GnRH therapy for ovarian, breast, and prostatic cancers. Front. Endocrinol..

[14] Gründker C., Emons G. (2021). Role of Gonadotropin-Releasing Hormone (GnRH) in Ovarian Cancer. Cells.

[15] Sun Q., Zhao Z. (2017). Peptide Hormones as Tumor Markers in Clinical Practice. Enzymes.

[16] Liu Q., Sun J., Zhou X., Zhang M., Pu T., Gao X., Zhang M., Xu C., Zhang X. (2025). A Near-Infrared Fluorescent Probe for Specific Imaging of Lymph Node Metastases in Ovarian Cancer via Active Targeting of the Gonadotropin-Releasing Hormone Receptor. Biomolecules.

[17] Giorgio A., Varani M., Lauri C., Bentivoglio V., Nayak P. (2025). Radiolabeled LHRH and FSH Analogues as Cancer Theranostic Agents: A Systematic Review. J. Clin. Med..

[18] Gründker C., Emons G. (2017). The Role of Gonadotropin-Releasing Hormone in Cancer Cell Proliferation and Metastasis. Front. Endocrinol..

[19] Limonta P., Manea M. (2013). Gonadotropin-Releasing Hormone Receptors as Molecular Therapeutic Targets in Prostate Cancer: Current Options and Emerging Strategies. Cancer Treat. Rev..

[20] Barretta M., Vignali M., La Marca A., Grandi G. (2025). The oral GnRH antagonists, a new class of drugs in gynecology: From pharmacokinetics to possible clinical applications. Expert Opin. Drug Metab. Toxicol..

[21] Chwalisz K. (2023). Clinical development of the GnRH agonist leuprolide acetate depot. F&S Rep..

[22] Nsereko Y., Armstrong A., Coburn F., Al Musaimi O. (2025). Innovative Peptide Therapeutics in the Pipeline: Transforming Cancer Detection and Treatment. Int. J. Mol. Sci..

[23] Salciccia S., Gentilucci A., Cattarino S., Sciarra A. (2016). GNRH-Agonist or Antagonist in the Treatment of Prostate Cancer: A Comparision Based on Oncological Results. Urol. J..

[24] Engel J.B., Hahne J.C., Häusler S.F.M., Meyer S., Segerer S.E., Diessner J., Dietl J., Honig A. (2012). Peptidomimetic GnRH Antagonist AEZS-115 Inhibits the Growth of Ovarian and Endometrial Cancer Cells. Anticancer Res..

[25] He H., Deng X., Wang Z., Chen J. (2025). Recent progress in the development of peptide-drug conjugates (PDCs) for cancer therapy. Eur. J. Med. Chem..

[26] Ghaly H.S.A., Varamini P. (2021). New Drug Delivery Strategies Targeting the GnRH Receptor in Breast and Other Cancers. Endocr. Relat. Cancer.

[27] Engel J., Emons G., Pinski J., Schally A.V. (2012). AEZS-108: A Targeted Cytotoxic Analog of LHRH for the Treatment of Cancers Positive for LHRH Receptors. Expert Opin. Investig. Drugs.

[28] Miller D.S., Scambia G., Bondarenko I., Westermann A.M., Oaknin A., Oza A.M., Lisyanskaya A.S., Vergote I., Wenham R.M., Temkin S.M. (2018). ZoptEC: Phase III Randomized Controlled Study Comparing Zoptarelin with Doxorubicin as Second Line Therapy for Locally Advanced, Recurrent, or Metastatic Endometrial Cancer (NCT01767155). J. Clin. Oncol..

[29] Biniari G., Markatos C., Nteli A., Tzoupis H., Simal C., Vlamis-Gardikas A., Karageorgos V., Pirmettis I., Petrou P., Venihaki M. (2023). Rational Design, Synthesis and Binding Affinity Studies of Anthraquinone Derivatives Conjugated to Gonadotropin-Releasing Hormone (GnRH) Analogues towards Selective Immunosuppression of Hormone-Dependent Cancer. Int. J. Mol. Sci..

[30] Neidhart J.A., Gochnour D., Roach R., Hoth D., Young D. (1986). A Comparison of Mitoxantrone and Doxorubicin in Breast Cancer. J. Clin. Oncol..

[31] Markatos C., Biniari G., Chepurny O.G., Karageorgos V., Tsakalakis N., Komontachakis G., Vlata Z., Venihaki M., Holz G.G., Tselios T. (2024). Cytotoxic Activity of Novel GnRH Analogs Conjugated with Mitoxantrone in Ovarian Cancer Cells. Molecules.

[32] Markatos C., Biniari G., Karageorgos V., Chepurny O.G., Venihaki M., Holz G.G., Tselios T., Liapakis G. (2024). Two GnRH-Mitoxantrone Conjugates, Con-3 and Con-7, Target Endometrial Cancer Cells. Curr. Mol. Pharmacol..

[33] Laimou D.K., Katsara M., Matsoukas M.-T.I., Apostolopoulos V., Troganis A.N., Tselios T.V. (2010). Structural Elucidation of Leuprolide and Its Analogues in Solution: Insight into Their Bioactive Conformation. Amino Acids.

[34] Willcott M.R. (2009). MestRe Nova. J. Am. Chem. Soc..

[35] Case D.A., Babin V., Berryman J., Betz R.M., Cai Q., Cerutti D.S., Cheatham T.E., Darden T.A., Duke R.E., Gohlke H. (2014). Amber 14. https://www.researchgate.net/profile/Tai-Sung-Lee/publication/270588529_Amber_2014/links/5501add20cf2d60c0e5fd2b6/Amber-2014.pdf.

[36] Pettersen E.F., Goddard T.D., Huang C.C., Couch G.S., Greenblatt D.M., Meng E.C., Ferrin T.E. (2004). UCSF Chimera—A Visualization System for Exploratory Research and Analysis. J. Comput. Chem..

[37] Schmidt M.W., Baldridge K.K., Boatz J.A., Elbert S.T., Gordon M.S., Jensen J.H., Koseki S., Matsunaga N., Nguyen K.A., Su S. (1993). General Atomic and Molecular Electronic Structure System. J. Comput. Chem..

[38] Gordon M.S., Schmidt M.W., Dykstra C.E., Frenking G., Kim K.S., Scuseria G.E. (2005). Chapter 41—Advances in Electronic Structure Theory: GAMESS a Decade Later. Theory and Applications of Computational Chemistry.

[39] Becke A.D. (1993). Density-functional Thermochemistry. III. The Role of Exact Exchange. J. Chem. Phys..

[40] Pople J.A., Gill P.M.W., Johnson B.G. (1992). Kohn—Sham Density-Functional Theory within a Finite Basis Set. Chem. Phys. Lett..

[41] Hertwig R.H., Koch W. (1997). On the Parameterization of the Local Correlation Functional. What Is Becke-3-LYP?. Chem. Phys. Lett..

[42] Trott O., Olson A.J. (2010). AutoDock Vina: Improving the Speed and Accuracy of Docking with a New Scoring Function, Efficient Optimization, and Multithreading. J. Comput. Chem..

[43] Eberhardt J., Santos-Martins D., Tillack A.F., Forli S. (2021). AutoDock Vina 1.2.0: New Docking Methods, Expanded Force Field, and Python Bindings. J. Chem. Inf. Model..

