# Supplementary material for: NMR Structural Elucidation of Mitoxantrone–Gonadotropin-Releasing Hormone (GnRH) Conjugates Implicated in Hormone-Dependent Cancer
Source: Int J Mol Sci. 2026 Aug 20;27(16):7437. doi: 10.3390/ijms27167437 (PMC13513673; doi:10.3390/ijms27167437)
Supplement: Supplementary file 1 [file ijms-27-07437-s001.zip › SI_SI1_NMR Structural Elucidation of Mitoxantrone-Gonadotropin-Releasing Hormone (GnRH) Conjugates Implicated In Hormone-Dependent Cancer_revised.pdf]

NMR Structural Elucidation of Mitoxantrone–Gonadotropin–Releasing Hormone (GnRH) Conjugates Implicated in Hormone–Dependent Cancer

Georgia Biniari [1], Haralambos Tzoupis [1], Uroš Javornik [2], Nikitas Georgiou [3,4], Georgios Liapakis [5], Thomas Mavromoustakos [3], Theodore Tselios [1] and Carmen Simal [1,*]

[1]Department of Chemistry, University of Patras, 26504 Rion Patras, Greece

[2]Slovenian NMR Centre, National Institute of Chemistry, SI-1001 Ljubljana, Slovenia

[3]Department of Chemistry, National and Kapodistrian University of Athens, 10679, Athens, Greece

[4]Center for Interdisciplinary Biosciences, Technology and Innovation Park, P.J. Safarik University in Kosice, Jesenna 5, 04001 Kosice, Slovakia

[5]Department of Pharmacology, School of Medicine, University of Crete, 71003 Heraklion, Greece

Table of Contents

1. Materials and methods used in experiments with D_2O S2
2. Methods used in experiments with DMSO-d_6 .... S3
3. NMR assignments of the Peptides S4
4. NMR assignments of the Conjugates S8
5. Structural elucidation S10
6. Molecular Modeling S10
7. Molecular Docking Studies S17

1. Materials and methods used in experiments with D_2O

D_2O (99.9 atom% D) was purchased from Eurisotop. High precision 5 mm NMR tubes from Deutero GmbH were used. The high-resolution NMR spectra were recorded at 298K on a Bruker Avance Neo 800 MHz equipped with a cryogenically cooled 5mm $^{1}H/^{13}C/^{15}N/D$ Z-gradient probe. All data were collected in phase-sensitive mode using pulse sequences and phase-cycling routines provided in Bruker libraries of pulse programs. 1D ^{1}H NMR spectra were acquired using a single-pulse experiment with a 90° excitation pulse (pulse program *zg*) with a spectral width (SW) of 14705.9 Hz, 32768 data points, an acquisition time of 2.228 s, a relaxation delay of 5.0 s, and 16 scans. 1D ^{13}C NMR spectra were acquired using a pulse sequence with pulsed-field gradients and a 30° flip angle (pulse program *zgpg30*) with broadband ^{1}H decoupling, a SW of 41666.7 Hz, 32768 data points, an acquisition time of 0.786 s, a relaxation delay of 1.0 s, and 7072 scans. The 2D homonuclear COSY spectrum was recorded using a gradient-selected, phase-sensitive double-quantum filtered ^{1}H–^{1}H correlation pulse sequence (pulse program *cosygpmfppqf*), a SW of 14706 Hz in both dimensions, an acquisition time of 0.139 s in F_2, 2048 data points in F_2 and 256 in F_1, 16 scans, a relaxation delay of 1.0 s, and an acquisition time of 0.139 s in F_2. The 2D homonuclear TOCSY spectrum was recorded using a gradient-selected, phase-sensitive pulse sequence with DIPSI-2 isotropic mixing and excitation sculpting for solvent suppression (pulse program *dipsi2esfbgpph*), a SW of 14706 Hz in both dimensions, an acquisition time of 0.139 s in F_2, 2048 data points in t_2 and 256 in t_1, an acquisition time of 0.139 s in F_2, 16 scans, a relaxation delay of 1.0 s, and a mixing time of 80 ms. The 2D homonuclear NOESY spectrum was acquired using a gradient-selected, phase-sensitive pulse sequence with excitation sculpting for water suppression (pulse program *noesyesgpph*), a SW of 14706 Hz in both dimensions, an acquisition time of 0.139 s in F_2, 4096 in t_2 and 1024 in t_1 data points, 16 scans, a relaxation delay of 1.0 s, and a mixing time of 150 ms. The 2D heteronuclear HMBC spectrum was recorded using a gradient-selected, phase-sensitive long-range ^{1}H-^{13}C correlation pulse sequence (pulse program *hmbcetgpl3nd*), a SW of 14706 Hz in F_2 (^{1}H) and 46261 Hz in F_1 (^{13}C), 4096 data points in F_2 and 1024 in F_1, an acquisition time of 0.139 s in F_2, 64 scans and a relaxation delay of 1.0 s. The 2D heteronuclear HSQC spectrum was recorded using a gradient-selected, phase-sensitive heteronuclear ^{1}H-^{13}C correlation pulse sequence with echo-detection (pulse program *hsqcedetgpsp.3*), a SW of 14706 Hz in F_2 (^{1}H) and 40226 Hz in F_1 (^{13}C), 4096 complex points in F_2 and 1024 in F_1, 32 scans, a relaxation

delay of 1.0 s, and an acquisition time of 0.1 s in F_2. Raw NMR data were processed with MestReNova software (Version: 14.3.3-33362) [1].

2. Methods used in experiments with DMSO-d_6

1D ^{1}H NMR spectra were acquired using a single-pulse experiment with a 30° excitation pulse (pulse program zg30) with a spectral width (SW) of 20833.3 Hz, 32768 data points, an acquisition time of 1.57 s, a relaxation delay of 3.0 s, and 64 scans. 1D ^{13}C NMR spectra were acquired using a pulse sequence with pulsed-field gradients and a 30° flip angle (pulse program *zgpg30*) with broadband ^{1}H decoupling, a SW of 48543.7 Hz, 32768 data points, an acquisition time of 0.675 s, a relaxation delay of 2.0 s, and 4000 scans. The 2D homonuclear TOCSY spectrum was recorded using a gradient-selected, phase-sensitive pulse sequence with DIPSI-2 isotropic mixing (pulse program *dipsi2gpphzs*), a SW of 14706 Hz in both dimensions, an acquisition time of 0.139 s in F_2, 2048 data points in t_2 and 400 in t_1, 8 scans, a relaxation delay of 1.5 s, and a mixing time of 80 ms. The 2D homonuclear NOESY spectrum was acquired using a gradient-selected, phase-sensitive pulse sequence with zero-quantum suppression (pulse program *noesygpphzs*), a SW of 14706 Hz in F_2 and 14397 Hz in F_1, an acquisition time of 0.139 s in F_2, 2048 data points in t_2 and 400 in t_1 data points, 16 scans, a relaxation delay of 2.0 s, and a mixing time of 150 ms. The 2D heteronuclear HMBC spectrum was recorded using a gradient-selected, phase-sensitive long-range ^{1}H-^{13}C correlation pulse sequence (pulse program *hmbcetgpl3nd*), a SW of 14706 Hz in F_2 (^{1}H) and 44249 Hz in F_1 (^{13}C), an acquisition time of 0.139 s in F_2, 2048 data points in F_2 and 128 in F_1, 32 scans, a relaxation delay of 1.5 s, and an acquisition time of 0.139 s in F_2. The 2D heteronuclear HSQC spectrum was recorded using a gradient-selected, phase-sensitive heteronuclear ^{1}H-^{15}N correlation pulse sequence (pulse program *hsqcetf3gp*), a SW of 14706 Hz in F_2 (^{1}H) and 17832 Hz in F_1 (^{15}N), an acquisition time of 0.139 s in F2, 2048 complex points in F2 and 400 in F1, 32 scans, a relaxation delay of 1.0 s, and an acquisition time of 0.139 s in F_2.

3. NMR assignments of the Peptides

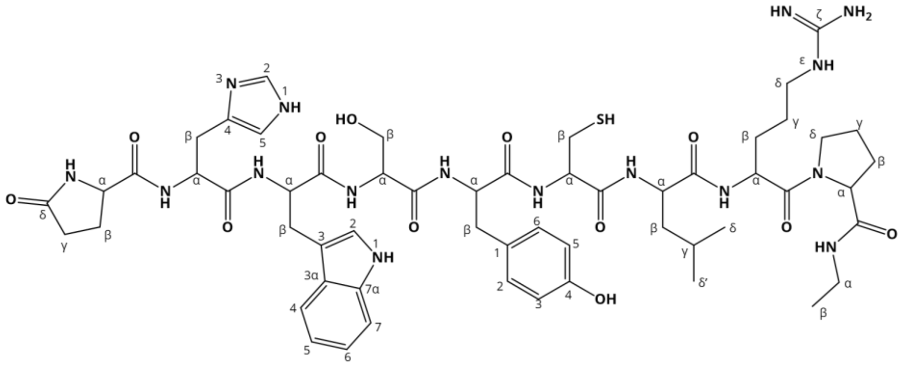

pGlu¹ - His² - Trp³ – Ser⁴-Tyr⁵-DCys⁶-Leu⁷-Arg⁸-Pro⁹-NHEt¹⁰

Figure S1: Structure of the peptide component of con3, with atom numbering corresponding to the NMR assignments.

The peptide moiety of conjugate con3 (Figure S1) corresponds to analogue 2, whose synthesis was previously described in our earlier work [2]. Its structural identification in D_2O (Table S1) has been accomplished using a combination of two-dimensional NMR experiments, including ^{1}H-^{1}H DQF-COSY, ^{1}H-^{1}H TOCSY, ^{1}H-^{1}H NOESY, ^{1}H-^{13}C HSQC, and ^{1}H-^{13}C HMBC.

Table S1: ^{1}H and ^{13}C (*italics*) chemical shift assignments of the peptide component of con3 (800 MHz, D_2O, 298 K). Chemical shifts are referenced to D_2O ($\delta^{1}H$ = 4.80).

	Residue chemical shift			
	NH	αH	βH	Others
	CO	*αC*	*βC*	
**pGlu¹**	-	4.10	1.55, 2.27	γH: 2.19, 2.25
	174.49	*56.51*	*25.04*	*γC: 29.12; δC: 182.07*
**His²**	-	4.57	2.98, 3.07	2H: 8.47; 5H: 7.09
	171.06	*52.26*	*26.14*	*2C: 133.53; 5C: 117.10; 4C: 128.27*
**Trp³**	-	4.64	3.07, 3.12	2H: 7.11; 4H: 7.52; 5H: 7.04; 6H: 7.16; 7H: 7.41
	173.35	*54.34*	*27.23*	*2C: 124.42; 3C: 108.66; 4C: 118.21; 5C: 119.35; 6C: 121.94; 7C: 111.83; 3αC: 126.93; 9αC: 136.12*
**Ser⁴**	-	4.24	3.66	-
	170.86	*55.53*	*61.04*	-
**Tyr⁵**	-	4.36	2.90, 2.86	2,6H: 7.02; 3,5H: 6.74
	172.98	*55.76*	*35.81*	*1C: 127.57; 2,6C: 130.62; 3,5C: 115.62; 4C: 154.70*
**DCys⁶**	-	4.38	2.53, 2.71	-
	171.42	*55.22*	*25.20*	-
**Leu⁷**	-	4.30	1.49, 1.58	γH: 1.50; δH: 0.77; δ΄H: 0.83
	174.09	*52.39*	*39.68*	*γC: 24.27; δC: 20.59; δ΄C: 22.16*
**Arg⁸**	-	4.40	1.54, 1.68	γH: 1.45; δH:3.00
	171.13	*50.96*	*27.57*	*γC: 23.98; δC: 40.51; ζC: 156.57*
**Pro⁹**	-	4.11	1.73, 2.07	γH: 1.78, 1.88; δH: 3.40, 3.53
	173.53	*60.81*	*29.39*	*γC: 24.50; δC: 47.85*
**NHEt¹⁰**	-	3.06, 3.10	0.99	-
	-	*34.41*	*13.44*	-

Table S2: ^{1}H and ^{13}C (*italics*) chemical shift assignments of the peptide component of con3 (800 MHz, DMSO-d_6, 298 K). Chemical shifts are referenced to DMSO-d_6 (δ^{1}H = 2.50).

	Residue chemical shift			
	NH	αH	βH	Others
	CO	*αC*	*βC*	
**pGlu[1]**	7.69	3.98	2.16	γH: 1.71, 2.06
	172.96	*55.90*	*25.46*	*γC: 29.56; δC: 177.94*
**His[2]**	8.13	4.60	2.90, 3.03	2H: 8.88; 5H: 7.26
	170.11	*51.78*	*24.64*	*2C: 134.31; 4C: 129.82; 5C: 117.42*
**Trp[3]**	8.06	4.65	2.99, 3.16	1H: 10.80; 2H: 7.13; 4H: 7.62; 5H: 6.92; 6H: 7.04; 7H: 7.32
	172.08	*53.76*	*28.21*	*2C: 124.17; 3C: 110.15; 3αC: 127.79; 4C: 118.69; 5C: 119.04; 6C: 121.34; 7C: 111.70; 7αC: 136.52*
**Ser[4]**	8.31	4.36	3.53, 3.60	-
	170.31	*55.45*	*62.24*	-
**Tyr[5]**	8.03	4.52	2.75, 2.95	2,6H: 7.06; 3,5H: 6.64
	171.44	*55.06*	*37.30*	*1C: 127.99; 2,6C: 115.41; 3,5C: 130.66; 4C: 156.34*
**DCys[6]**	8.18	4.45	2.03, 2.68	-
	169.58	*55.25*	*27.05*	-
**Leu[7]**	8.16	4.34	1.41, 1.63	γH: 1.47; δH: 0.87; δ′H: 0.83
	172.23	*51.32*	*41.30*	*γC: 24.58; δC: 23.70; δ′C: 21.76*
**Arg[8]**	8.15	4.47	1.72	γH: 1.54; δH: 3.09; εH: 7.46
	169.96	*50.57*	*28.55*	*γC: 24.99; δC: 41.09; ζC: 157.12*
**Pro[9]**	-	4.22	1.80, 1.91	γH: 1.71, 2.01; δH: 3.51, 3.66
	171.67	*59.99*	*24.99*	*γC: 29.84; δC: 47.28*
**NHEt[10]**	7.83	3.06	0.99	-
	-	*33.81*	*15.12*	-

The sequential resonance assignment in D_2O was initiated from the singlet observed at 8.47 ppm, corresponding to the 2H proton of the histidine aromatic ring. The remaining residues in the peptide sequence were subsequently assigned through analysis of the scalar and through-space connectivities obtained from the complementary NMR spectra. A similar approach was employed for the assignment in DMSO-d_6 (Table S2). The sequential assignment was initiated from the singlet at 10.80 ppm, corresponding to the indole NH proton of the tryptophan residue. Amino acid spin patterns were identified through analysis of the scalar and through-space connectivities, which ultimately led to the complete sequence assignment of the peptide.

The peptide moiety of conjugate con7 (Figure S2) corresponds to analogue 4, whose synthesis was previously described in our earlier work [2]. The sequential resonance assignments in D_2O (Table S3) and DMSO-*d6* (Table S4) have been carried out in a similar manner to those performed for the peptide component of con3.

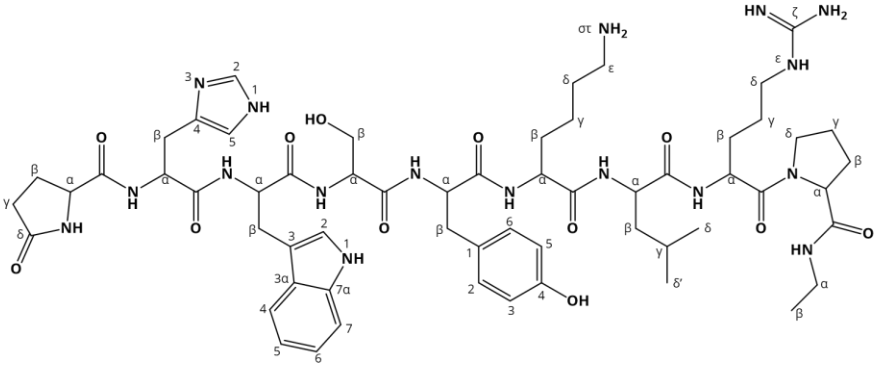

pGlu¹ - His² - Trp³ – Ser⁴-Tyr⁵-DLys⁶-Leu⁷-Arg⁸-Pro⁹-NHEt¹⁰

Figure S2: Structure of the peptide component of con7, with atom numbering corresponding to the NMR assignments.

Table S3: ^{1}H and ^{13}C (*italics*) chemical shift assignments of the peptide component of con7 (800 MHz, D_2O, 298 K). Chemical shifts are referenced to D_2O (δ^{1}H = 4.80).

	Residue chemical shift			
	NH	αH	βH	Others
	CO	*αC*	*βC*	
**pGlu¹**	-	4.10	1.54, 2.27	γH: 2.24, 2.18
	174.54	*56.50*	*25.04*	*γC: 29.14; δC: 182.06*
**His²**	-	4.57	2.98, 3.08	2H: 8.48, 5H: 7.10
	171.11	*52.28*	*26.08*	*2C: 133.51; 5C: 117.11; 4C: 128.24*
**Trp³**	-	4.64	3.10, 3.15	2H: 7.13; 4H: 7.52; 5H: 7.05; 6H: 7.16; 7H: 7.41
	173.33	*54.42*	*27.20*	*2C: 124.43; 3C: 108.65; 4C: 118.19; 5C: 119.37; 6C: 121.96; 7C: 111.87; 7αC: 136.98; 3αC: 126.92*
**Ser⁴**	-	4.24	3.63, 3.66	-
	170.77	*55.45*	*61.09*	-
**Tyr⁵**	-	4.31	2.80, 2.90	2,6H: 7.02; 3,5H: 6.75
	173.05	*55.86*	*36.00*	*1C: 127.49; 2,6C: 130.59; 3,5C: 115.53; 4C: 154.60*
**DLys⁶**	-	4.04	1.41, 1.57	γH: 0.88; δH: 1.45; εH: 2.83
	173.46	*53.46*	*30.31*	*γC: 21.93; δC: 26.26; εC: 39.07*
**Leu⁷**	-	4.27	1.48, 1.55	γH: 1.44; δH: 0.76; δ΄H: 0.82
	174.14	*52.17*	*39.69*	*γC: 24.53; δC: 20.61; δ΄C: 22.16*
**Arg⁸**	-	4.37	1.55, 1.68	γH: 1.45; δH: 3.01
	171.26	*51.02*	*27.53*	*γC: 23.96; δC: 40.54; ζC: 156.61*
**Pro⁹**	-	4.11	1.74, 2.08	γH: 1.78, 1.89; δH: 3.40, 3.55
	173.52	*60.81*	*29.40*	*γC: 24.29; δC: 47.88*
**NHEt¹⁰**	-	3.05, 3.10	0.98	-
	-	*34.41*	*13.45*	-

Table S4: ^{1}H and ^{13}C (*italics*) chemical shift assignments of the peptide component of con7 (800 MHz, DMSO-d_6, 298 K). Chemical shifts are referenced to DMSO-d_6 (δ^1H = 2.50).

	Residue chemical shift			
	NH / *CO*	αH / *αC*	βH / *βC*	Others
**pGlu[1]**	7.69<br>*173.01*	3.97<br>*55.89*	1.72, 2.15<br>*25.46*	γH: 2.04, 2.07<br>*γC: 29.56; δC: 177.93*
**His[2]**	8.16<br>*170.08*	4.60<br>*51.80*	2.90, 3.04<br>*27.50*	2H: 8.94; 5H: 7.27<br>*2C: 134.27; 5C: 117.42; 4C: 129.62*
**Trp[3]**	8.03<br>*172.06*	4.65<br>*53.71*	2.99, 3.15<br>*28.25*	1H: 10.81; 2H: 7.13; 4H: 7.62; 5H: 6.92; 6H: 7.05; 7H: 7.32<br>*2C: 124.21; 3C: 110.13; 3αC: 127.79; 4C: 118.68; 5C: 118.40; 6C: 121.33; 7C: 111.71; 7αC: 136.53*
**Ser[4]**	8.31<br>*170.25*	4.36<br>*55.41*	3.52, 3.60<br>*62.25*	-<br>-
**Tyr[5]**	8.02<br>*171.29*	4.50<br>*55.06*	2.73, 2.91<br>*37.40*	2,6H: 7.04; 3,5H: 6.65<br>*1C: 128.22; 2,6C: 130.56; 3,5C: 115.36; 4C: 156.31*
**DLys[6]**	8.08<br>*171.52*	4.27<br>*52.66*	1.56<br>*32.25*	γH: 1.12; δH: 1.47; εH: 2.70; στH: 7.70<br>*γC: 22.46; δC: 27.06; εC: 39.15*
**Leu[7]**	8.09<br>*172.34*	4.33<br>*51.08*	1.46<br>*41.27*	γH: 1.55; δH: 0.82; δ´H: 0.87<br>*γC: 32.24; δC: 21.70; δ´C: 23.68*
**Arg[8]**	8.14<br>*170.05*	4.46<br>*50.61*	1.73<br>*28.52*	γH: 1.55; δH: 3.09; εH: 7.57<br>*γC: 25.00; δC: 41.08; ζC: 157.21*
**Pro[9]**	-<br>*171.66*	4.21<br>*60.02*	1.74, 2.01<br>*29.83*	γH: 1.80, 1.91; δH: 3.52, 3.67<br>*γC: 25.01; δC: 47.29*
**NHEt[10]**	7.82<br>-	3.01, 3.07<br>*33.81*	0.99<br>*15.11*	-<br>-

4. NMR assignments of the Conjugates

Table S5: ^{1}H and ^{13}C (*italics*) chemical shift assignments of con3 (800 MHz, D_2O, 298 K). Chemical shifts are referenced to D_2O (δ^{1}H = 4.80).

	Residue chemical shift			
	NH<br>*CO*	αH<br>*αC*	βH<br>*βC*	Others
**pGlu[1]**	-	4.06	2.13, 2.22	γH: 1.46, 2.22
	174.44	*56.44*	*29.10*	*γC: 25.02; δC: 181.96*
**His[2]**	-	4.52	2.92, 3.01	2H: 8.43; 5H: 7.03
	171.07	*52.20*	*25.99*	*2C: 133.50; 5C: 117.01; 4C: 128.28*
**Trp[3]**	-	4.57	2.94, 2.99	2H: 6.98; 4H: 7.34; 5H: 6.89; 6H: 7.02; 7H: 7.27
	173.14	*54.17*	*27.29*	*2C: 124.24; 3C: 108.58; 3αC: 126.82; 4C: 118.05; 5C: 119.17; 6C: 121.79; 7C: 111.67; 7αC: 135.99*
**Ser[4]**	-	4.23	3.58, 3.61	-
	170.64	*55.30*	*61.12*	-
**Tyr[5]**	-	4.25	2.55, 2.64	2,6H: 6.66; 3,5H: 6.43
	172.45	*55.16*	*36.07*	*1C: 127.20; 2,6C: 130.21; 3,5C: 115.11; 4C: 154.46*
**DCys[6]**	-	4.40	2.65, 2.77	-
	171.23	*52.41*	*38.45*	-
**Leu[7]**	-	4.24	1.43, 1.50	γH: 1.43; δH: 0.71; δ'H: 0.76
	173.91	*52.30*	*39.77*	*γC: 24.28; δC: 20.67; δ'C: 22.14*
**Arg[8]**	-	4.36	1.52, 1.66	γH: 1.43; δH: 2.98
	171.15	*50.98*	*27.55*	*γC: 23.95; δC: 40.54; ζC: 156.58*
**Pro[9]**	-	4.12	1.74, 2.07	γH: 1.78, 1.89; δH: 3.41, 3.54
	173.49	*60.80*	*29.38*	*γC: 24.53; δC: 47.86*
**NHEt[10]**	-	3.04, 3.08	0.97	-
	-	*34.41*	*13.46*	-
	Mitoxantrone Analogue			
	**1, 4**			-
				153.82
	**2, 3**			6.77
				124.55
	**5, 8**			-
				145.74
	**6, 7**			6.91
				123.83
	**4α, 9α**			-
				114.57
	**8α, 10α**			-
				117.10
	**11, 11'**			3.63
				38.63
	**12, 12'**			3.32
				46.57
	**13**			3.27, 3.42
				46.30
	**13'**			3.21
				49.30
	**14**			4.36
				60.15
	**14'**			3.82
				56.56
	**15**			-
				173.37
	**16**			2.75
				31.94
	**17**			2.68
				33.35

The sequential resonance assignment of the conjugates (Table S5, S6) in D_2O was initiated from the identification of the peptide structure, following the same procedure used previously for the peptides. Therefore, the mitoxantrone analogue

was assigned through analysis of the long-range correlations observed between its protons and the peptide atoms in the NMR spectra.

Table S6: ^{1}H and ^{13}C (*italics*) chemical shift assignments of con7 (800 MHz, D_2O, 298 K). Chemical shifts are referenced to D_2O ($\delta^{1}H = 4.80$).

Residue chemical shift				
	NH<br>*CO*	αH<br>*αC*	βH<br>*βC*	Others
**pGlu^{1}**	-<br>*174.48*	4.08<br>*56.46*	2.15, 2.24<br>*29.12*	γH: 1.38, 1.50<br>*γC: 25.00; δC: 181.97*
**His^{2}**	-<br>*171.09*	4.55<br>*52.22*	2.96, 3.05<br>*26.02*	2H: 8.46; 5H: 7.07<br>*2C: 133.50; 5C: 117.08; 4C: 128.25*
**Trp^{3}**	-<br>*173.11*	4.58<br>*54.33*	3.00, 3.05<br>*27.21*	2H: 7.01; 4H: 7.37; 5H: 6.91; 6H: 7.03; 7H: 7.28<br>*2C: 124.26; 3C: 108.57; 3αC: 126.85; 4C: 118.04; 5C: 119.20; 6C: 121.78; 7C: 111.68; 7αC: 136.01*
**Ser^{4}**	-<br>*170.61*	4.24<br>*55.45*	3.59, 3.63<br>*61.16*	-<br>-
**Tyr^{5}**	-<br>*172.68*	4.26<br>*55.65*	2.70, 2.81<br>*36.16*	2,6H: 6.86; 3,5H: 6.61<br>*1C: 127.38; 2,6C: 130.37; 3,5C: 115.36; 4C: 154.63*
**DLys^{6}**	-<br>*173.57*	3.99<br>*53.46*	1.37, 1.50<br>*30.60*	γH: 0.83; 0.89; δH: 1.25; εH: 2.93, 2.97<br>*γC: 22.26; δC: 27.83; εC: 38.86*
**Leu^{7}**	-<br>*174.10*	4.23<br>*52.15*	1.45, 1.52<br>*39.65*	γH: 1.43; δH: 0.73; δ′H: 0.80<br>*γC: 24.30; δC: 20.61; δ′C: 22.19*
**Arg^{8}**	-<br>*171.19*	4.39<br>*50.97*	1.55, 1.69<br>*27.55*	γH: 1.46; δH: 3.01<br>*γC: 23.97; δC: 40.53; ζC: 156.60*
**Pro^{9}**	-<br>*173.49*	4.13<br>*60.80*	1.75, 2.09<br>*29.41*	γH: 1.79, 1.90; δH: 3.42, 3.57<br>*γC: 24.55; δC: 47.87*
**NHEt^{10}**	-<br>-	3.05, 3.09<br>*34.41*	0.98<br>*13.46*	-<br>-
**Ahx Linker**	-<br>*176.34*	2.03, 2.09<br>*35.66*	1.36<br>*27.85*	γH: 1.08, 1.17; δH: 1.39,1.46; εH: 2.91, 3.05; ηH: 2.34, 2.50; θH: 2.73, 2.81<br>*γC: 25.53; δC: 25.03; εC: 39.14; ζC: 173.13; ηC: 34.86; θC: 33.19*

Mitoxantrone Analogue	
**1, 4**	-<br>*153.90*
**2, 3**	6.84<br>*124.63*
**5, 8**	-<br>*145.79*
**6, 7**	6.98<br>*123.94*
**4α, 9α**	-<br>*153.89*
**8α, 10α**	-<br>*145.70, 145.83*
**11, 11’**	3.67<br>*38.49*
**12**	3.33<br>*46.67*
**12’**	3.30<br>*46.36*
**13**	3.43<br>*46.23*
**13’**	3.21<br>*49.49*
**14**	4.38<br>*60.89*
**14’**	3.81<br>*56.56*
**15**	-<br>*171.09*
**16**	3.46<br>*39.97*

5. Structural elucidation

Table S7: Observed NOE connectivities for the peptide component of con3 and con7 based on NOESY spectra.

Correlations							
con3's peptide						con7's peptide	
αH_{P9}	βH_{NHEt10}	γH_{R8}	NH_{NHEt10}	$5H_{W3}$	αH_{Y5}	αH_{R8}	δH_{L7}, $\delta' H_{L7}$
αH_{pGlu1}	$5H_{H2}$	γH_{R8}	αH_{P9}	$6H_{W3}$	αH_{Y5}	αH_{DK6}	δH_{L7}, $\delta' H_{L7}$
αH_{P9}	δH_{L7}, $\delta' H_{L7}$	γH_{R8}	δH_{P9}, $\delta' H_{P9}$	$3,5H_{Y5}$	αH_{W3}	αH_{P9}	δH_{L7}, $\delta' H_{L7}$
εH_{R8}	δH_{L7}, $\delta' H_{L7}$	γH_{R8}	εH_{R8}			δH_{L7}, $\delta' H_{L7}$	δH_{P9}, $\delta' H_{P9}$
αH_{w3}	δH_{L7}, $\delta' H_{L7}$	γH_{R8}	βH_{S4}			NH_{S4}	δH_{L7}, $\delta' H_{L7}$
αH_{Y5}	δH_{L7}, $\delta' H_{L7}$	γH_{R8}	αH_{Y5}			βH_{S4}	δH_{L7}, $\delta' H_{L7}$
$2H_{W3}$	δH_{L7}, $\delta' H_{L7}$	γH_{R8}	$2H_{W3}$			αH_{Y5}	δH_{L7}, $\delta' H_{L7}$
$5H_{W3}$	δH_{L7}, $\delta' H_{L7}$	γH_{R8}	$5H_{W3}$			$2H_{W3}$	δH_{L7}, $\delta' H_{L7}$
$3,5H_{Y5}$	δH_{L7}, $\delta' H_{L7}$	γH_{R8}	$3,5H_{Y5}$			$5H_{W3}$	δH_{L7}, $\delta' H_{L7}$
αH_{pGlu1}	αH_{H2}	NH_{S4}	$2H_{W3}$			αH_{L7}	γH_{DK6}
αH_{pGlu1}	$5H_{H2}$	αH_{Y5}	$6H_{W3}$			NH_{S4}	γH_{DK6}
αH_{pGlu1}	NH_{W3}	$2H_{W3}$	αH_{H2}			βH_{S4}	γH_{DK6}
αH_{pGlu1}	$2H_{W3}$	$5H_{W3}$	αH_{H2}			αH_{S4}	γH_{DK6}
αH_{pGlu1}	NH_{Y5}	$5H_{W3}$	NH_{S4}			$3,5H_{Y5}$	γH_{DK6}

6. Molecular Modeling

Geometry optimization. All structures were optimized with the GAMESS software [1,2], employing the density functional theory (DFT) [3,4] and the Hartree - Fock (HF) approximation. Additionally, the B3LYP/6-311G atomic basis set was used, and the convergence criterion was set at 0.0001 [4,5].

Molecular Dynamics (MD) simulations. The ff14SB and GAFF (General Amber Force Fields) force fields were employed to describe the parameters for the peptide core and for the organic molecules, respectively. All analogues were dissolved in water, using the TIP3P water model, and DMSO to construct the separate systems (4 in water and 4 in DMSO), creating a total of 8 systems. For the solvation, a truncated octahedral box with a 10Å cut-off distance was used. The overall charge of the analogues was neutralized by the addition of Cl^- ions. Initially, the energy minimization of the systems was carried out in two steps. In the first step, a force constant was used in the peptide backbone (10 kcal mol^{-1} $Å^{-2}$) for constraining the atom movement, while in the second step, the constraints on the solute atoms were removed. The minimization for the first 5000 steps of each stage was performed using the "steepest descent" method, while in the subsequent steps of each stage, the minimization was performed using the

"conjugate gradient" method. The systems were then gradually heated from 0 K to 300 K using the Langevin thermostat with a time step of 0.002 ps for 0.5 ns, followed by 0.5 ns of pressure equilibration at 1 atm with a time step of 0.002 ps. In both heating and pressure equilibration, a force constant of (10 kcal mol^{-1} $Å^{-2}$) was applied to the solute. The process was followed by a 1 ns unrestrained equilibration at 300 K in the NPT ensemble. The MD production phase was performed for 150 ns under constant pressure and temperature conditions. Throughout the simulation, the temperature was kept stable using the Langevin thermostat, and the SHAKE algorithm was employed to constrain the motion of bonds involving hydrogen.

MD analysis. The trajectories from the MD simulations were analyzed using the cpptraj module of AMBER. The clustering analysis was based on the hierarchical approach with an RMSD cutoff of 4.0 Å. The RMSD values for all analogues were calculated for the peptide backbone atoms in reference to the initial conformation of every analogue.

NMR Refinement. A simulated annealing protocol (50 ps) was carried out for the different peptide analogues. The protocol implemented as NMR restraints the distances derived from the NMR spectra (Tables S91-S12). The simulated annealing was performed over 50000 steps:

(1) gradually rising the temperature from 10 to 1200 K, over 5000 steps

(2) keeping the temperature at 1200 K, for 10000 steps

(3) gradually re-cooling the system for the last 35000 steps

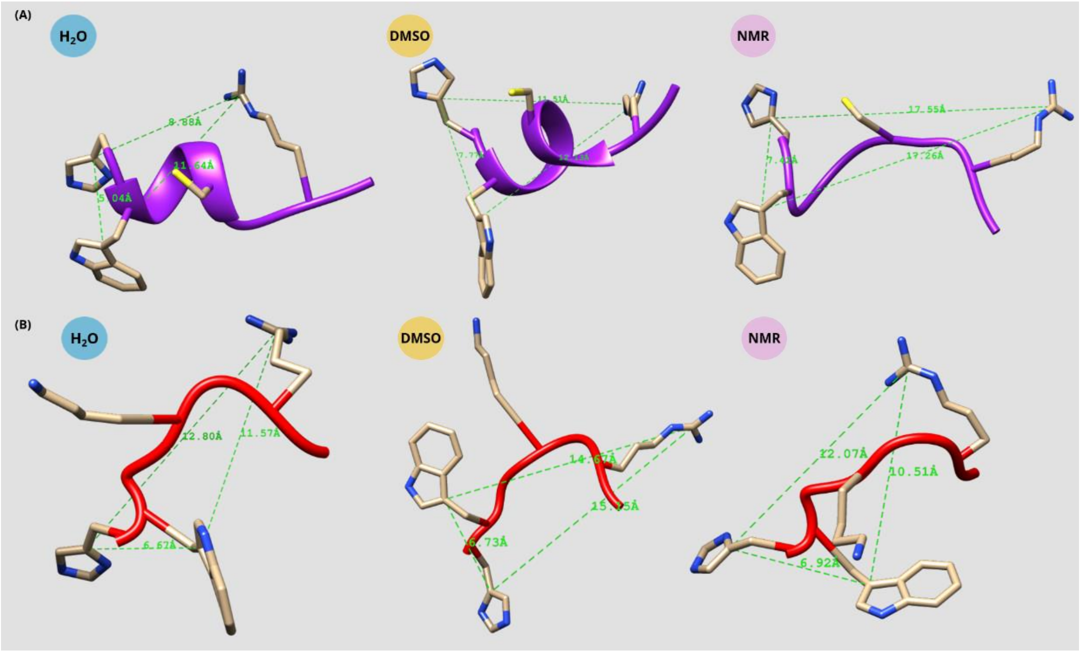

Figure S3: Conformations of the: (A) con3 and (B) con7 peptides from MD simulations in H_2O, DMSO, and NMR-refined MD simulations in DMSO. Green dashed lines indicate the triangles formed by the C_γ atom of His^2 and Trp^3 and the C_ζ of Arg^8, an arrangement considered characteristic of the putative bioactive conformation of the peptide. Only selected side chains involved in the bioactive conformation are displayed for clarity. Atoms are colored as follows: sulfur (S) in yellow, oxygen (O) in red, and nitrogen (N) in dark blue.

Table S8: Geometric triangles formed by C_γ of His^2 and Trp^3 and C_ζ of Arg^8 for the putative bioactive conformation of con3 and con7, compared to Leuprolide [3].

Distances	MD	con3	con7	Leuprolide
**d (His^2–Trp^3)**	H_2O	7.00 Å	6.84 Å	6.21 Å
	DMSO	6.85 Å	7.03 Å	7,80 Å
	NMR-refined	6.88 Å	7.00 Å	-
**d (His^2–Arg^8)**	H_2O	7.89 Å	6.26 Å	9.43 Å
	DMSO	7.15 Å	10.17 Å	9.89 Å
	NMR-refined	9.61 Å	21.87 Å	-
**d (Trp^3–Arg^8)**	H_2O	10.65 Å	9.42 Å	12.03 Å
	DMSO	10.09 Å	11.48 Å	12.76 Å
	NMR-refined	4.78 Å	16.63 Å	-

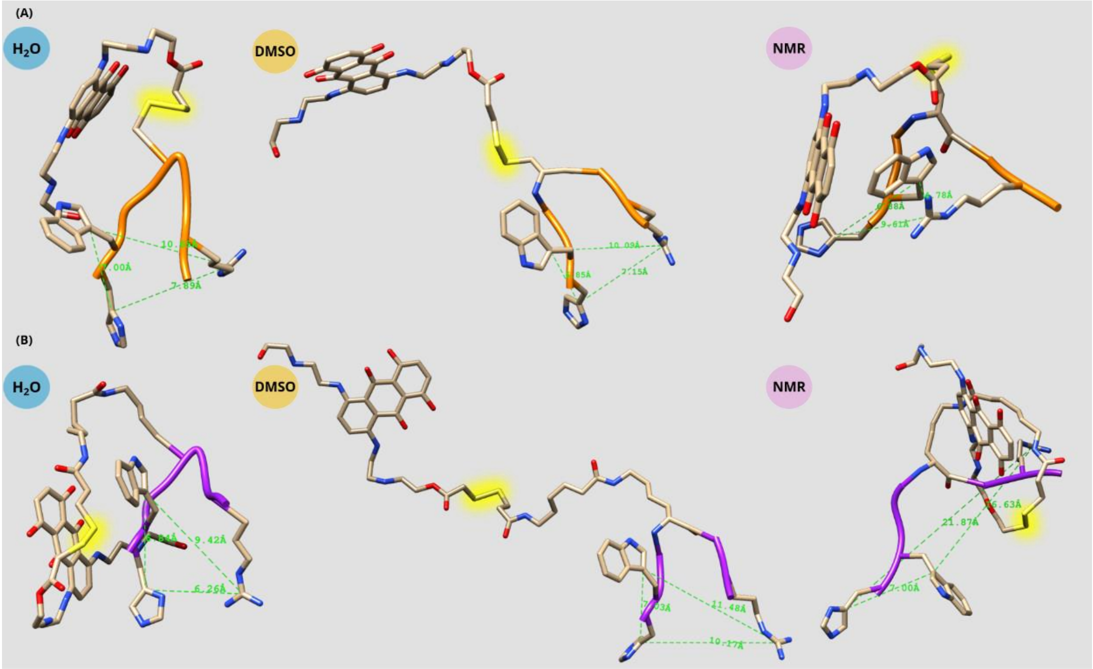

Figure S4: Conformations of con3 (A) and con7 (B) from MD simulations in H_2O, DMSO and NMR-refined MD simulations in DMSO. Green dashed lines indicate the triangles formed by C_γ of His[2] and Trp[3] and C_ζ of Arg[8], an arrangement considered characteristic of the putative bioactive conformation of the peptide. Only selected side chains involved in the bioactive conformation are displayed for clarity. Atoms are colored as follows: sulfur (S) in yellow, oxygen (O) in red, and nitrogen (N) in dark blue.

Table S9: NOE constraints employed in the NMR refinement protocol for the peptide moiety of con7.

#1st_ResID 1st_ResName 1st_H 2nd_ResID 2nd_ResName 2nd_H upper_dist_limit

7 LEU MD1 8 ARG HA 5.5

7 LEU MD1 6 LYS HA 5.5

7 LEU MD1 9 PRO HA 5.5

7 LEU MD1 9 PRO HD2 5.5

7 LEU MD1 9 PRO HD3 5.5

7 LEU MD1 4 SER H 5.5

7 LEU MD1 4 SER HB2 5.5

7 LEU MD1 4 SER HB3 5.5

7 LEU MD1 5 TYR HA 5.5

7 LEU MD1 3 TRP HD1 5.5

7 LEU MD1 3 TRP HE3 5.5

6 LYS HG3 7 LEU HA 5.5

6 LYS HG3 4 SER H 5.5

6 LYS HG3 4 SER HB2 5.5

6 LYS HG3 4 SER HB3 5.5

6 LYS HG3 5 TYR HA 5.5

6 LYS HG3 5 TYR HE1 5.5

Table S10: NOE constraints employed in the NMR refinement protocol for the peptide moiety of con3.

#1st_ResID 1st_ResName 1st_H 2nd_ResID 2nd_ResName 2nd_H upper_dist_limit

10 ET2 HB2 9 PRO HA 5.5

2 HIE HD1 1 pGL HA 5.5

7 LEU HD1 9 PRO HA 5.5

7 LEU HD1 8 ARG HE 5.5

7 LEU HD1 4 SER H 5.5

7 LEU HD1 3 TRP HA 5.5

7 LEU HD1 5 TYR HA 5.5

7 LEU HD1 3 TRP HD1 5.5

7 LEU HD1 3 TRP HE3 5.5

7 LEU HD1 5 TYR HE1 5.5

1 pGL HA 2 HIE HA 5.5

1 pGL HA 2 HIE HD1 5.5

1 pGL HA 3 TRP H 5.5

1 pGL HA 3 TRP HD1 5.5

1 pGL HA 5 TYR H 5.5

8 ARG HG2 9 PRO HA 5.5

8 ARG HG2 9 PRO HD2 5.5

8 ARG HG2 9 PRO HD3 5.5

8 ARG HG2 8 ARG HE 5.5

8 ARG HG2 4 SER HB3 5.5

8 ARG HG2 5 TYR HA 5.5

8 ARG HG2 3 TRP HD1 5.5

8 ARG HG2 3 TRP HE3 5.5

8 ARG HG2 5 TYR HE1 5.5

4 SER H 3 TRP HD1 5.5

5 TYR HA 3 TRP HZ3 5.5

3 TRP HD1 2 HIE HA 5.5

3 TRP HE3 2 HIE HA 5.5

3 TRP HE3 4 SER H 5.5

3 TRP HE3 5 TYR HA 5.5

3 TRP HZ3 5 TYR HA 5.5

5 TYR HE1 3 TRP HA 5.5

Table S11: NOE constraints employed in the NMR refinement protocol for the con7.

#1st_ResID 1st_ResName 1st_H 2nd_ResID 2nd_ResName 2nd_H upper_dist_limit

10 ET HB3 9 PRO HA 5.5

10 ET HB3 3 TRP H 5.5

7 LEU HD1 6 CYS H 5.5

7 LEU HD1 6 CYS HA 5.5

7 LEU HD1 9 PRO HA 5.5

7 LEU HD1 8 ARG H 5.5

7 LEU HD1 5 TYR HA 5.5

7 LEU HD1 3 TRP HD1 5.5

7 LEU HD1 5 TYR H 5.5

7 LEU HD1 5 TYR HE1 5.5

1 PGLU HA 3 TRP HD 5.5

8 ARG HG2 10 ET H 5.5

8 ARG HG2 9 PRO HA 5.5

8 ARG HG2 3 TRP HD1 5.5

8 ARG HG2 3 TRP HE3 5.5

8 ARG HG2 3 TRP HZ2 5.5

8 ARG HG2 ? 14MTX H 5.5

Table S12: NOE constraints employed in the NMR refinement protocol for the con3.

#1st_ResID	1st_ResName	1st_H	2nd_ResID	2nd_ResName	2nd_H	upper_dist_limit
2	HIS	HE2	5	TYR	HE1	5.5
7	LEU	HD1	?	LINKER	HST	5.5
7	LEU	HD1	5	TYR	H	5.5
7	LEU	HD1	3	TRP	HD1	5.5
6	LYS	HG	5	TYR	H	5.5
6	LYS	HG	5	TYR	HE1	5.5
4	SER	H	5	TYR	H	5.5
4	SER	H	3	TRP	HD1	5.5
?	1MTX	OH	2	HIS	HD1	5.5
?	1MTX	OH	2	HIS	HE2	5.5
?	1MTX	OH	?	2MTX	H	5.5
?	1MTX	OH	?	5MTX	H	5.5
?	16MTX	H	?	5MTX	NH	5.5

7. Molecular Docking Studies

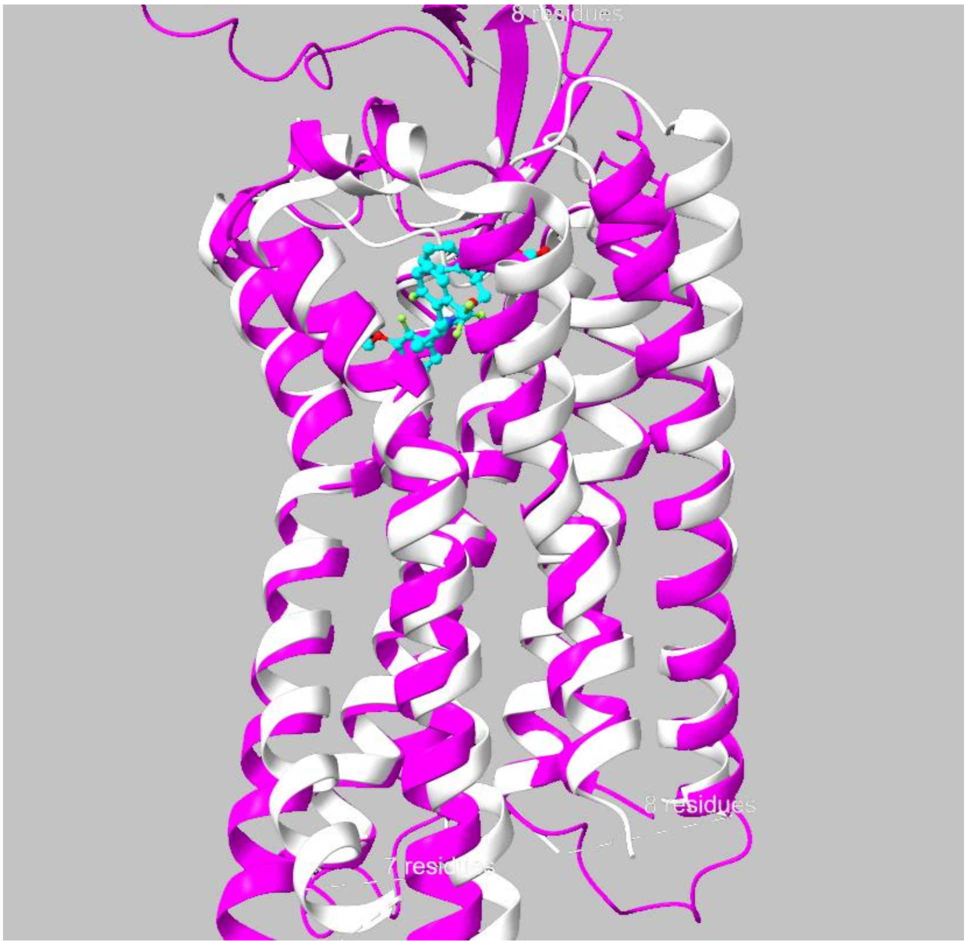

Figure S5: Structural superposition of the human GnRH receptor models (white and magenta cartoon representation). Elagolix molecule (cyan) is located within the binding pocket and was used as a reference for the molecular docking studies.

1. Willcott, M.R. MestRe Nova. *J. Am. Chem. Soc.* **2009**, *131*, 13180–13180, doi:10.1021/ja906709t (Reference [34] in the manuscript).
2. Biniari, G.; Markatos, C.; Nteli, A.; Tzoupis, H.; Simal, C.; Vlamis-Gardikas, A.; Karageorgos, V.; Pirmettis, I.; Petrou, P.; Venihaki, M.; et al. Rational Design, Synthesis and Binding Affinity Studies of Anthraquinone Derivatives Conjugated to Gonadotropin-Releasing Hormone (GnRH) Analogues towards Selective Immunosuppression of Hormone-Dependent Cancer. *Int. J. Mol. Sci.* **2023**, *24*, 15232–15250, doi:10.3390/ijms242015232 (Reference [29] in the manuscript).
3. Laimou, D.K.; Katsara, M.; Matsoukas, M.-T.I.; Apostolopoulos, V.; Troganis, A.N.; Tselios, T.V. Structural Elucidation of Leuprolide and Its Analogues in Solution: Insight into Their Bioactive Conformation. *Amino Acids* **2010**, *39*, 1147–1160, doi:10.1007/s00726-010-0549-8 (Reference [33] in the manuscript).
