# Supplementary material for: NMR Structural Elucidation of Mitoxantrone–Gonadotropin-Releasing Hormone (GnRH) Conjugates Implicated in Hormone-Dependent Cancer
Source: Int J Mol Sci. 2026 Aug 20;27(16):7437. doi: 10.3390/ijms27167437 (PMC13513673; doi:10.3390/ijms27167437)
Supplement: Supplementary file 1 [file ijms-27-07437-s001.zip › SI_SI2_NMR SPECTRA_revised.pdf]

NMR Structural Elucidation of Mitoxantrone–Gonadotropin–Releasing Hormone (GnRH) Conjugates Implicated in Hormone–Dependent Cancer

Georgia Biniari [1], Haralambos Tzoupis [1], Uroš Javornik [2], Nikitas Georgiou [3,4], Georgios Liapakis [5], Thomas Mavromoustakos [3], Theodore Tselios [1] and Carmen Simal [1,*]

[1]Department of Chemistry, University of Patras, 26504 Rion Patras, Greece

[2]Slovenian NMR Centre, National Institute of Chemistry, SI-1001 Ljubljana, Slovenia

[3]Department of Chemistry, National and Kapodistrian University of Athens, 10679, Athens, Greece

[4]Center for Interdisciplinary Biosciences, Technology and Innovation Park, P.J. Safarik University in Kosice, Jesenna 5, 04001 Kosice, Slovakia

[5]Department of Pharmacology, School of Medicine, University of Crete, 71003 Heraklion, Greece

Table of Contents

Figure S6: ^{1}H (A) and ^{13}C (B) NMR spectra of con3 peptide in HDO. S3

Figure S7: ^{1}H-^{1}H DQF COSY spectrum of con3 peptide in HDO. S4

Figure S8: ^{1}H-^{1}H TOCSY spectrum of con3 peptide in HDO S4

Figure S9: ^{1}H-^{1}H NOESY spectrum of con3 peptide in HDO. S5

Figure S10: ^{1}H-^{13}C HSQC spectrum of con3 peptide in HDO S5

Figure S11: ^{1}H-^{13}C HMBC spectrum of con3 peptide in HDO. S6

Figure S12: ^{1}H (A) and ^{13}C (B) NMR spectra of con3 peptide in DMSO-d_6. ....S7

Figure S13: ^{1}H-^{1}H TOCSY spectrum of con3 peptide in DMSO-d_6. ....S8

Figure S14: ^{1}H-^{1}H zqf NOESY spectrum of con3 peptide in DMSO-d_6. ....S8

Figure S15: ^{1}H-^{15}N HSQC spectrum of con3 peptide in DMSO-d_6. ....S9

Figure S16: ^{1}H-^{13}C HMBC spectrum of con3 peptide in DMSO-d_6. ....S9

Figure S17: ^{1}H (A) and ^{13}C (B) NMR spectra of con3 in HDO. S10

Figure S18: ^{1}H-^{1}H DQF COSY spectrum of con3 in HDO. S11

Figure S19: ^{1}H-^{1}H TOCSY spectrum of con3 in HDO S11

Figure S20: ^{1}H-^{1}H NOESY spectrum of con3 in HDO. S12

Figure S21: ^{1}H-^{13}C HSQC spectrum of con3 in HDO. S12

Figure S22: ^{1}H-^{13}C HMBC spectrum of con3 in HDO. S13

Figure S23: ^{1}H NMR spectrum of con3 in DMSO-d_6. ....S13

Figure S24: ^{1}H-^{1}H zqf NOESY spectrum of con3 in DMSO-d_6 ....S14

Figure S25: ^{1}H-^{15}N HSQC spectrum of con3 in DMSO-d_6. ....S14

Figure S26: ^{1}H (A) and ^{13}C (B) NMR spectra of con7 peptide in HDO. S15

Figure S27: ^{1}H-^{1}H DQF COSY spectrum of con7 peptide in HDO S16
Figure S28: ^{1}H-^{1}H TOSY spectrum of con7 peptide in HDO. S16
Figure S29: ^{1}H-^{1}H NOESY spectrum of con7 peptide in HDO. S17
Figure S30: ^{1}H-^{13}C HSQC spectrum of con7 peptide in HDO. S17
Figure S31: ^{1}H-^{13}C HMBC spectrum of con7 peptide in HDO. S18
Figure S32: ^{1}H (A) and ^{13}C (B) NMR spectra of con7 peptide in DMSO-d_6 .... S19
Figure S33: ^{1}H-^{1}H TOCSY spectrum of con7 peptide in DMSO-d_6. .... S20
Figure S34: ^{1}H-^{1}H zqf NOESY spectrum of con7 peptide in DMSO-d_6. .... S20
Figure S35: ^{1}H-^{15}N HSQC spectrum of con7 peptide in DMSO-d_6. .... S21
Figure S36: ^{1}H-^{13}C HMBC spectrum of con7 peptide in DMSO-d_6. .... S21
Figure S37: ^{1}H (A) and ^{13}C (B) NMR spectra of con7 in HDO S22
Figure S38: ^{1}H-^{1}H DQF COSY spectrum of con7 in HDO. S23
Figure S39: ^{1}H-^{1}H TOSY spectrum of con7 in HDO. S23
Figure S40: ^{1}H-^{1}H NOESY spectrum of con7 in HDO. S24
Figure S41: ^{1}H-^{13}C HSQC spectrum of con7 in HDO. S24
Figure S42: ^{1}H-^{13}C HMBC spectrum of con7 in HDO. S25
Figure S43: ^{1}H NMR spectrum of con7 in DMSO-d_6. .... S25
Figure S44: ^{1}H-^{1}H zqf NOESY spectrum of con7 in DMSO-d_6. .... S26
Figure S45: ^{1}H-^{15}N HSQC spectrum of con7 in DMSO-d_6. .... S26

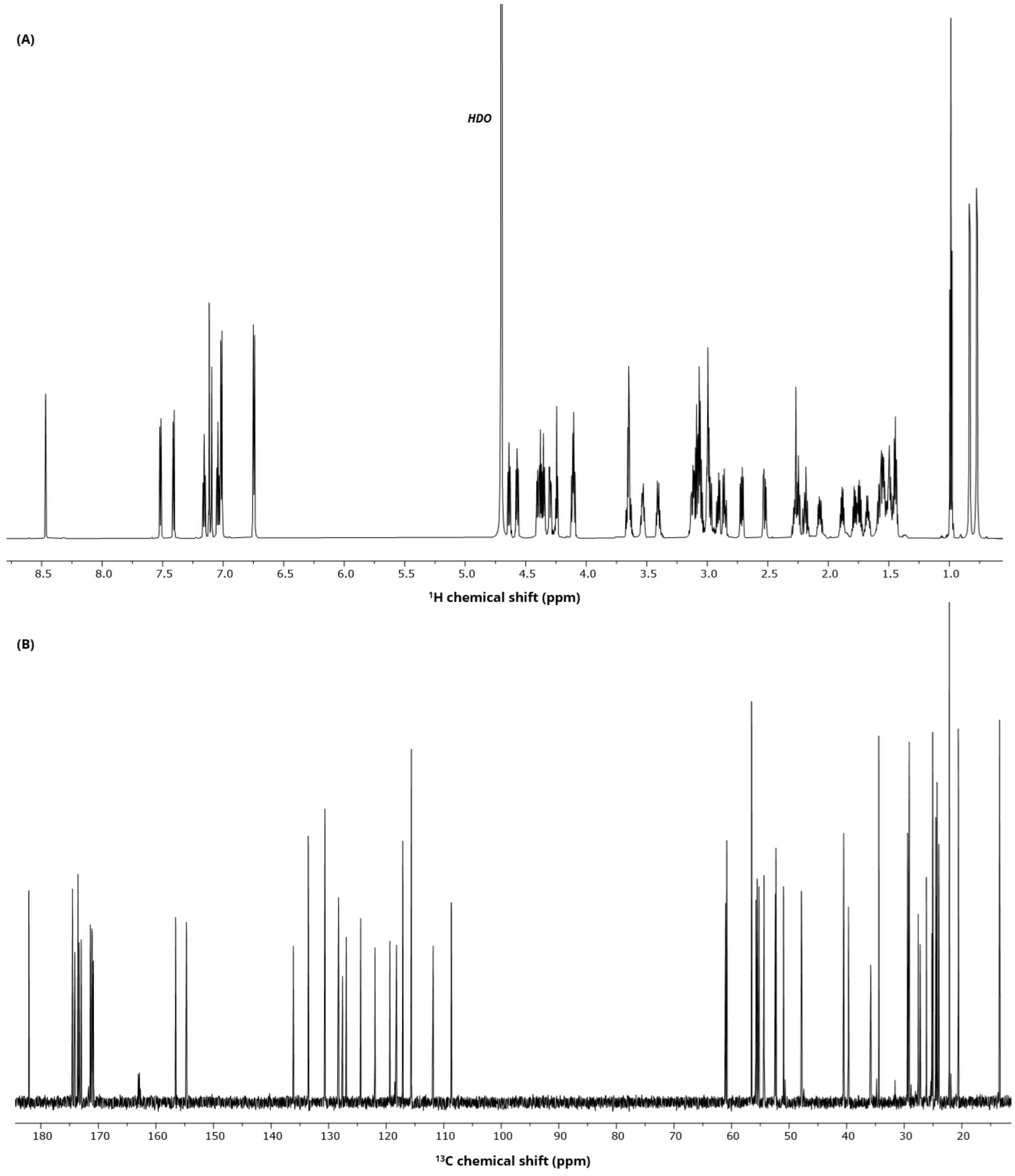

Figure S6: ^{1}H (A) and ^{13}C (B) NMR spectra of con3 peptide (800 MHz, HDO, 298 K).

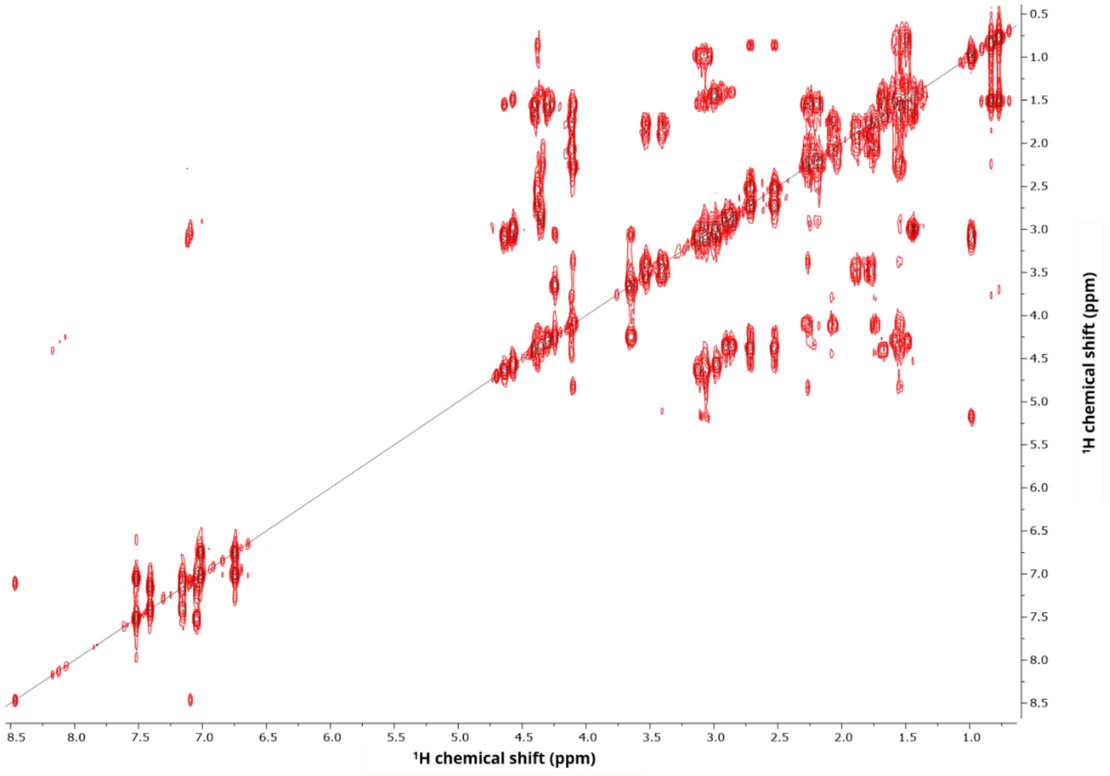

Figure S7: ^{1}H-^{1}H DQF COSY spectrum of con3 peptide (800 MHz, HDO, 298 K).

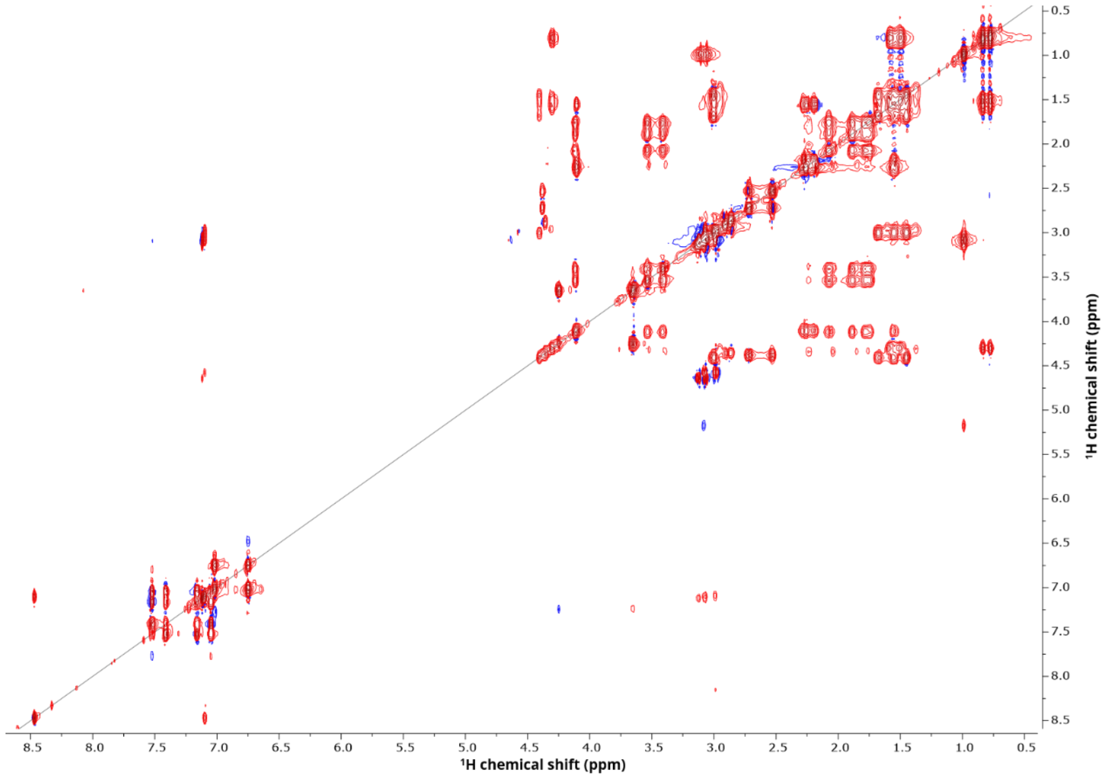

Figure S8: ^{1}H-^{1}H TOCSY spectrum of con3 peptide (800 MHz, HDO, 298 K, t_m = 80 ms).

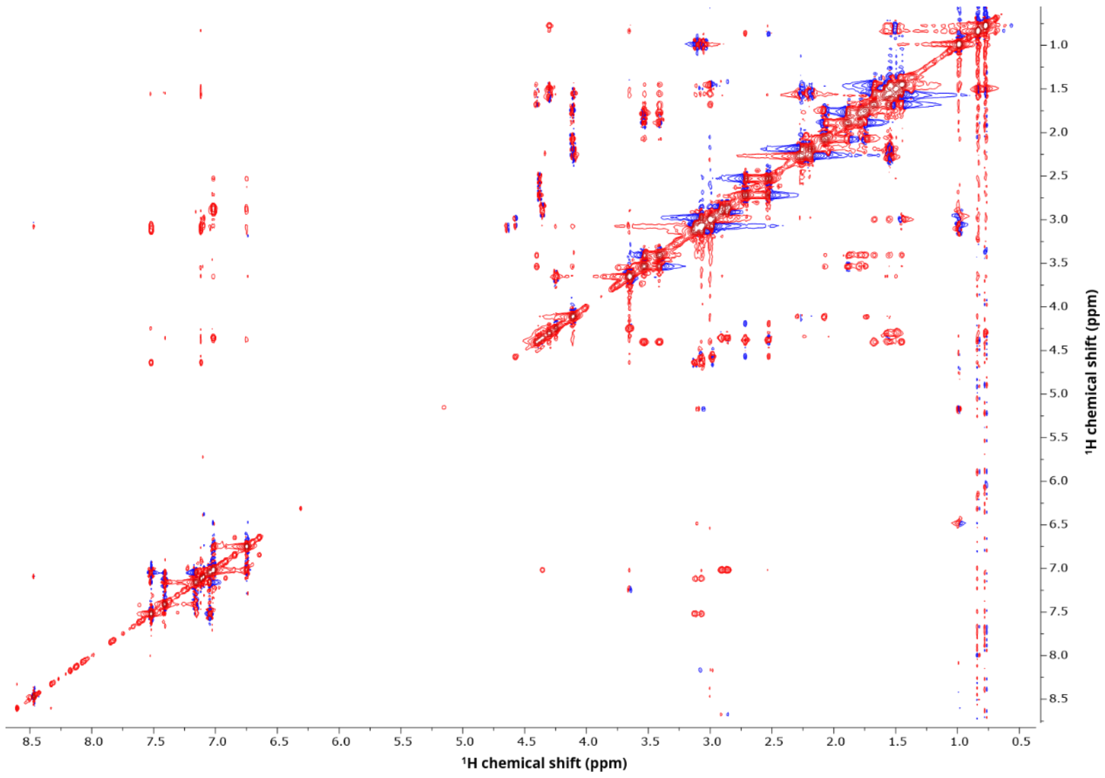

Figure S9: ^{1}H-^{1}H NOESY spectrum of con3 peptide (800 MHz, HDO, 298 K, 300 ms).

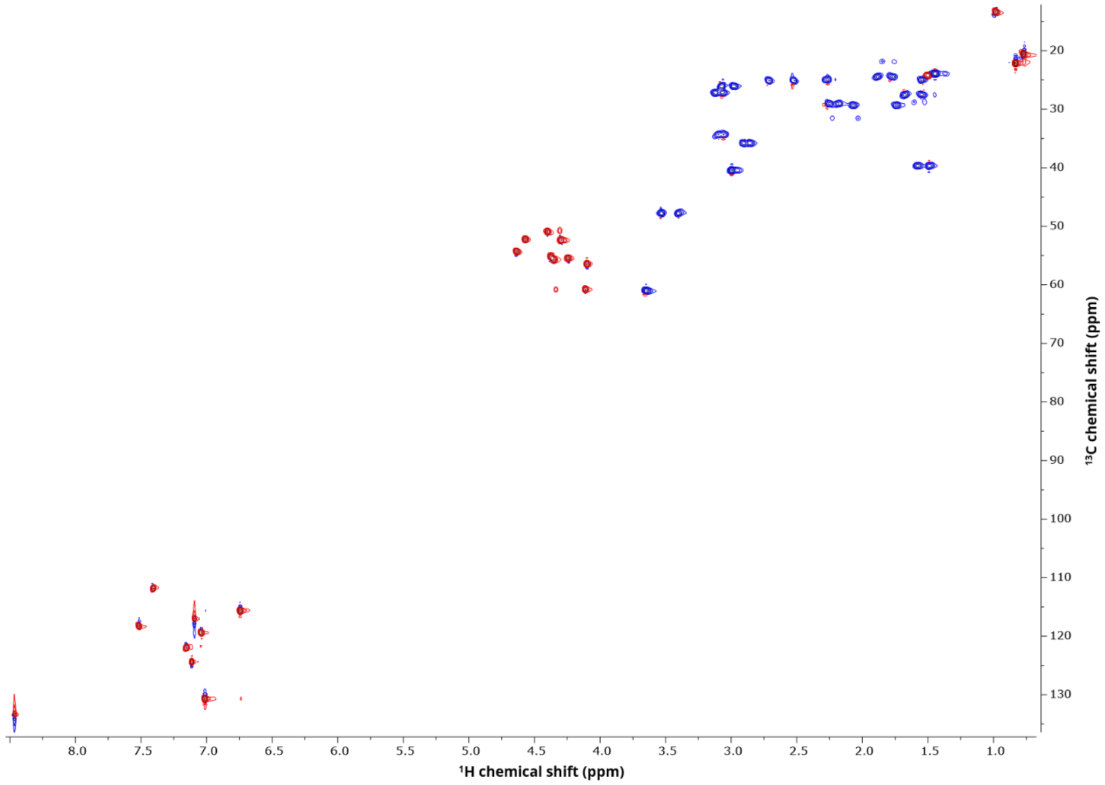

Figure S10: ^{1}H-^{13}C HSQC spectrum of con3 peptide (800 MHz, HDO, 298 K).

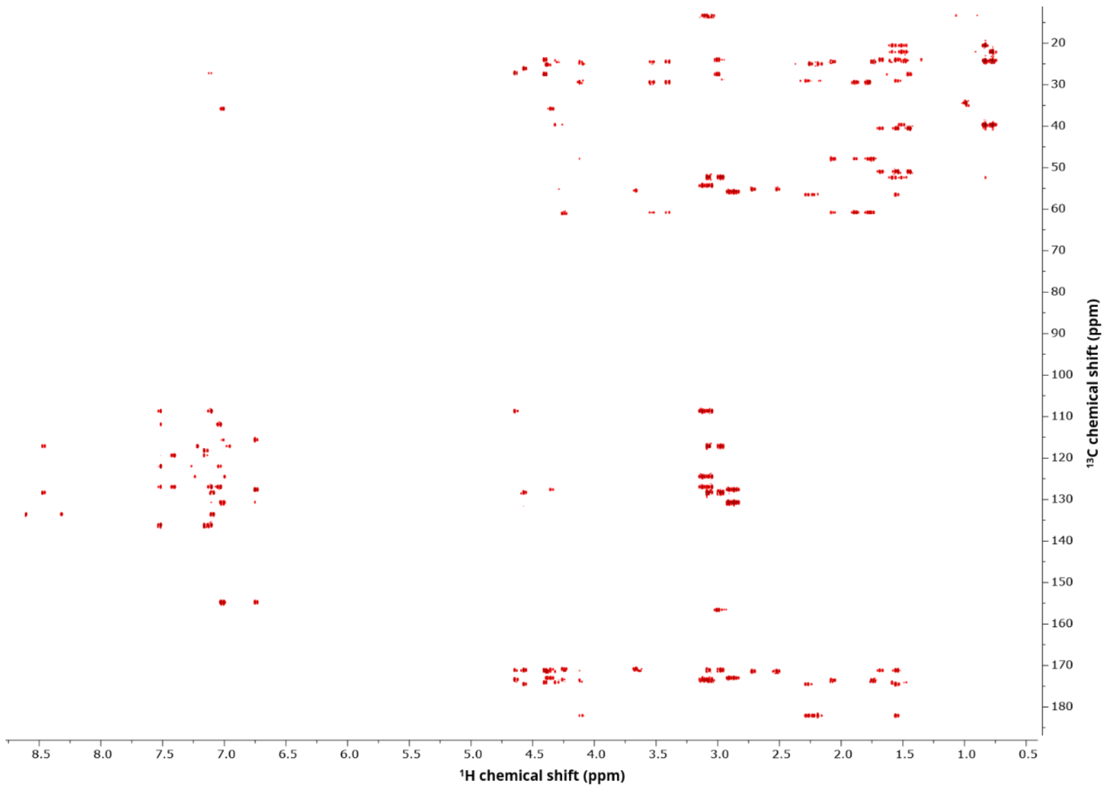

Figure S11: ^{1}H-^{13}C HMBC spectrum of con3 peptide (800 MHz, HDO, 298 K).

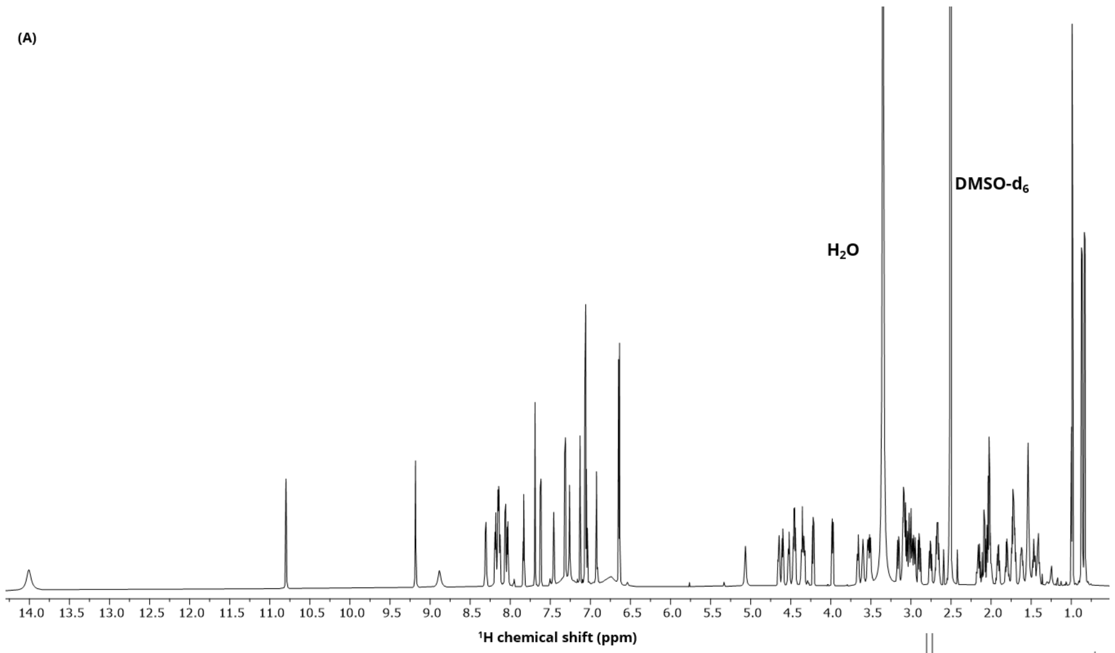

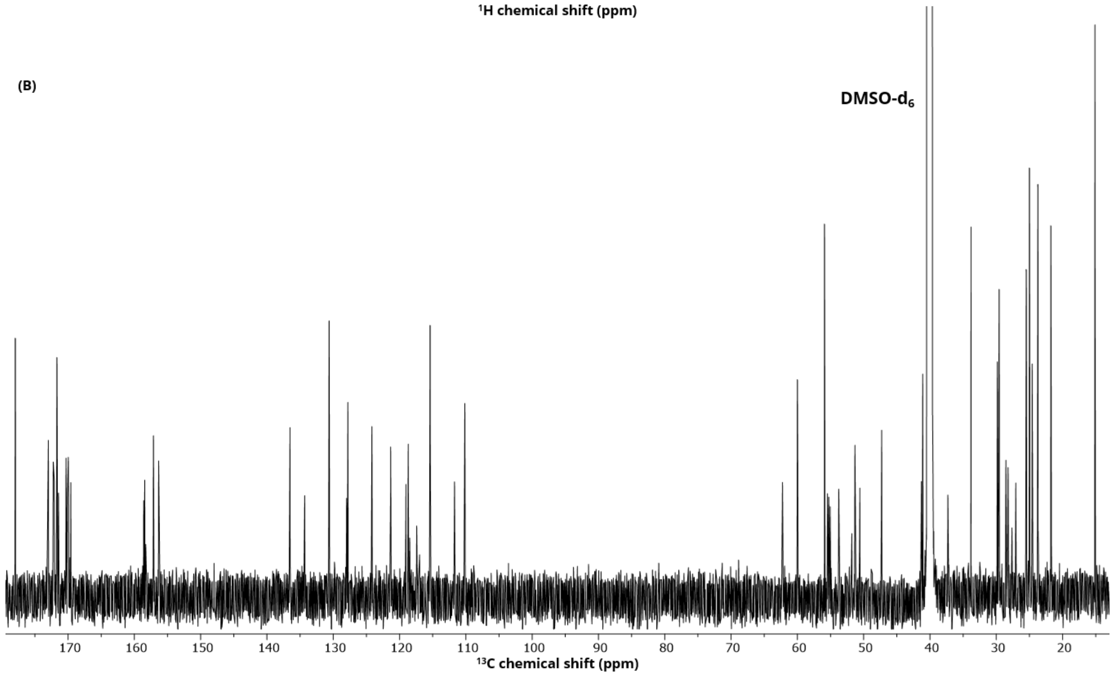

Figure S12: ^{1}H (A) and ^{13}C (B) NMR spectra of con3 peptide (800 MHz, DMSO-d_6, 298 K).

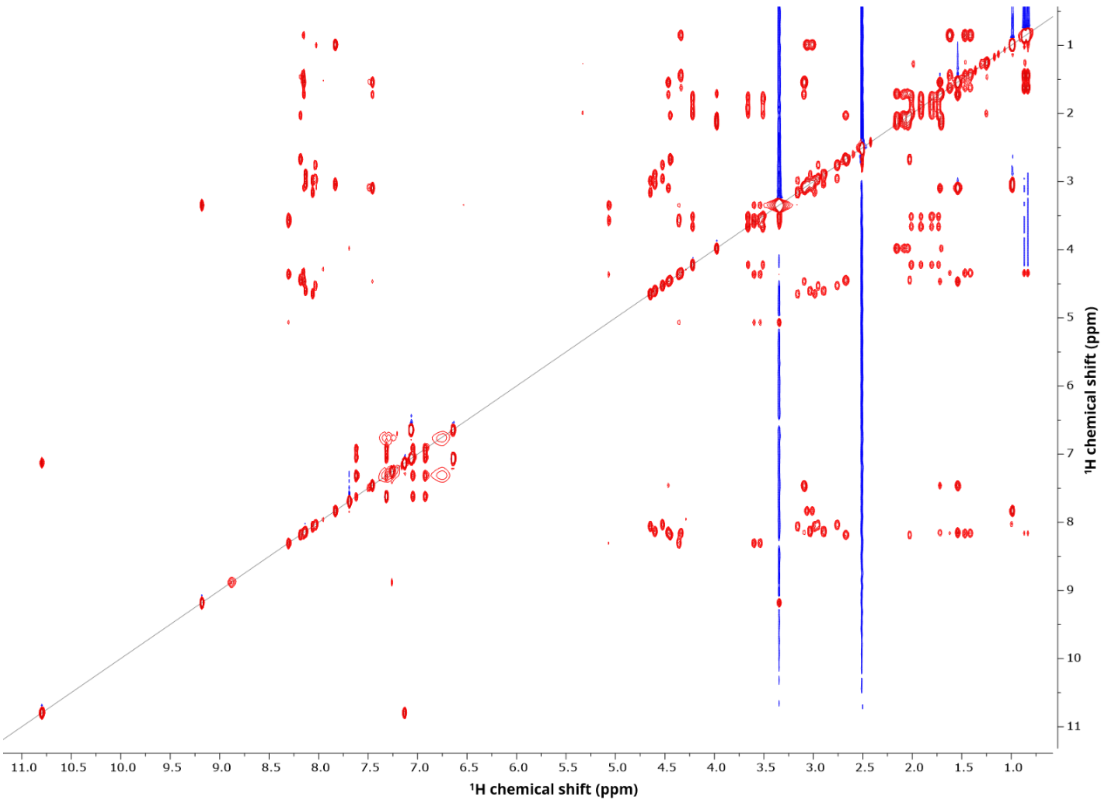

Figure S13: ^{1}H-^{1}H TOCSY spectrum of con3 peptide (800 MHz, DMSO-d_6, 298 K, t_m = 80 ms).

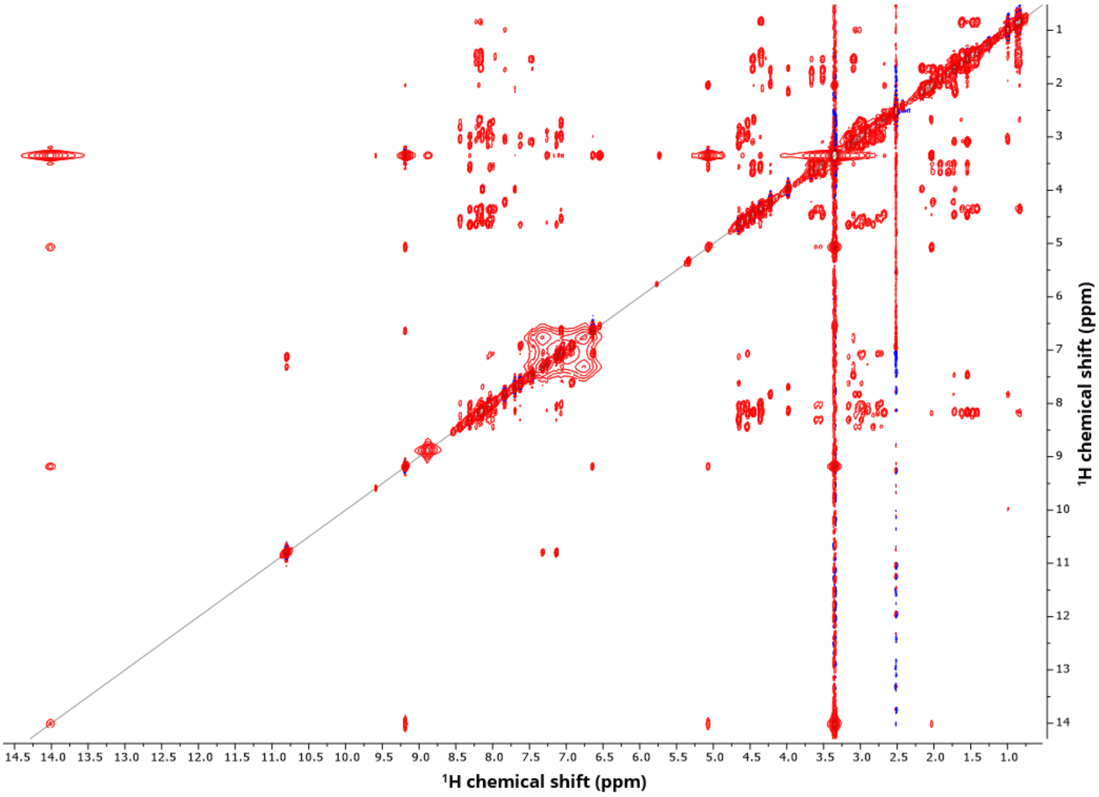

Figure S14: ^{1}H-^{1}H zqf NOESY spectrum of con3 peptide (800 MHz, DMSO-d_6, 298 K, 150 ms).

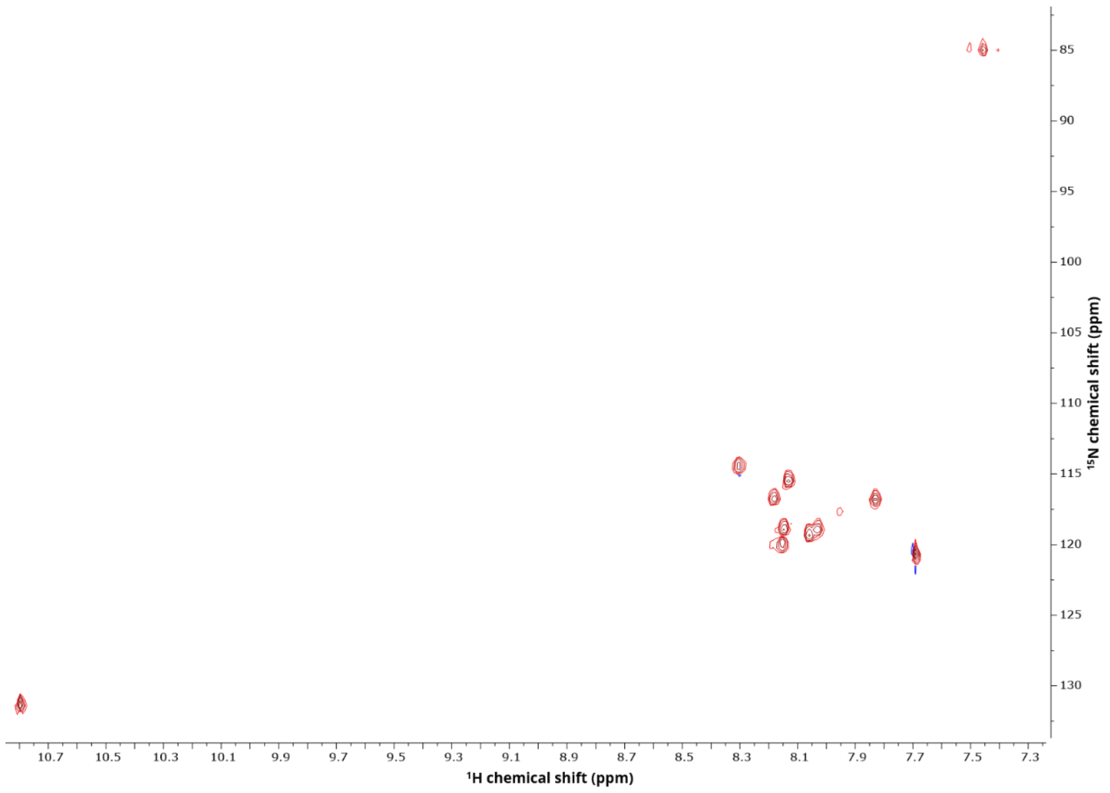

Figure S1: ¹H-¹⁵N HSQC spectrum of con3 peptide (800 MHz, DMSO-d_6, 298 K).

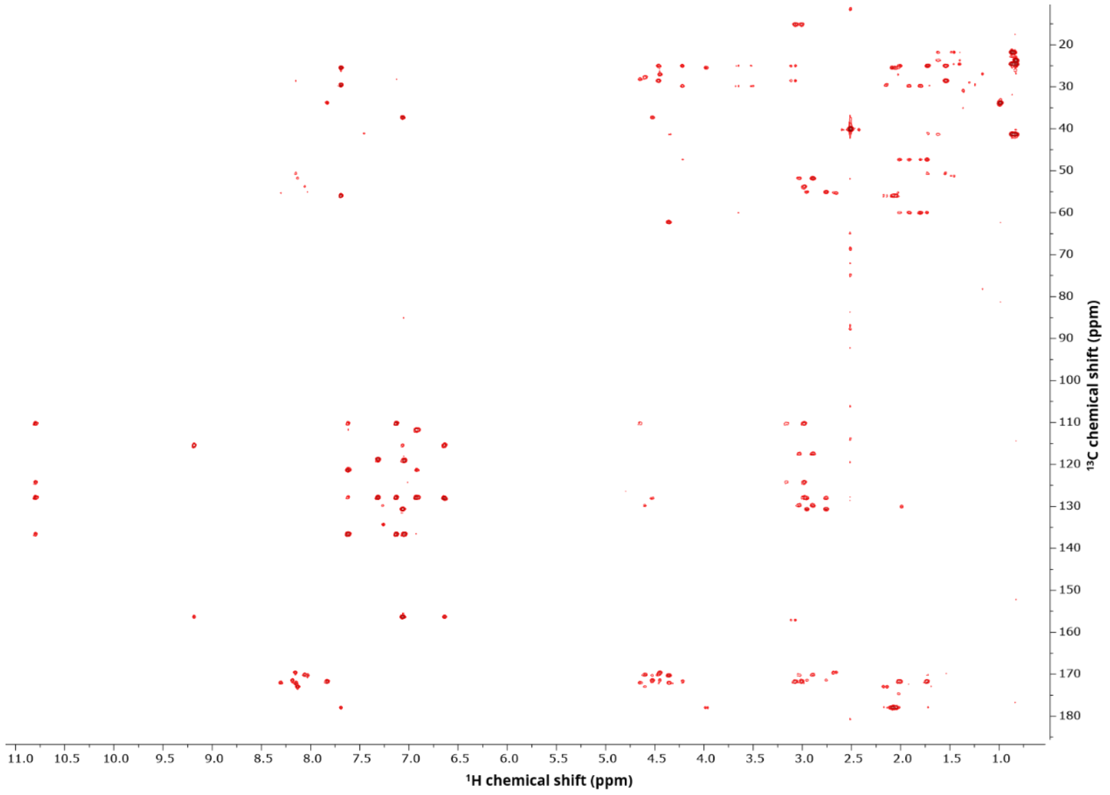

Figure S16: ¹H-¹³C HMBC spectrum of con3 peptide (800 MHz, DMSO-d_6, 298 K).

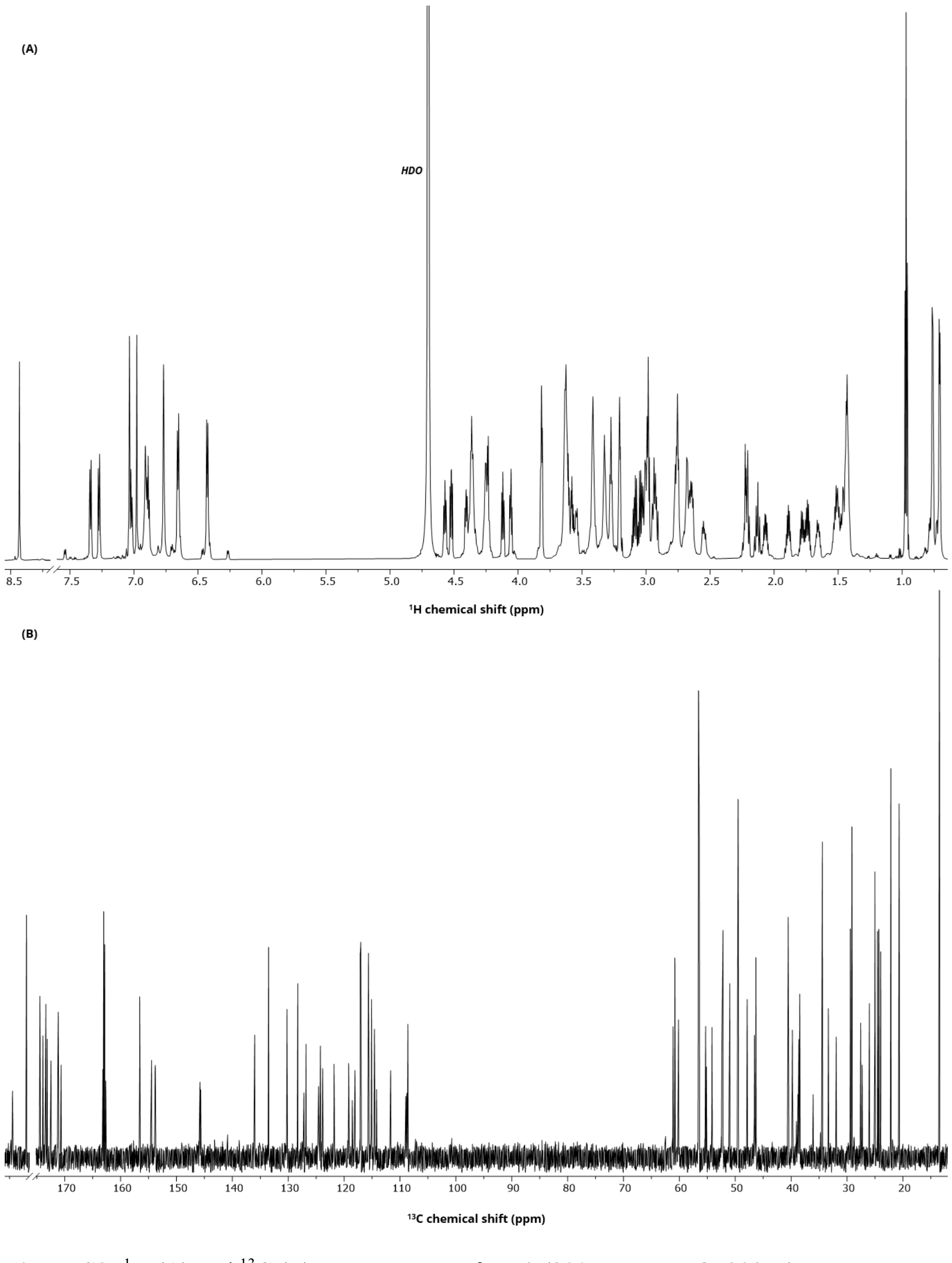

Figure S2: ^{1}H (A) and ^{13}C (B) NMR spectra of con3 (800 MHz, HDO, 298 K).

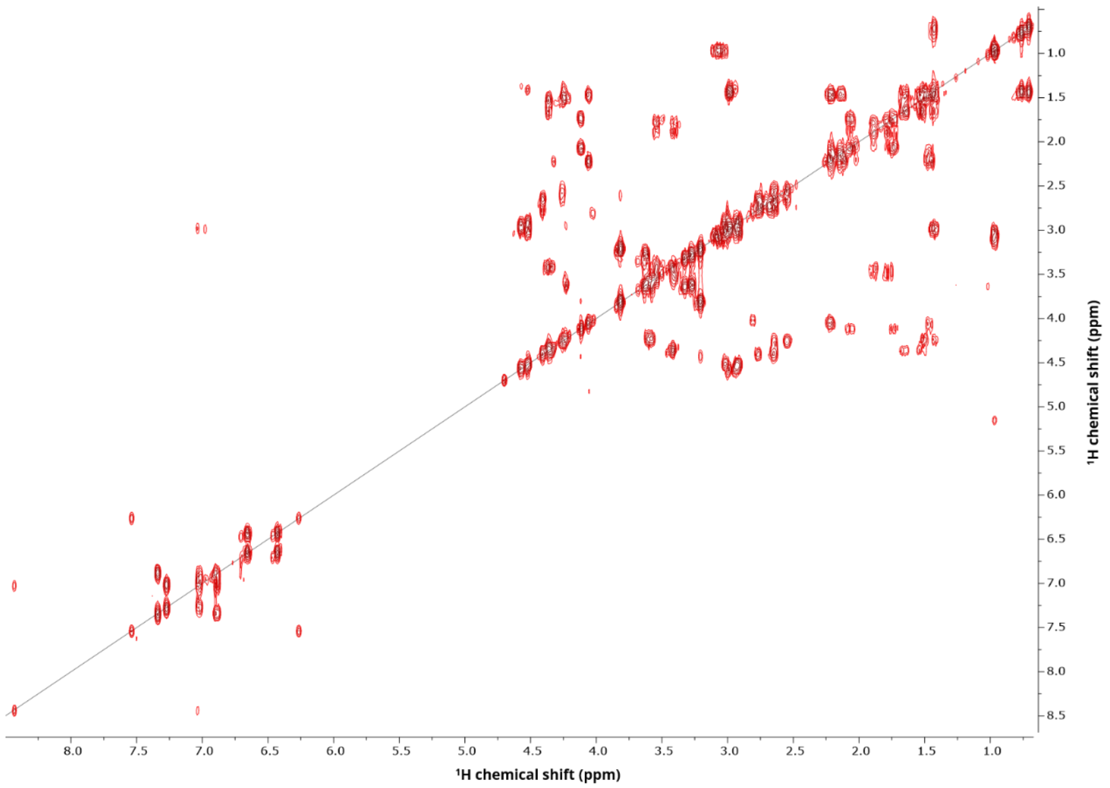

Figure S3: ^{1}H-^{1}H DQF COSY spectrum of con3 (800 MHz, HDO, 298 K).

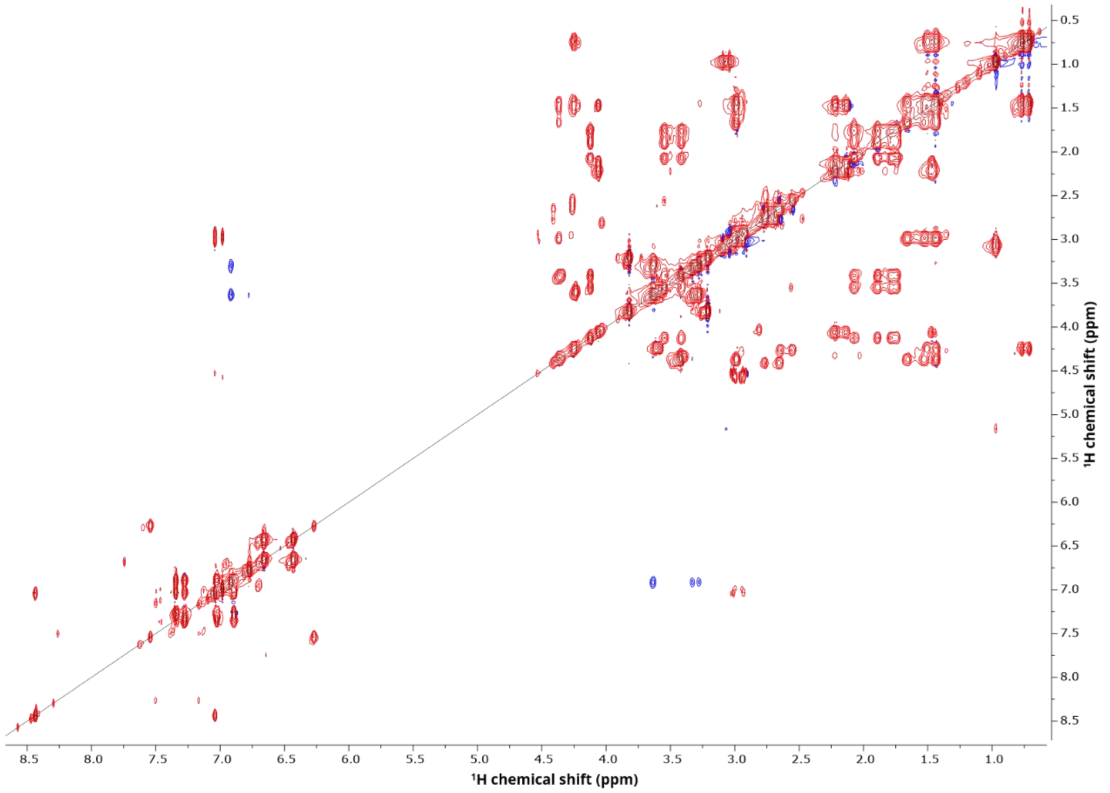

Figure S4: ^{1}H-^{1}H TOCSY spectrum of con3 (800 MHz, HDO, 298 K, t_m = 80 ms).

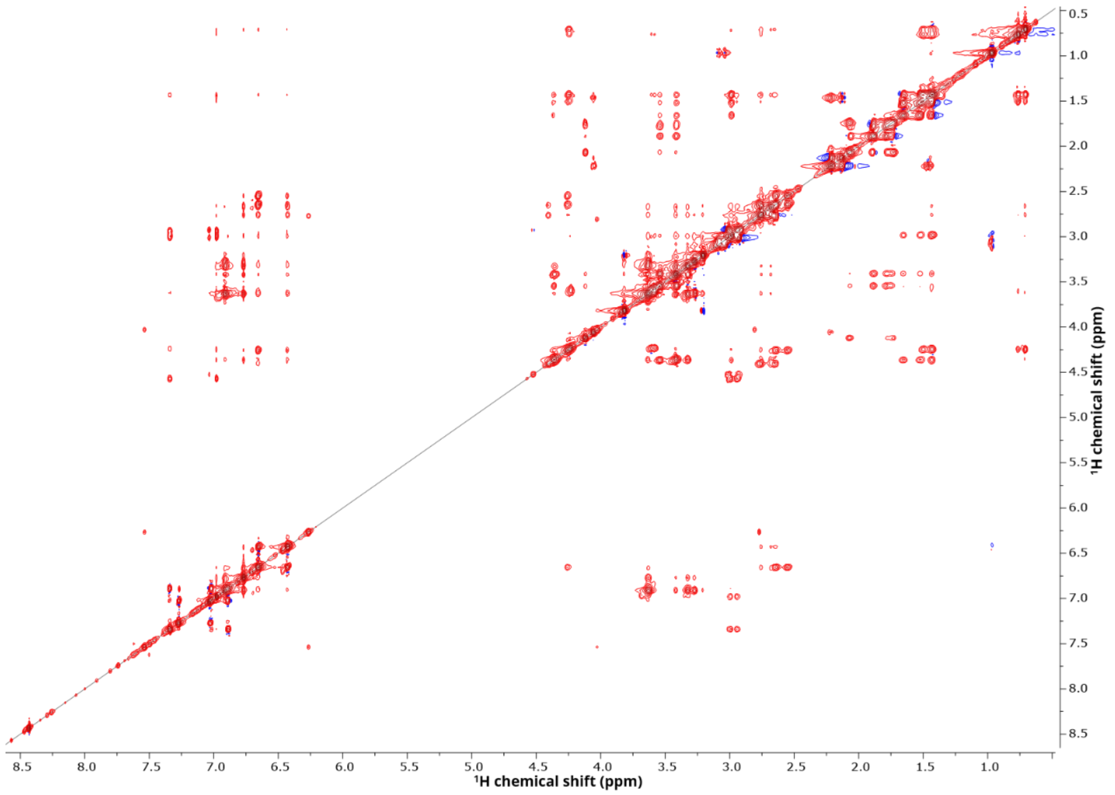

Figure S20: ^{1}H-^{1}H NOESY spectrum of con3 (800 MHz, HDO, 298 K, 300 ms).

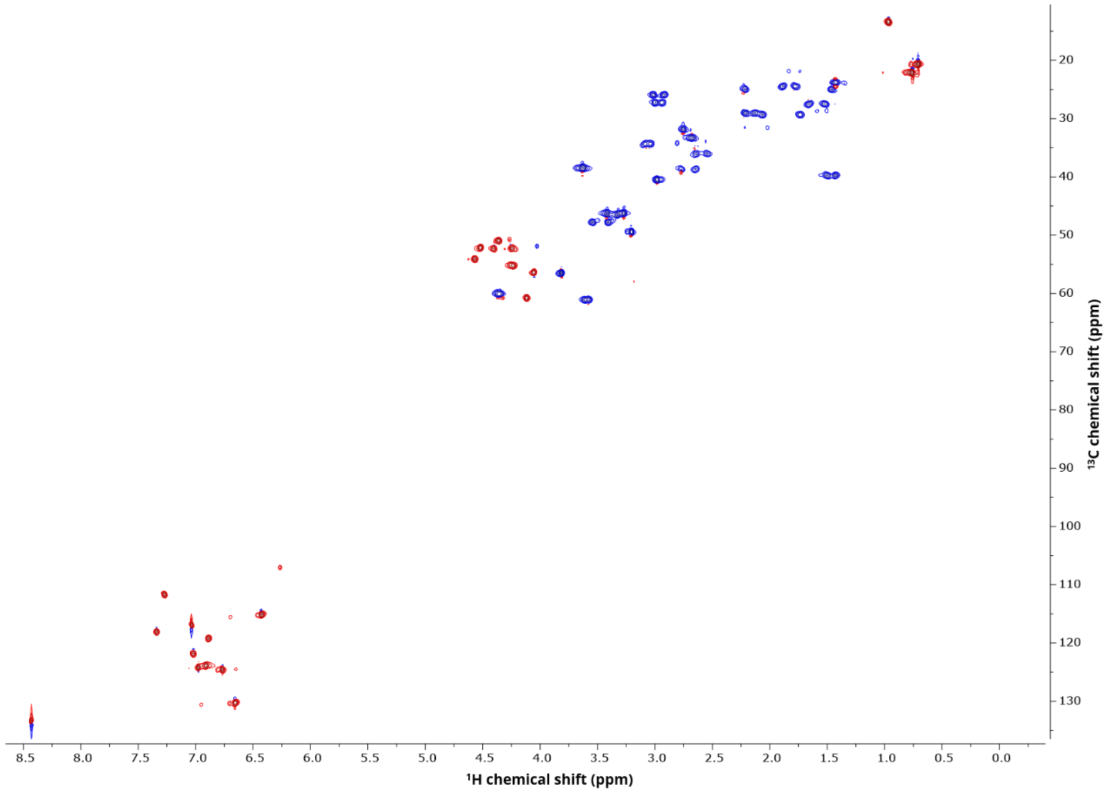

Figure S21: ^{1}H-^{13}C HSQC spectrum of con3 (800 MHz, HDO, 298 K).

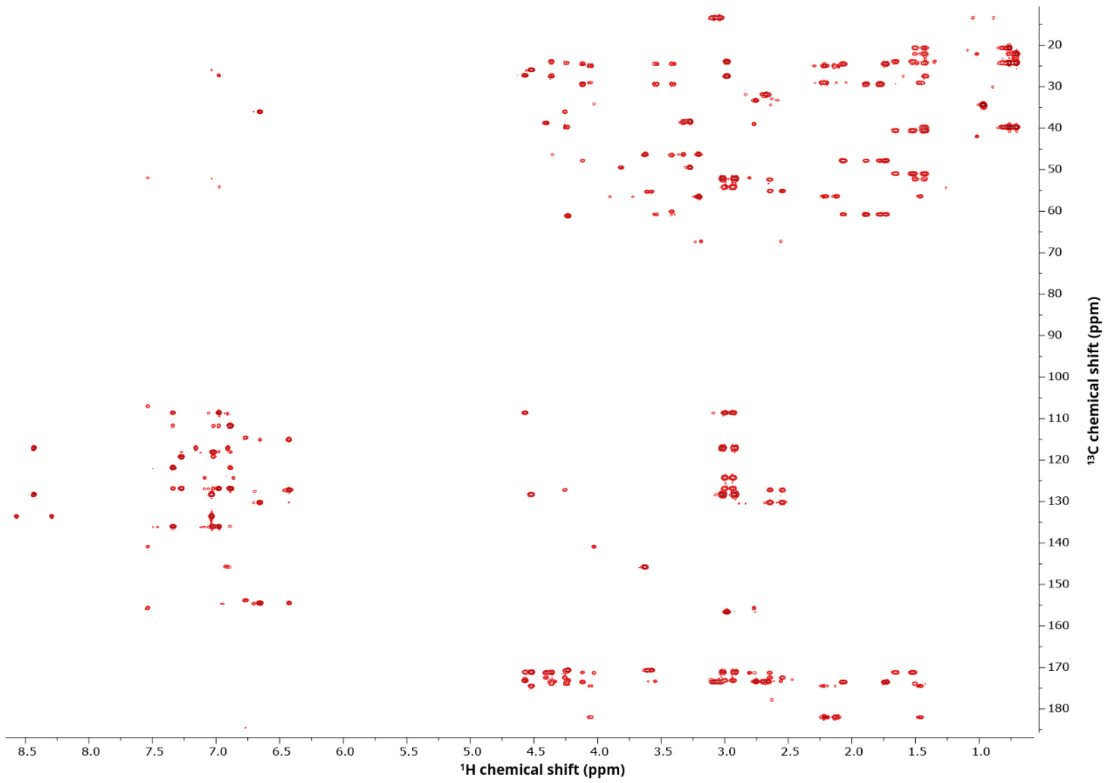

Figure S22: ^{1}H-^{13}C HMBC spectrum of con3 (800 MHz, HDO, 298 K).

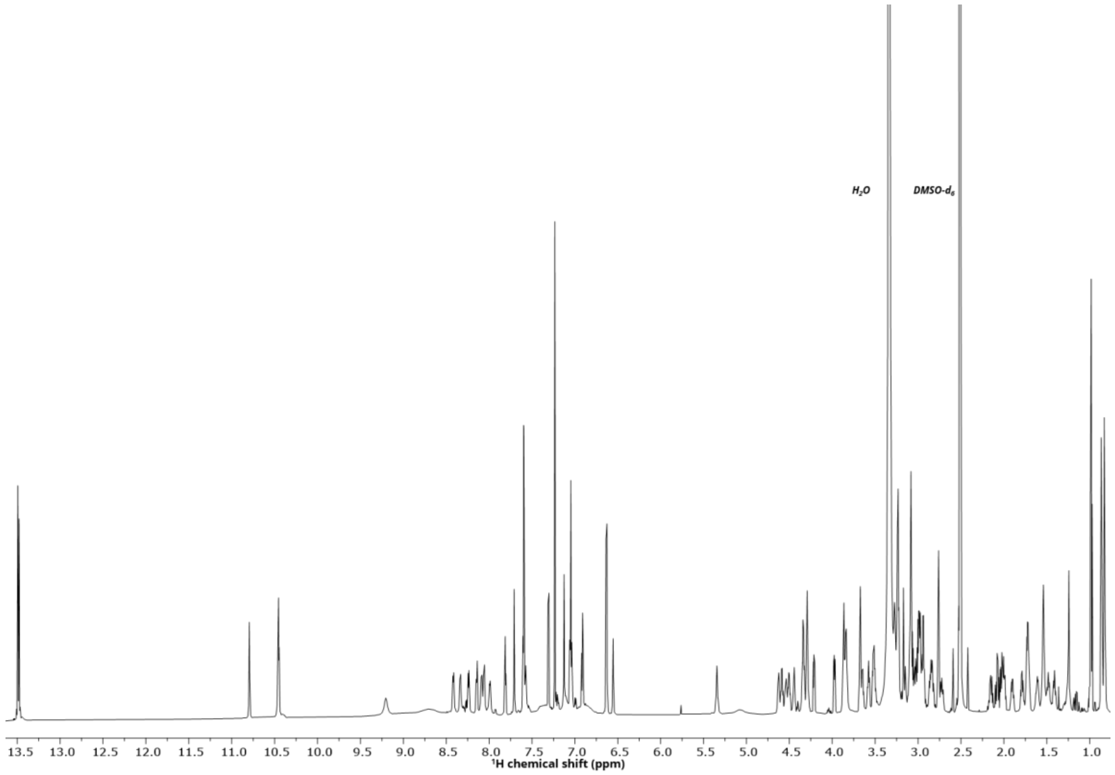

Figure S23: ^{1}H NMR spectrum of con3 (800 MHz, DMSO-d_6, 298 K).

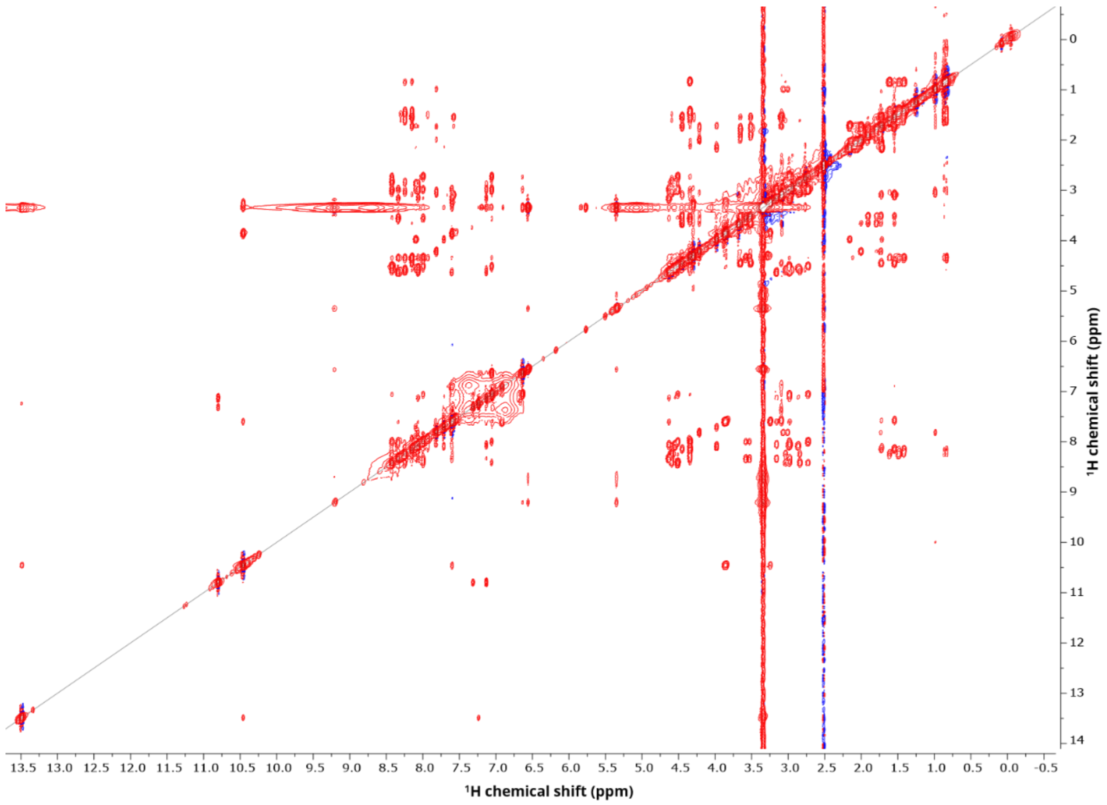

Figure S24: ^{1}H-^{1}H zqf NOESY spectrum of con3 (800 MHz, DMSO-d_6, 298 K, 150 ms).

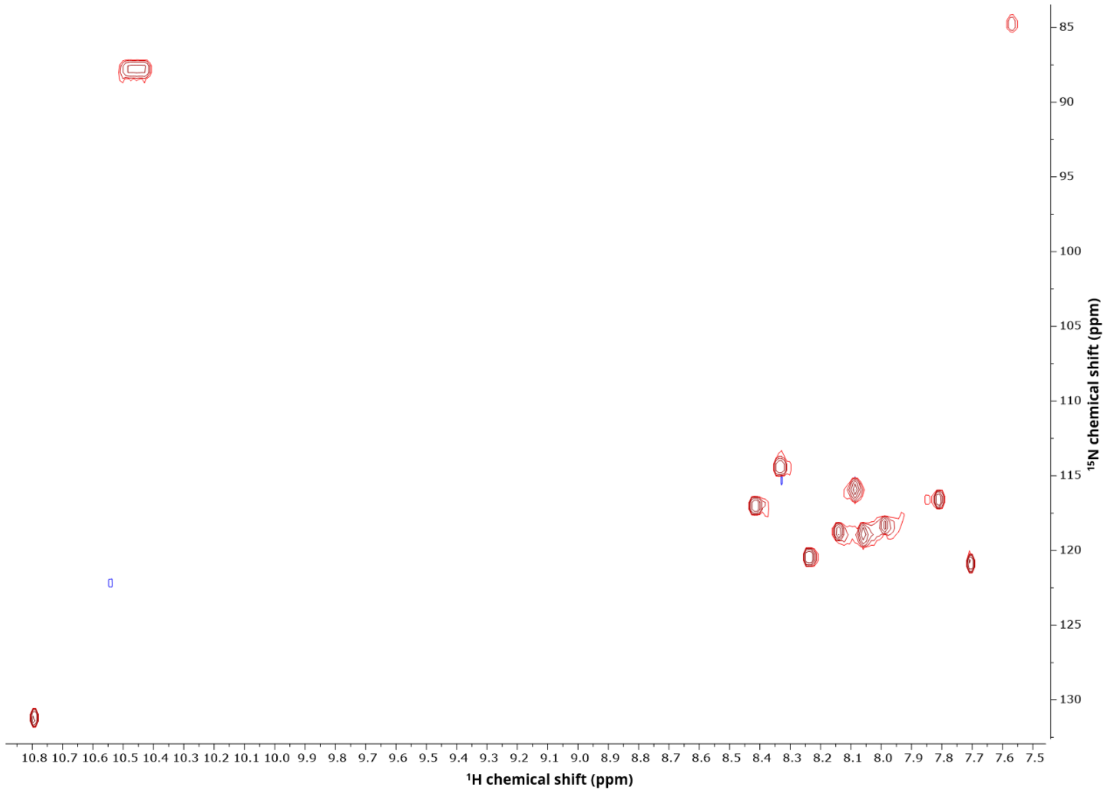

Figure S5: ^{1}H-^{15}N HSQC spectrum of con3 (800 MHz, DMSO-d_6, 298 K).

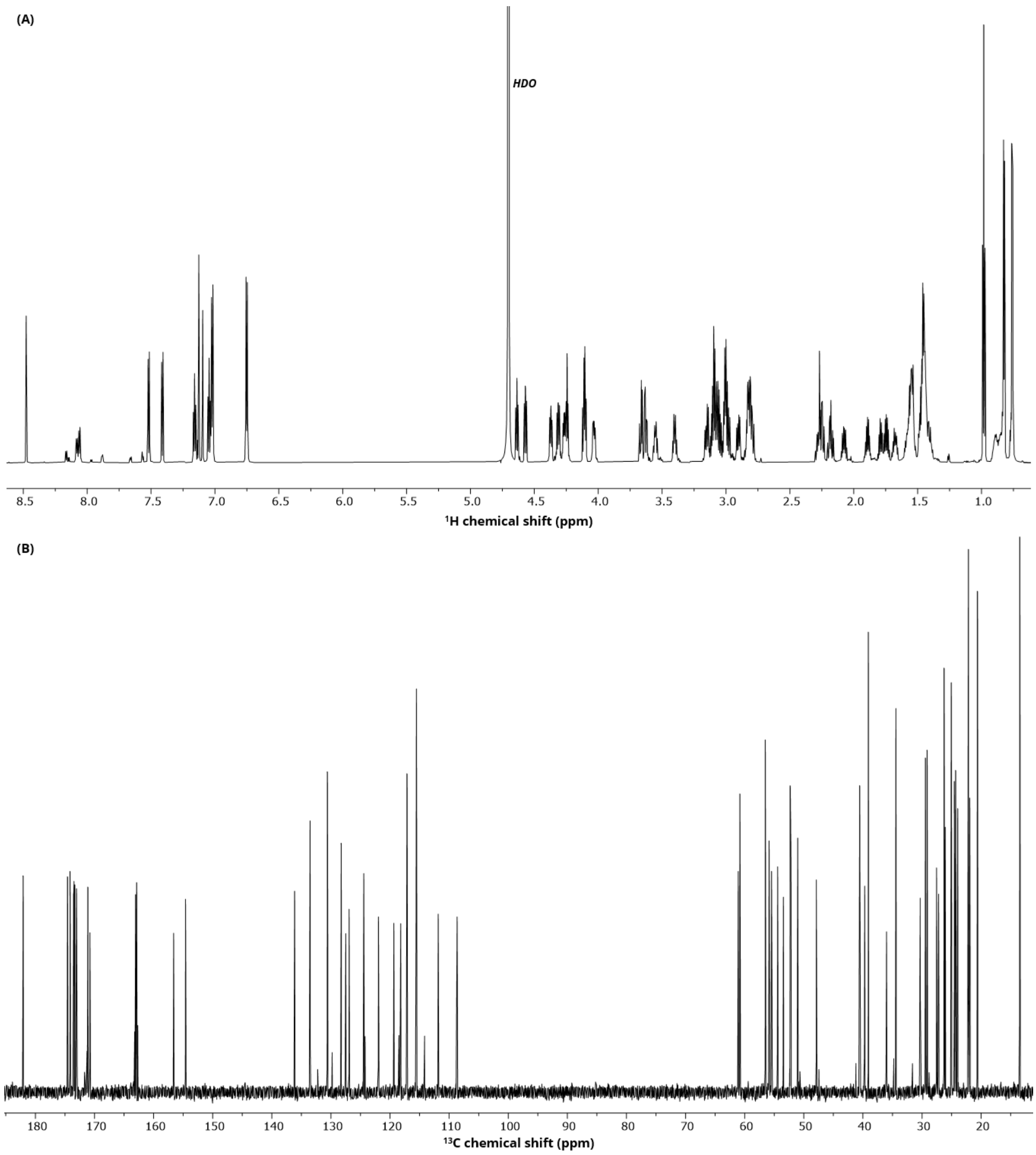

Figure S6: ^{1}H (A) and ^{13}C (B) NMR spectra of con7 peptide (800 MHz, HDO, 298 K).

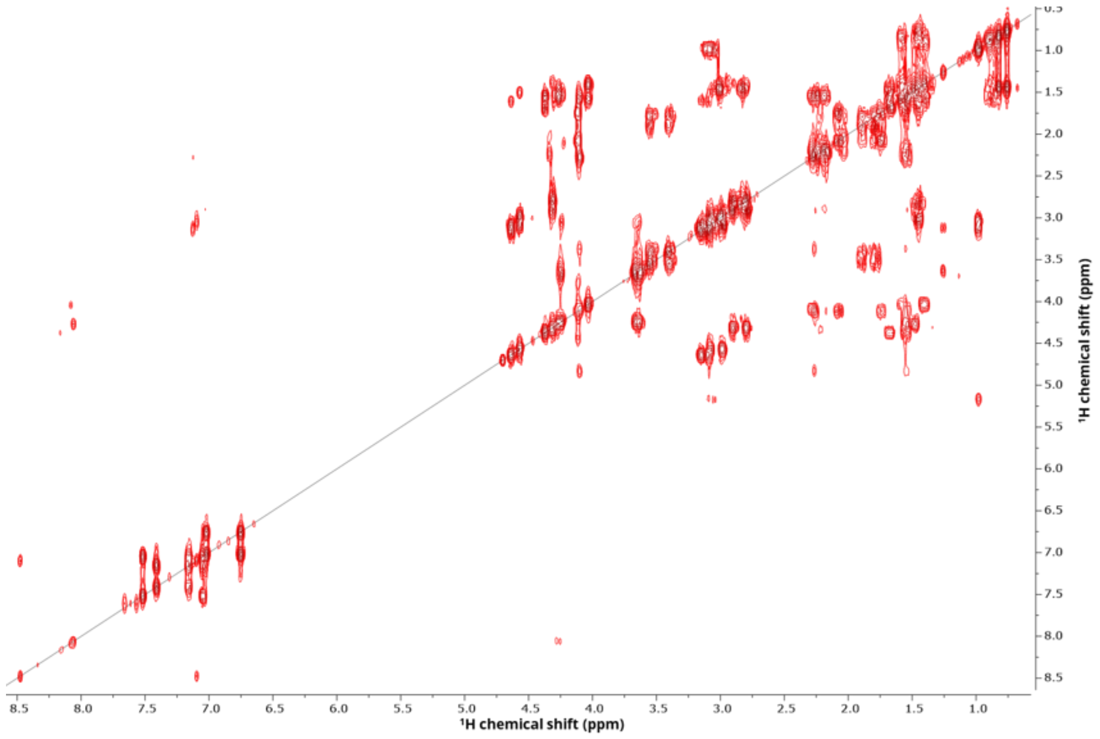

Figure S7: ^{1}H-^{1}H DQF COSY spectrum of con7 peptide (800 MHz, HDO, 298 K).

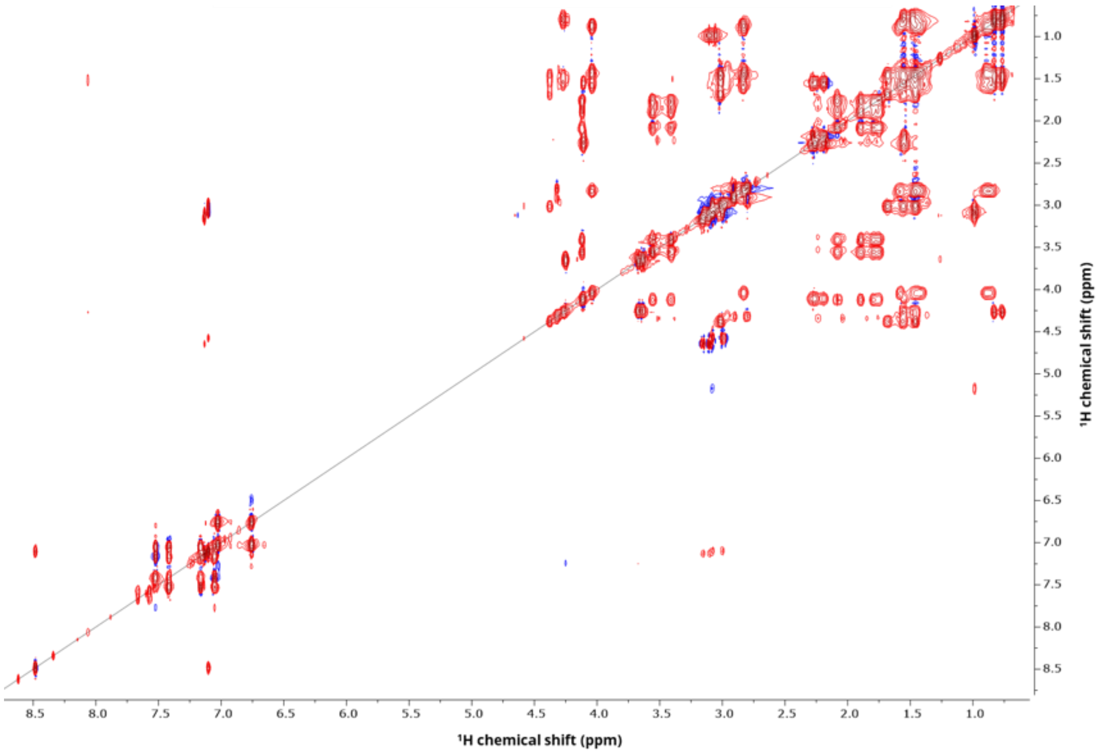

Figure S8: ^{1}H-^{1}H TOSY spectrum of con7 peptide (800 MHz, HDO, 298 K, t_m = 80 ms).

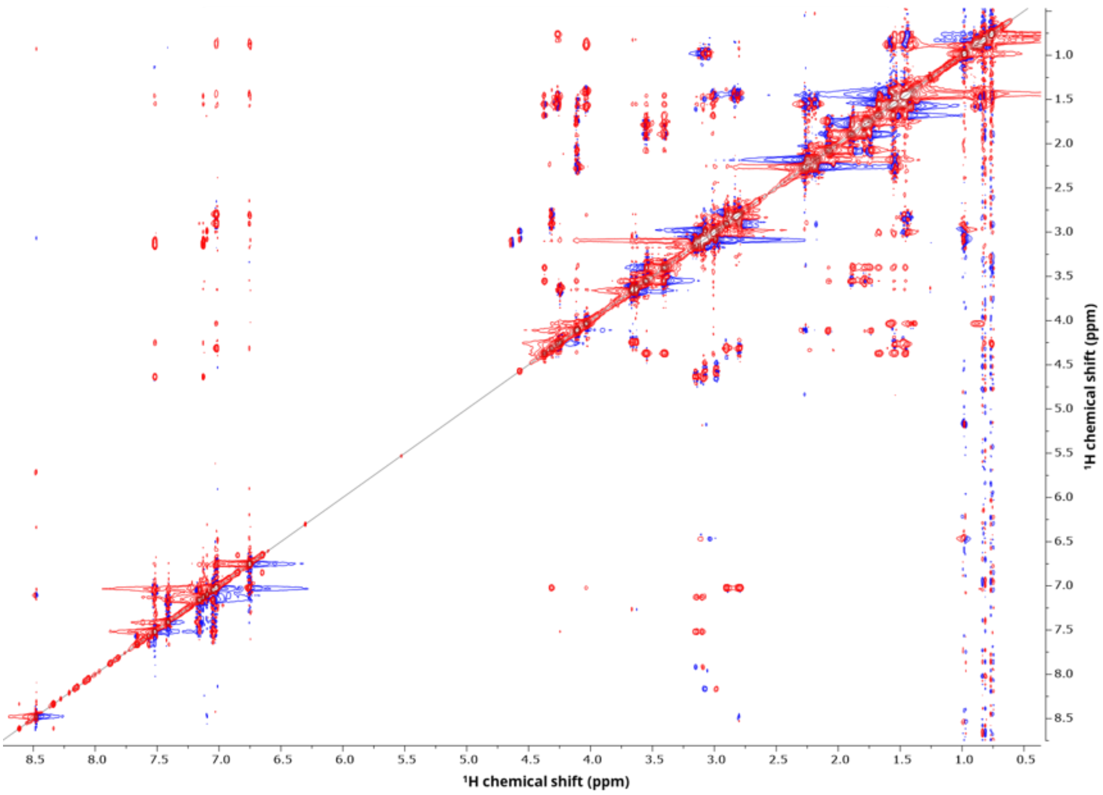

Figure S9: ^{1}H-^{1}H NOESY spectrum of con7 peptide (800 MHz, HDO, 298 K, 300 ms).

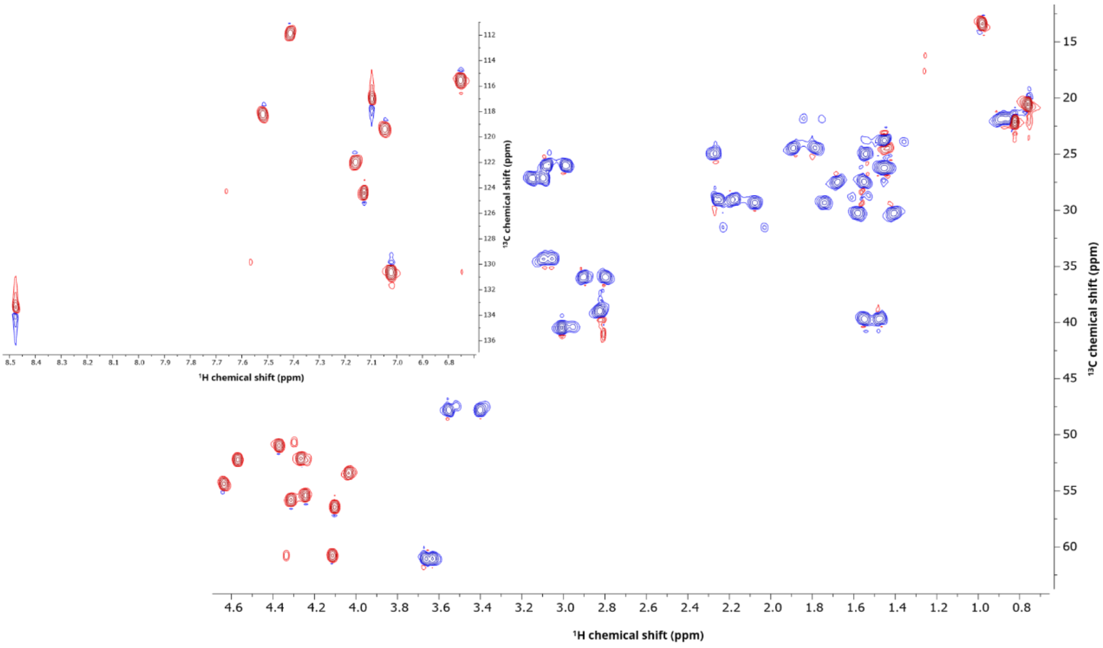

Figure S30: ^{1}H-^{13}C HSQC spectrum of con7 peptide (800 MHz, HDO, 298 K).

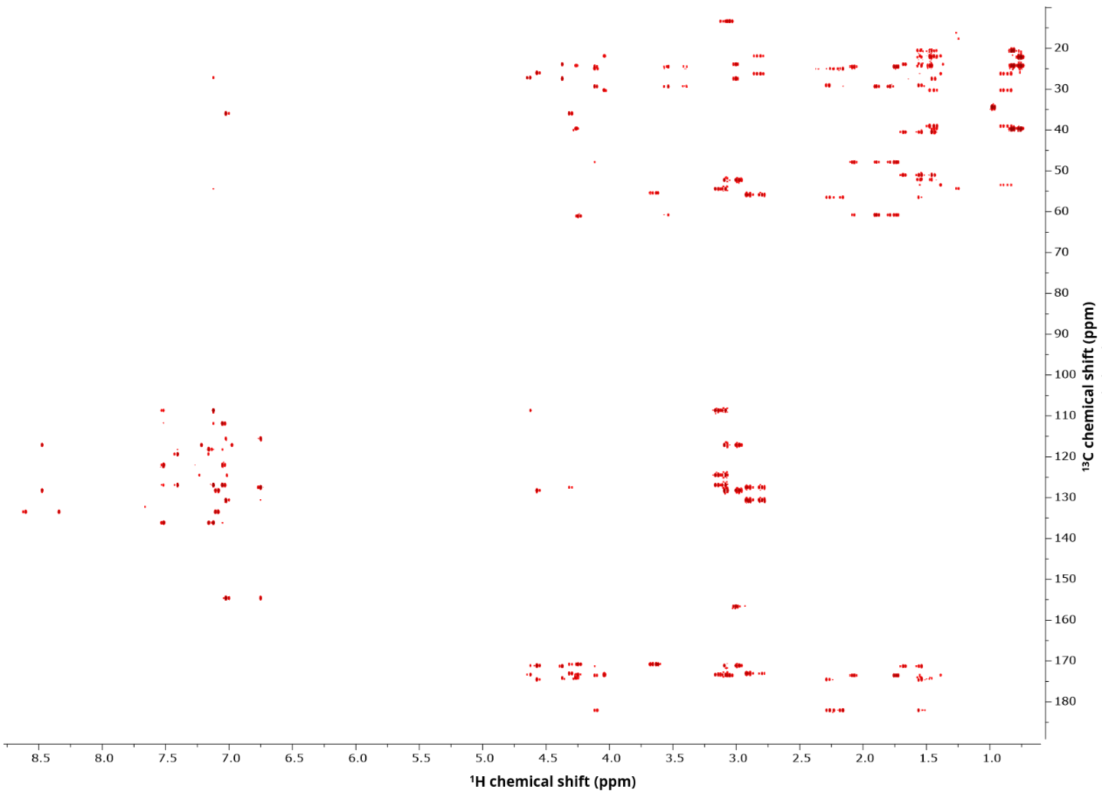

Figure S31: ^{1}H-^{13}C HMBC spectrum of con7 peptide (800 MHz, HDO, 298 K).

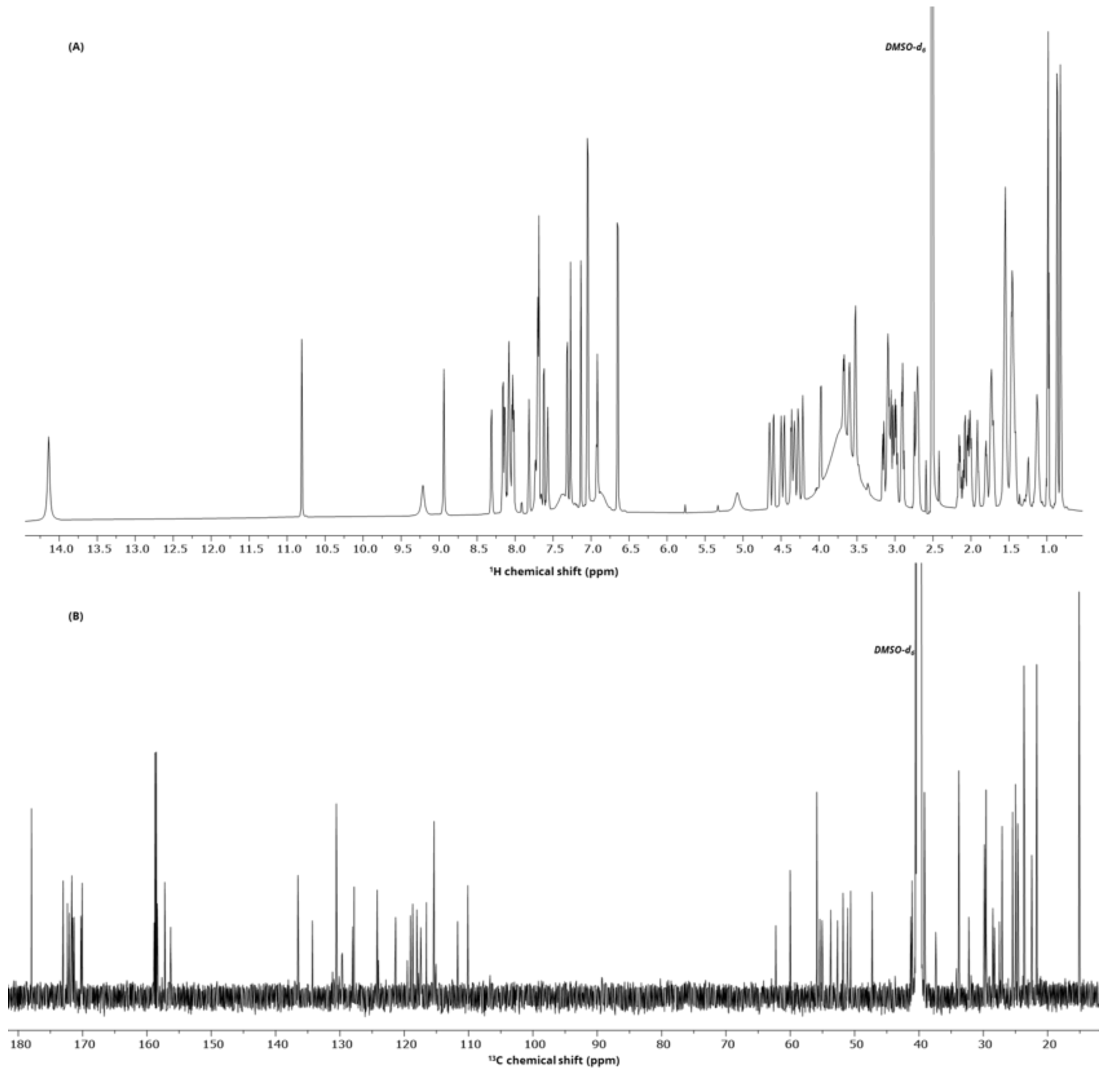

Figure S32: ^{1}H (A) and ^{13}C (B) NMR spectra of con7 peptide (800 MHz, DMSO-d_6, 298 K).

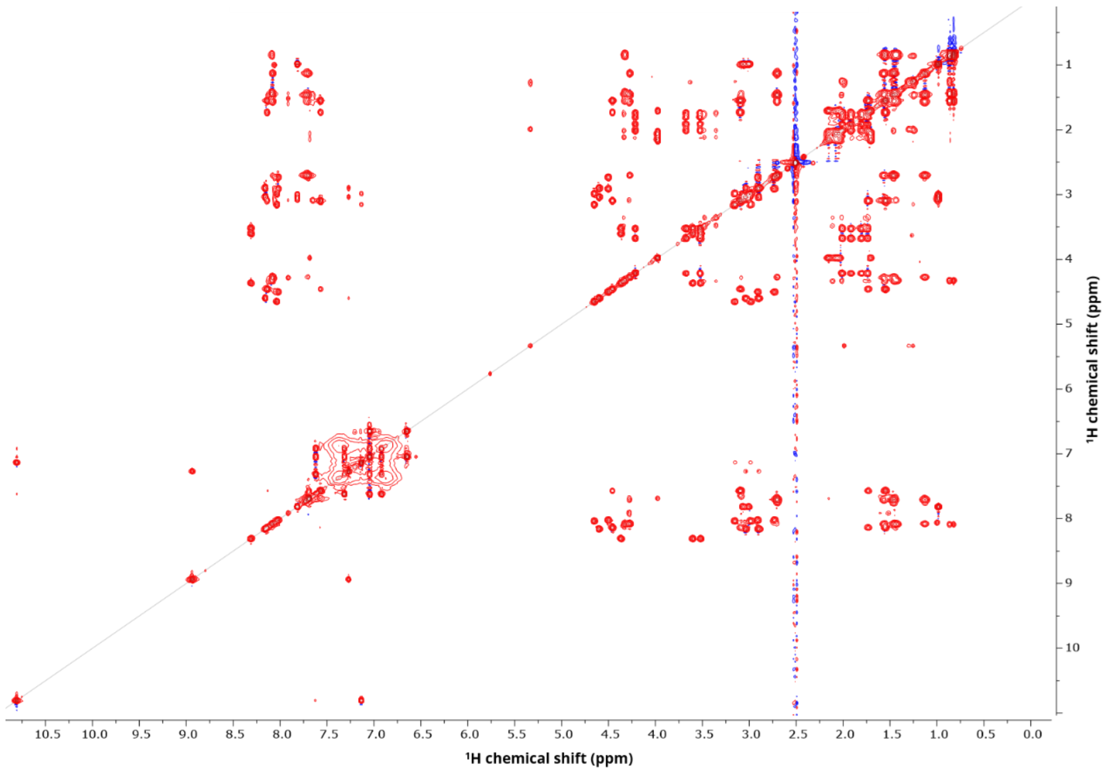

Figure S33: ^{1}H-^{1}H TOCSY spectrum of con7 peptide (800 MHz, DMSO-d_6, 298 K, t_m = 80 ms).

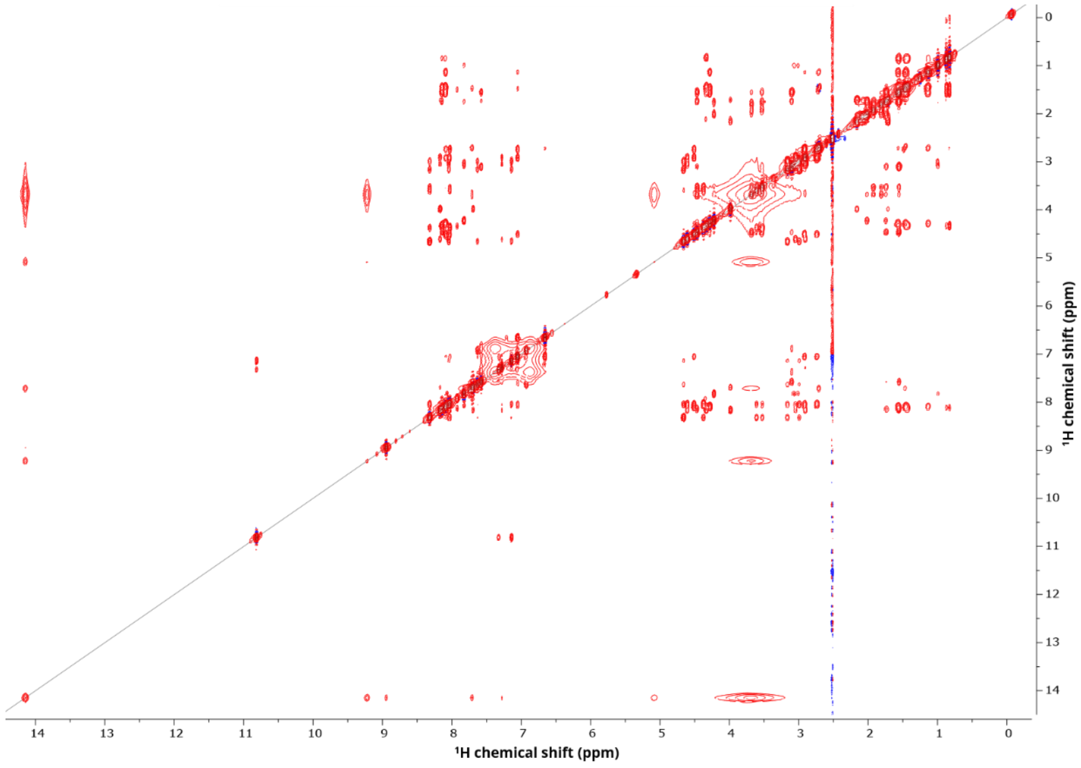

Figure S34: ^{1}H-^{1}H zqf NOESY spectrum of con7 peptide (800 MHz, DMSO-d_6, 298 K, 150 ms).

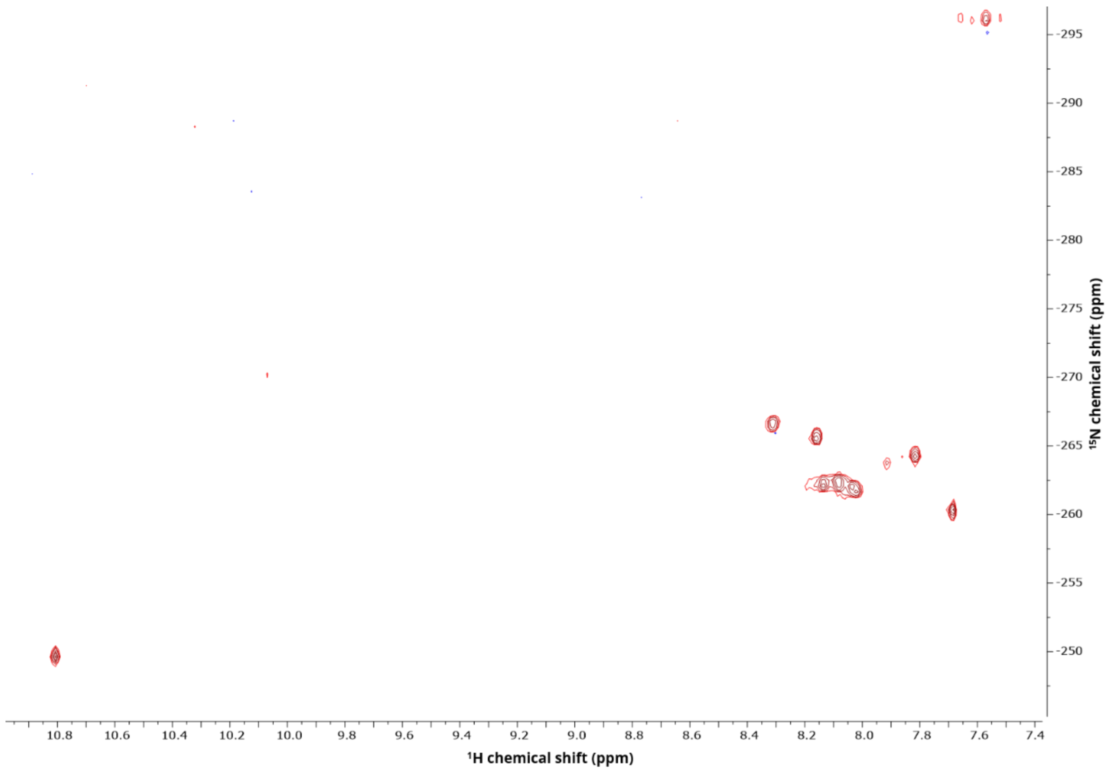

Figure S10: ^{1}H-^{15}N HSQC spectrum of con7 peptide (800 MHz, DMSO-d_6, 298 K).

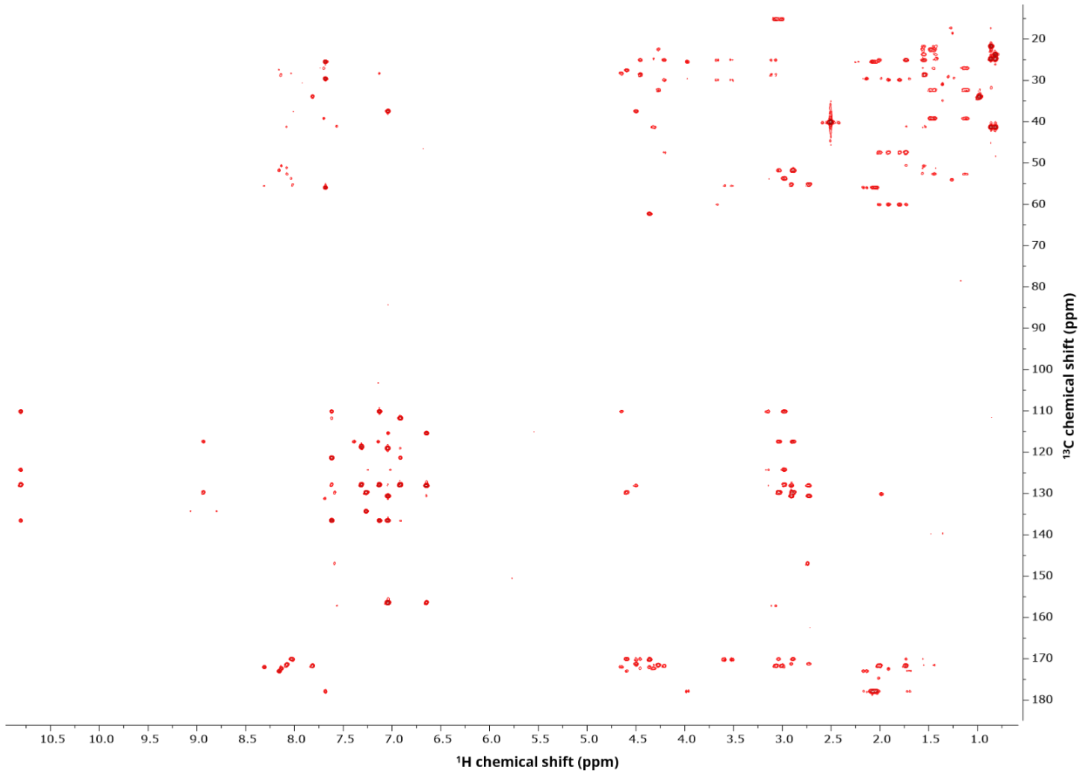

Figure S11: ^{1}H-^{13}C HMBC spectrum of con7 peptide (800 MHz, DMSO-d_6, 298 K).

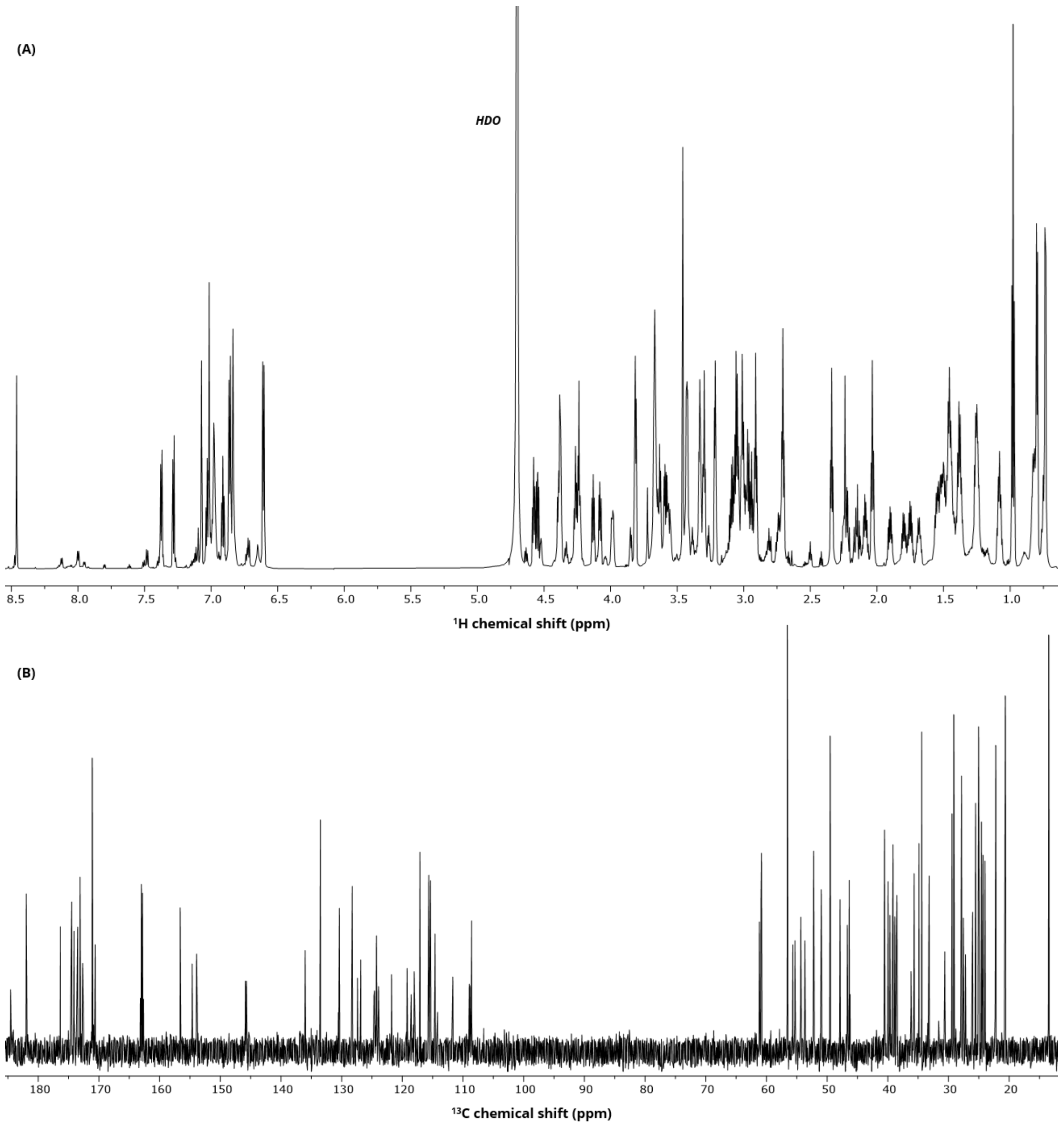

Figure S12: ^{1}H (A) and ^{13}C (B) NMR spectra of con7 (800 MHz, HDO, 298 K).

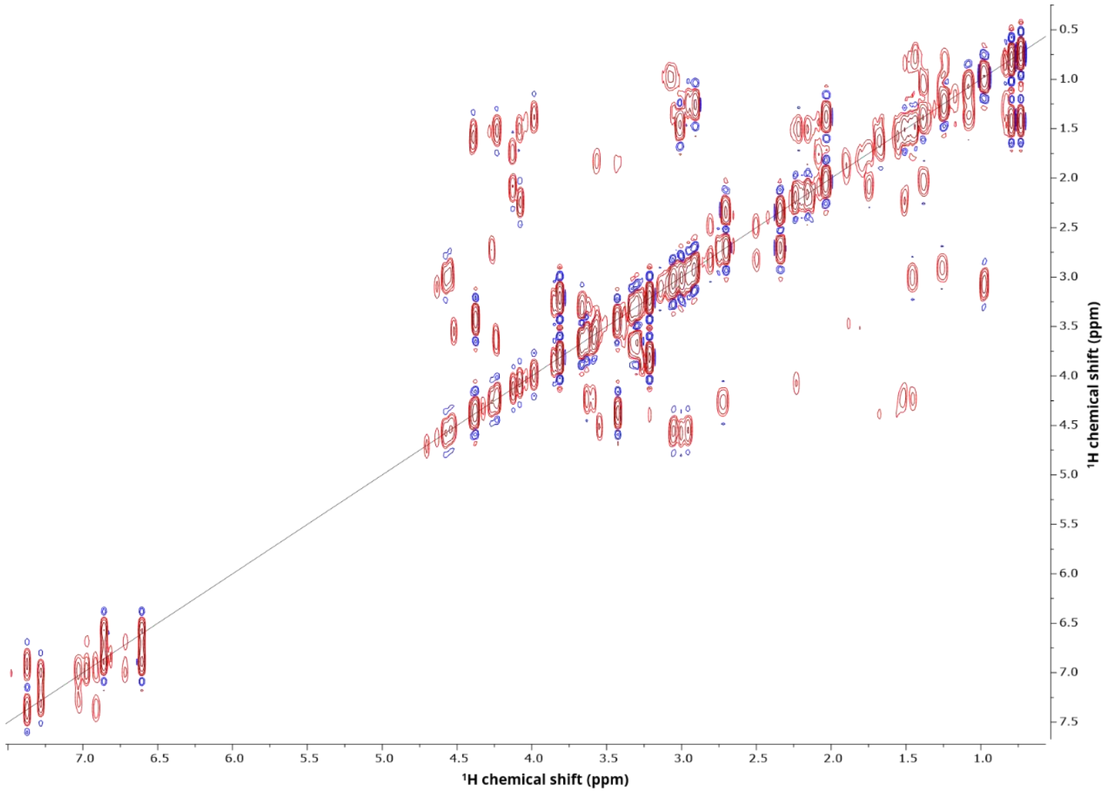

Figure S13: ^{1}H-^{1}H DQF COSY spectrum of con7 (800 MHz, HDO, 298 K).

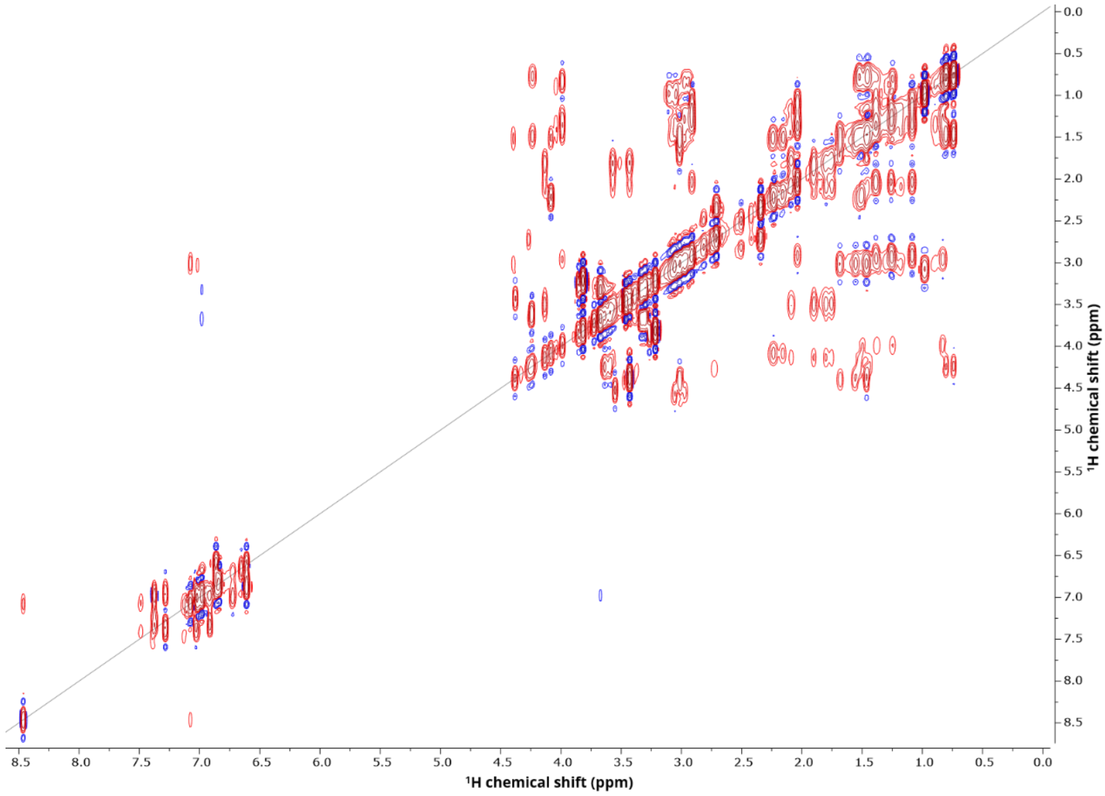

Figure S14: ^{1}H-^{1}H TOSY spectrum of con7 (800 MHz, HDO, 298 K, t_m = 80 ms).

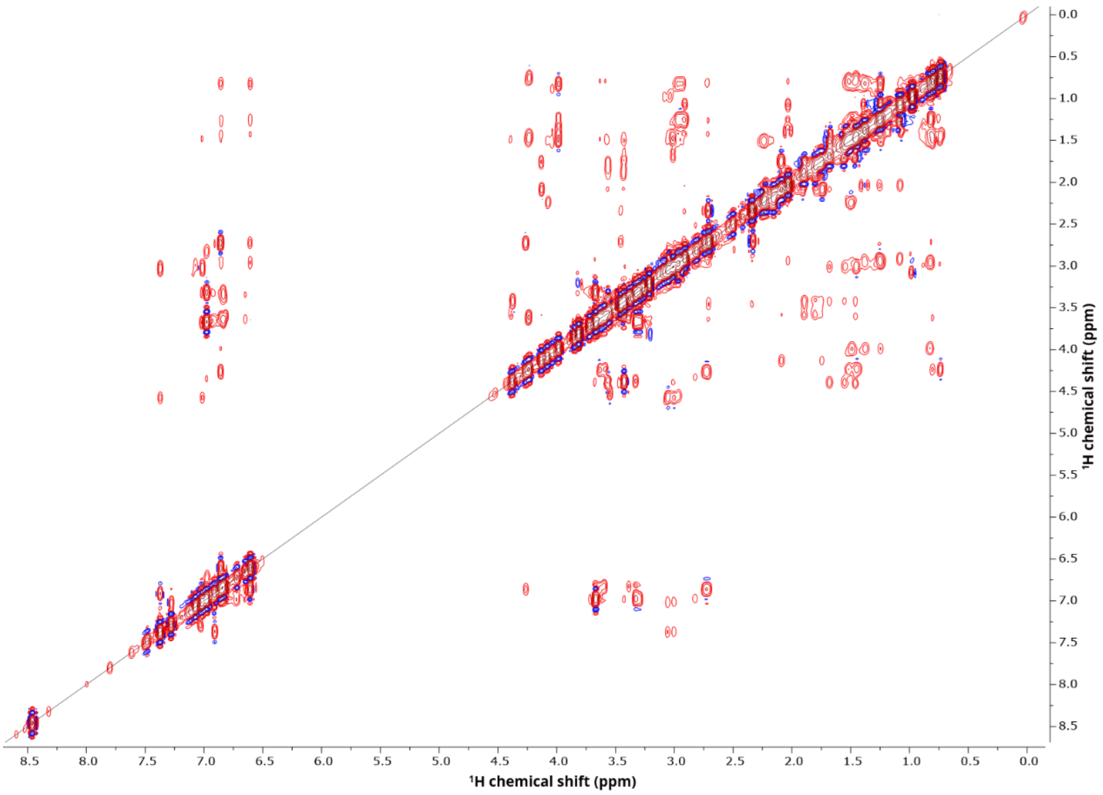

Figure S40: ^{1}H-^{1}H NOESY spectrum of con7 (800 MHz, HDO, 298 K, 300 ms).

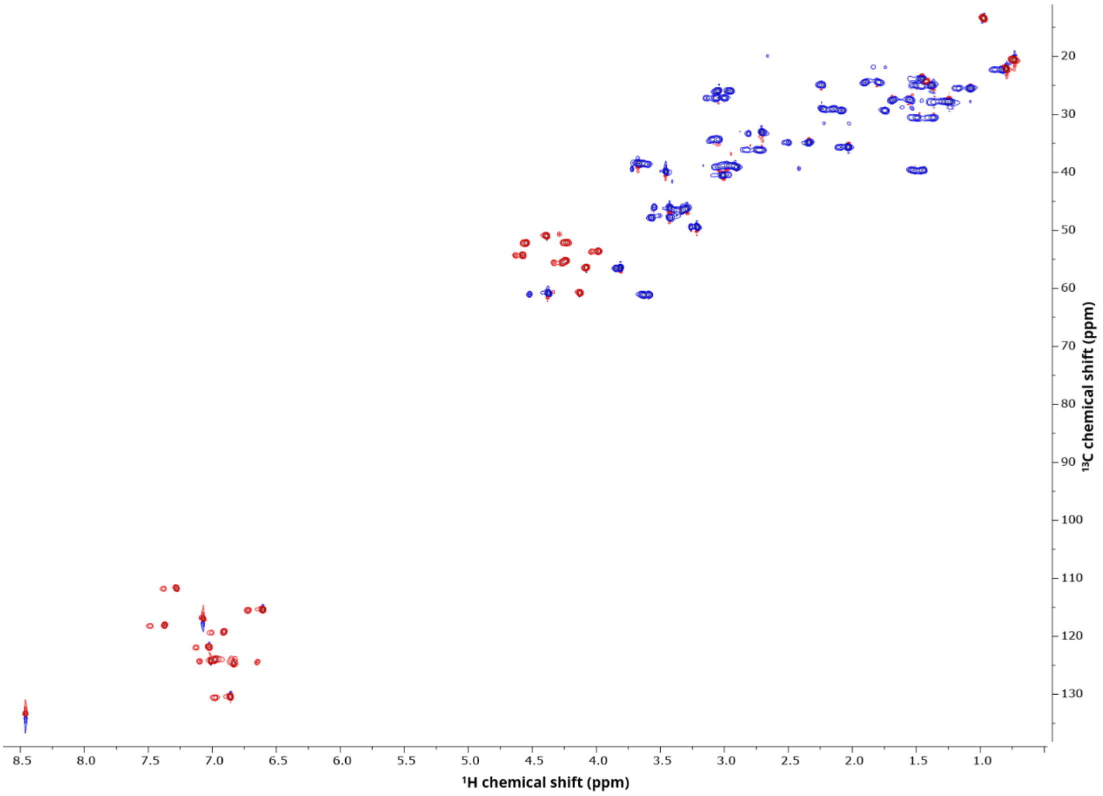

Figure S41: ^{1}H-^{13}C HSQC spectrum of con7 (800 MHz, HDO, 298 K).

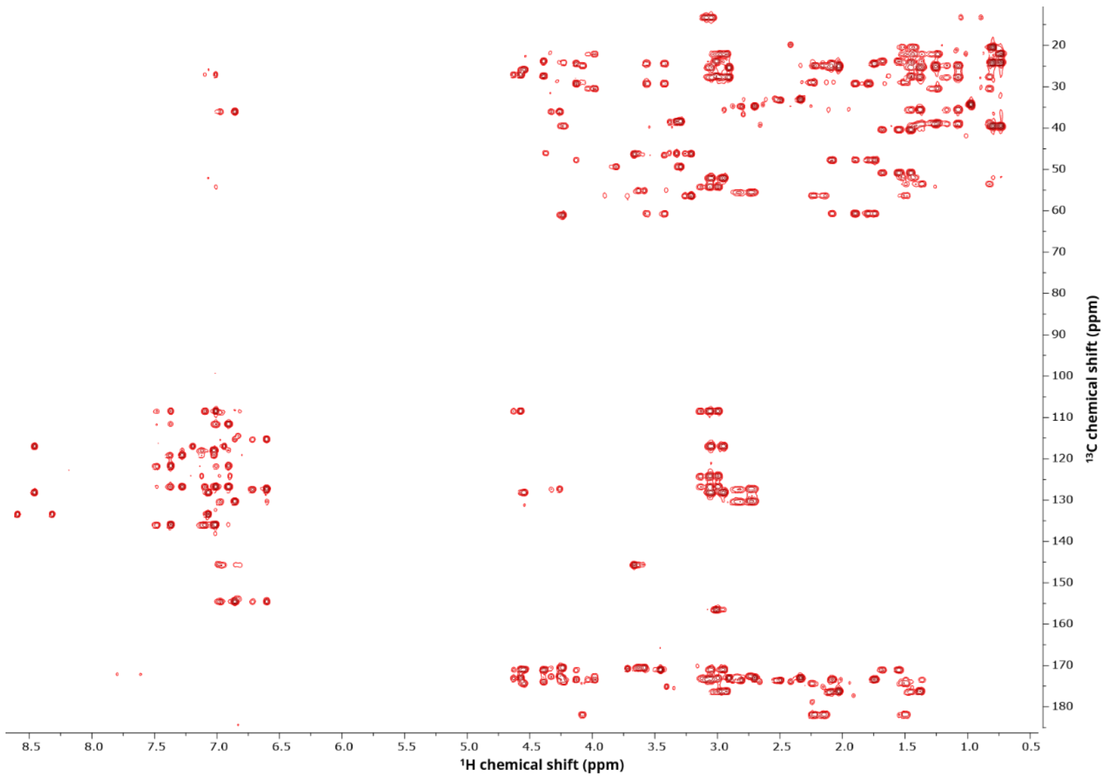

Figure S42: ^{1}H-^{13}C HMBC spectrum of con7 (800 MHz, HDO, 298 K).

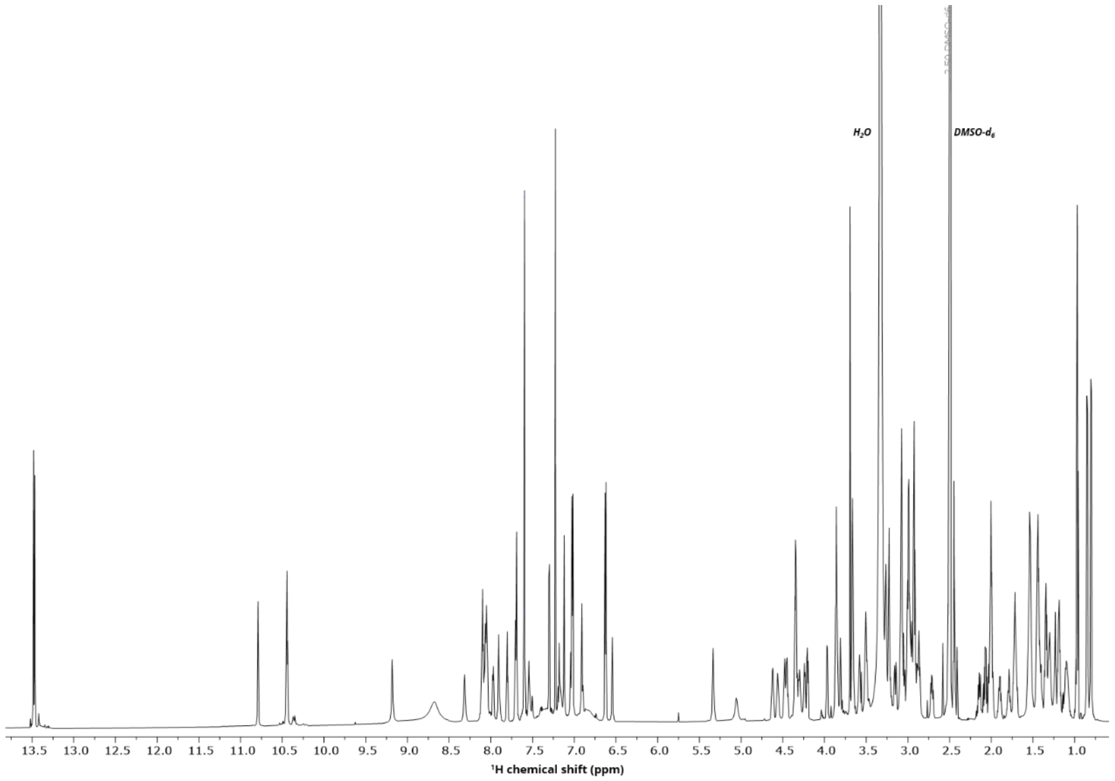

Figure S43: ^{1}H NMR spectrum of con7 (800 MHz, DMSO-d_6, 298 K).

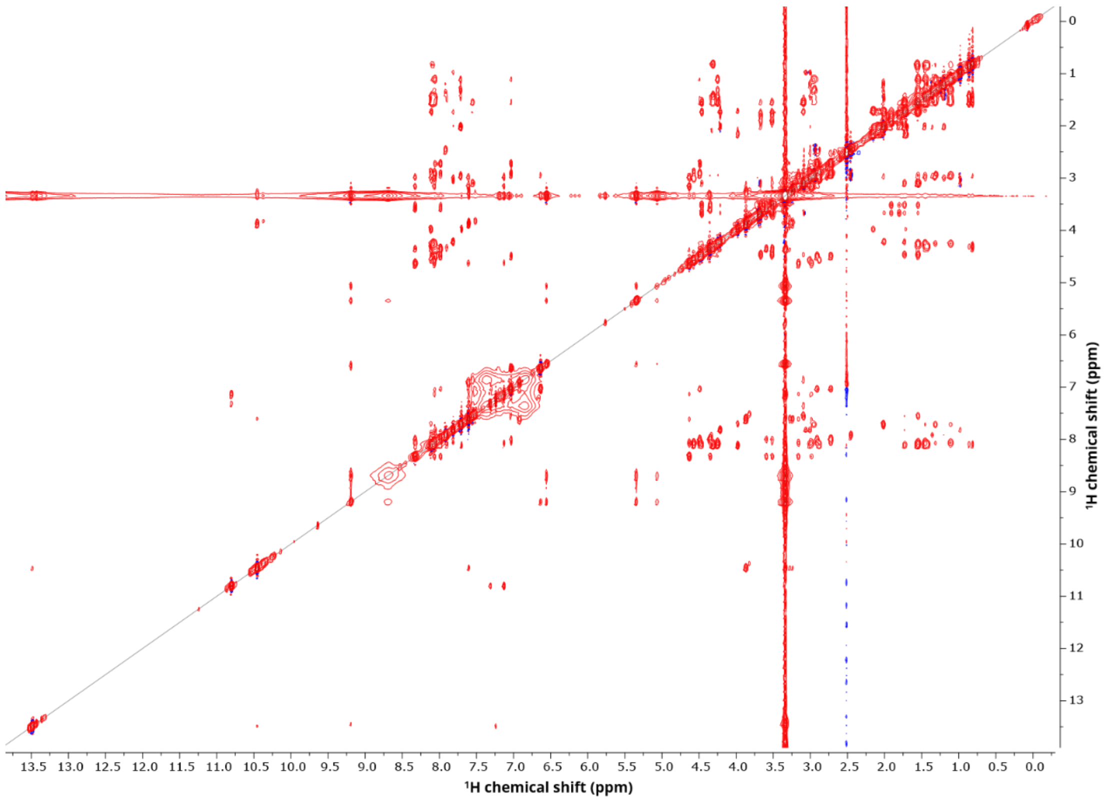

Figure S44: ^{1}H-^{1}H zqf NOESY spectrum of con7 (800 MHz, DMSO-d_6, 298 K, 150 ms).

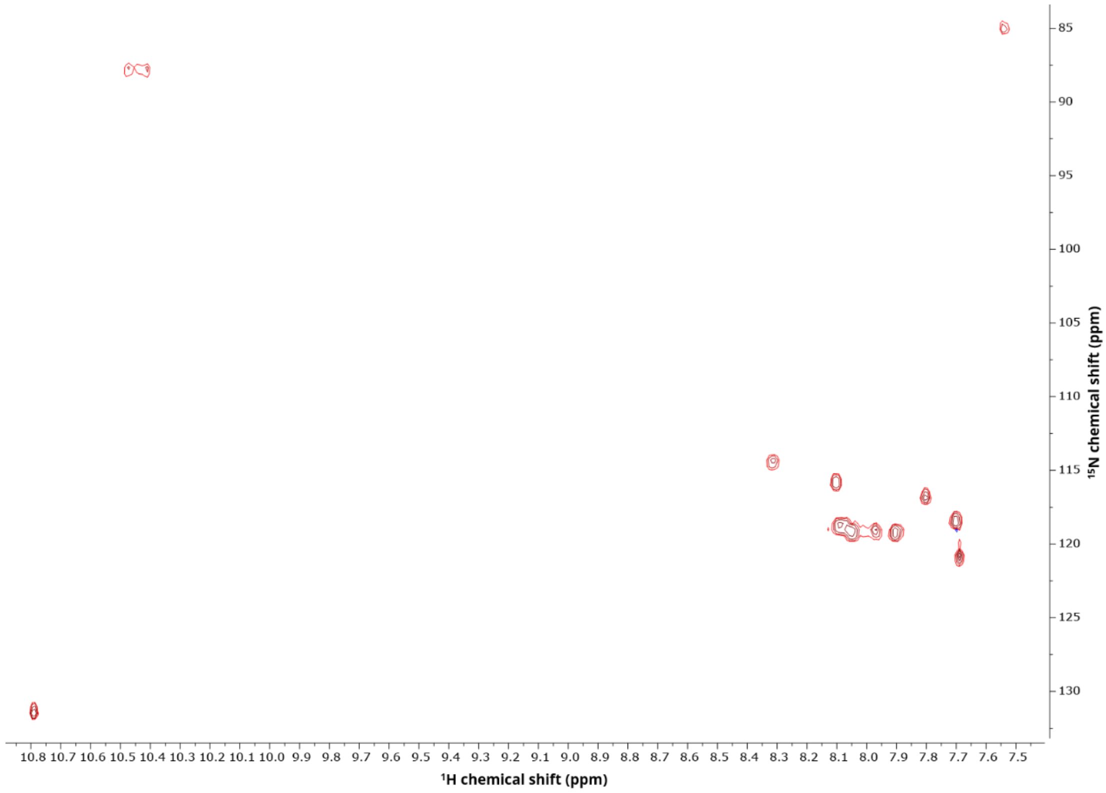

Figure S15: ^{1}H-^{15}N HSQC spectrum of con7 (800 MHz, DMSO-d_6, 298 K).
